# Modeling Within-Host Effects of Drugs on *Plasmodium falciparum* Transmission and Prospects for Malaria Elimination

**DOI:** 10.1371/journal.pcbi.1003434

**Published:** 2014-01-23

**Authors:** Geoffrey L. Johnston, Peter W. Gething, Simon I. Hay, David L. Smith, David A. Fidock

**Affiliations:** 1 Department of Microbiology and Immunology, Columbia University College of Physicians and Surgeons, New York, New York, United States of America; 2 School of International and Public Affairs, Columbia University, New York, New York, United States of America; 3 Bloomberg School of Public Health, John Hopkins University, Baltimore, Maryland, United States of America; 4 Spatial Ecology and Epidemiology Group, Department of Zoology, University of Oxford, Oxford, United Kingdom; 5 Division of Infectious Diseases, Department of Medicine, Columbia University College of Physicians and Surgeons, New York, New York, United States of America; Imperial College London, United Kingdom

## Abstract

Achieving a theoretical foundation for malaria elimination will require a detailed understanding of the quantitative relationships between patient treatment-seeking behavior, treatment coverage, and the effects of curative therapies that also block *Plasmodium* parasite transmission to mosquito vectors. Here, we report a mechanistic, within-host mathematical model that uses pharmacokinetic (PK) and pharmacodynamic (PD) data to simulate the effects of artemisinin-based combination therapies (ACTs) on *Plasmodium falciparum* transmission. To contextualize this model, we created a set of global maps of the fold reductions that would be necessary to reduce the malaria *R*_C_ (i.e. its basic reproductive number under control) to below 1 and thus interrupt transmission. This modeling was applied to low-transmission settings, defined as having a *R*_0_<10 based on 2010 data. Our modeling predicts that treating 93–98% of symptomatic infections with an ACT within five days of fever onset would interrupt malaria transmission for ∼91% of the at-risk population of Southeast Asia and ∼74% of the global at-risk population, and lead these populations towards malaria elimination. This level of treatment coverage corresponds to an estimated 81–85% of all infected individuals in these settings. At this coverage level with ACTs, the addition of the gametocytocidal agent primaquine affords no major gains in transmission reduction. Indeed, we estimate that it would require switching ∼180 people from ACTs to ACTs plus primaquine to achieve the same transmission reduction as switching a single individual from untreated to treated with ACTs. Our model thus predicts that the addition of gametocytocidal drugs to treatment regimens provides very small population-wide benefits and that the focus of control efforts in Southeast Asia should be on increasing prompt ACT coverage. Prospects for elimination in much of Sub-Saharan Africa appear far less favorable currently, due to high rates of infection and less frequent and less rapid treatment.

## Author Summary

We utilize a within-host mathematical model of malaria transmission to predict the effects of antimalarial treatment across the globe. We predict that areas containing 91% of the at-risk population of Southeast Asia can achieve elimination if at least 93–98% of symptomatic individuals are promptly treated with effective artemisinin-based combination therapies (ACTs), based on assessments of treatment and transmission levels as of 2010. The benefit of attaining this level of coverage far outperforms that of adding additional gametocyte-specific transmission-blocking drugs to current ACTs. We advocate for elimination programs in Southeast Asia to focus on maximizing ACT coverage.

## Introduction

*Plasmodium falciparum*, the most virulent of the *Plasmodium* species that cause malaria in humans, is responsible for hundreds of millions of cases per year [1]. The number of fatal outcomes is a matter of considerable debate, with estimates for 2010 ranging from 655,000 to 1,238,000 [2], [3]. Studies nonetheless agree that overall levels of morbidity and mortality have declined over the past decade, due at least in part to the worldwide scaling up of insecticide-treated bed nets and the use of artemisinin-based combination therapies (ACTs). ACTs, which pair fast-acting short-lived artemisinin derivatives with longer-lasting partner drugs, are now the first-line antimalarial drugs in almost the entire malaria-endemic world [4], [5].

Public health and malaria infection experts are increasingly promoting the goal of malaria elimination in areas of low transmission [6], [7] while planning ways to achieve significant reductions in higher-transmission areas [8], [9]. Major obstacles, however, stand in the way. These include insecticide and drug resistance [10], [11], under-developed health care systems, shifting public funding priorities, donor fatigue [6], malaria importation [12] and economic constraints [13]. The complex life cycle of *P. falciparum* also presents unique challenges [14]. *P. falciparum* stages differ markedly in their levels of metabolic activity, within-host locations, and susceptibilities to antimalarials. Mathematical modeling can help guide elimination efforts by providing quantitative predictions to assess the feasibility of different intervention and control strategies [15]–[18].

ACTs and other antimalarial drugs reduce transmission in three ways: by killing the disease-causing asexual blood stages and thus preventing continued production of the intra-erythrocytic sexual gametocyte forms; by killing existing gametocytes and reducing or preventing onward transmission to the mosquito (thereby reducing parasite oocyst numbers in mosquito midguts); and by post-treatment drug prophylaxis wherein residual drug levels can protect against new infections [19]. Here we utilize mathematical modeling to quantify how ACTs reduce malaria transmission, with or without late-stage gametocytocidal agents such as primaquine (PQ) or methylene blue. These agents are receiving considerable interest within the malaria community as to how their action might be leveraged to help interrupt parasite transmission [20]–[22].

Here, we report outputs from our within-host model of *P. falciparum* infection and transmission, and overlay these findings onto geospatial maps of malaria endemicity in order to predict the benefits of extended coverage of infected individuals and incorporation of transmission-blocking agents into current ACT regimens. In low-transmission settings we predict that if at least 93–98% of all symptomatic infections, corresponding to an estimated 81–85% of all infected individuals, were treated within five days of first fever, then the *R*_C_ could be reduced to below one and malaria would progress towards elimination in regions harboring over ∼91% of at-risk populations in Southeast Asia and ∼74% of the global at-risk population. Our findings suggest that increasing treatment coverage with ACTs would be more effective than adding additional transmission-blocking agents in driving towards the goal of malaria elimination in Southeast Asia.

## Results

### A within-host model to predict the effects of drug treatment on reducing malaria transmission

We used our recently developed within-host model of the progression of *P. falciparum* infection [23] to simulate the densities of asexual blood stage parasites and gametocytes in a population of individuals with no acquired immunity to malaria. The variability in densities among individuals was matched to the variability observed in malaria therapy studies, in which syphilitic individuals with no history of malaria infection were infected with *P. falciparum* to induce a fever and clear the syphilis infection [23]. Of note, our model incorporates three different types of antimalarial immunity: an innate response that establishes an upper limit for parasite density; a PfEMP1 variant-specific response that regulates short-term periodic oscillations in density; and a variant-transcending response that causes a steady log-linear decrease in density over time, clearing the infection [23]. In our simulations, these responses were calibrated such that the infection dynamics matched those of experimental challenge volunteers who had no previous malarial infections, i.e., we assumed that individuals either had no prior episode of malaria or had acquired malaria so long ago that their responses were equivalent to that of individuals without prior infections. Further, we simulated only single infections, i.e. we did not simulate infections that overlapped in time.

**Figure 1:**
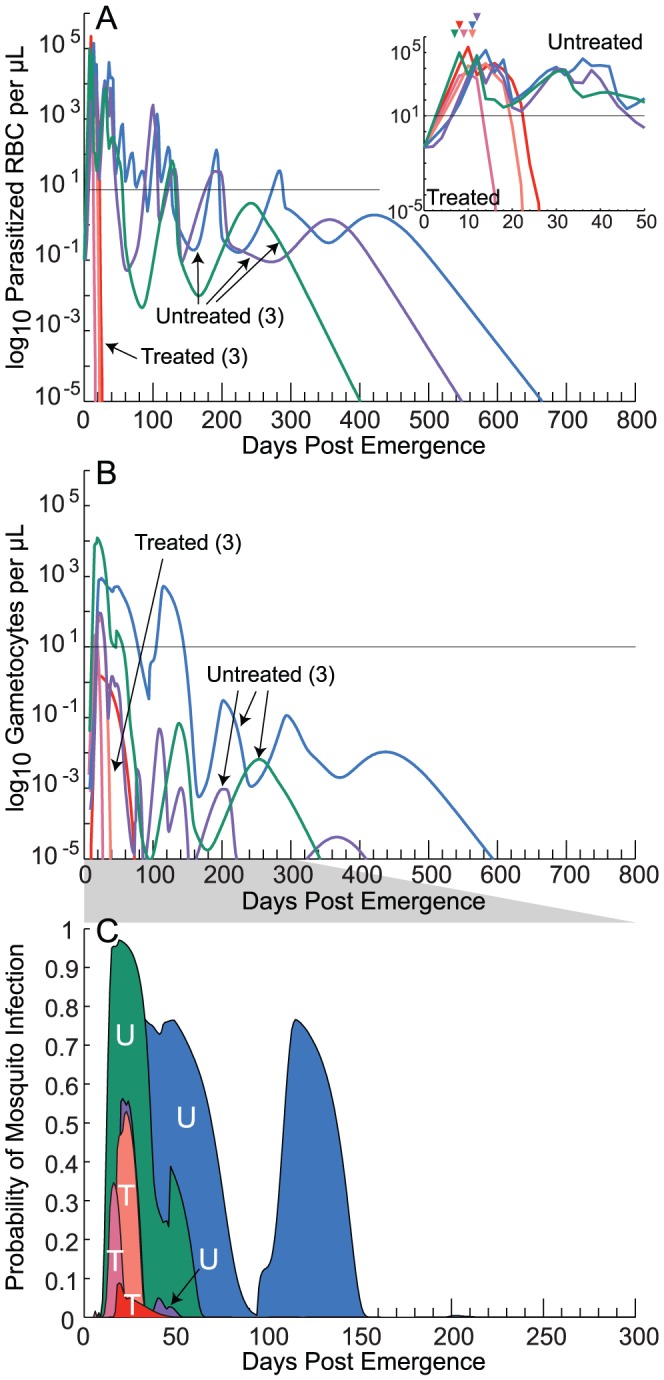
Illustration of asexual, gametocyte, and human-to-mosquito infectivity model outputs. The *P. falciparum* infection model was run six times to simulate three untreated individuals and another three treated with a hypothetical antimalarial. (**A**) Individual log_10_ asexual blood stage parasitemias as a function of the number of days post emergence of parasites from the liver into the bloodstream. The inset depicts the first 50 days of infection; the triangles above indicate the first day of fever. In this example, three individuals were assumed to seek treatment a variable number of days after the onset of fever. The black line illustrates the approximate level of detectability by microscopy (∼10 parasitized red blood cells/µL). (**B**) Daily gametocytemias (sexual stage parasitemias) of the same six individuals. The hypothetical drug treatment was assumed to target early stages of gametocyte development more strongly than later stages. (**C**) Estimated probability of human-to-mosquito parasite transmission for treated (T) vs. untreated (U) individuals. Areas under the infectivity curves (AUIC) are equivalent to the number of fully infectious days. Net infectivity simulations yielded 1.0, 2.4, and 6.3 fully infectious days for treated (T) and 67.7, 6.5, and 28.6 days for untreated (U) individuals, respectively. The model outputs for untreated patients shown in panels A–C were previously reported in [23] and are shown to compare with our modeling of treated patients.

**Figure 1** illustrates six runs from our within-host infection model; untreated individuals are denoted by ‘Untreated’ or ‘U’. **Figure 1A** shows the log_10_ parasitized red blood cells (PRBC) per µL, while **Figure 1B** depicts the daily gametocytemias over time, which are typically two logs lower for untreated individuals. The **Figure 1A** inset illustrates the asexual densities for the first 50 days post emergence of parasites into the bloodstream; colored triangles illustrate the onset of first fever [23]. Once the daily gametocytemias were simulated, a gametocyte density-to-infectivity relationship was utilized to translate these values into predicted infectiousness to mosquitoes over time. **Figure 1C** illustrates the predicted human-to-mosquito infectivity for each of the three untreated individuals (U) using the Jeffery-Eyles (JE) relationship between gametocyte densities and infectivity [23].

As described in detail below, we then incorporated the effects of drug treatment into our within-host model to illustrate how this modeled treatment affects malaria transmission. **Figure 1** includes the results of a hypothetical drug treatment on three individuals infected with *P. falciparum*; treated individuals are denoted by ‘Treated’ or ‘T’. As with the untreated cases, **Figure 1A** shows the asexual parasite densities in log_10_ PRBC/µL, while **Figs. 1B and 1C** depict the daily gametocytemias and human-to-mosquito infectivities. Treatment was assumed to begin 4, 9 and 14 days after the onset of fever. The effects of drug treatment can be seen immediately on the asexual blood stage population, which showed a steep drop in numbers after dosing, as well as the gametocytemias that were lower among the treated individuals following treatment. To calculate the net infectivity of individuals to mosquitoes, gametocytes densities were transformed into infectivity probabilities, and the area under the infectivity curve was derived (AUIC). This approach was previously used with field data [24] to estimate onward infectivity following treatment. In the case where drugs reduced oocyst numbers independently of their effects against gametocytes, the gametocyte density-to-infectivity relationships were adjusted in a drug-dependent manner. Infectivities of treated patients (T) were of relatively brief duration and had largely disappeared within 30 days (**Figure 1C**). This hypothetical example illustrates many of the processes involved in modeling the effects of drugs on transmission. Below we describe how we have used *in vitro* and field data to parameterize the various components of drug activity and predict the effects of various antimalarial therapies in real-world settings.

### Modeling drug effects against asexual blood stage *P. falciparum* parasites

To model the effects of different drugs on asexual parasite densities, we first modeled the within-host concentrations of the partner drugs of two ACTs, artesunate+mefloquine (AM) and artemether+lumefantrine (AL). AM is a frequently used first-line therapy in parts of Southeast Asia [24], while AL has recently become the most widely-used ACT worldwide [25]. Because we calculated our asexual parasite densities daily, we did not model the explicit concentrations of the artemisinin derivatives, as these drugs have half-lives of 1 to 3 hr [4]. However, we did incorporate their fast-acting PD potency on asexual blood stage parasite densities [4]. As a point of reference, we also modeled the drug concentrations of chloroquine (CQ), the former first line therapy that is highly active against asexual blood stages and has some activity against very early stage gametocytes but is inactive against mature gametocytes [21]. For lumefantrine (LMF) uptake and clearance we used a two-compartment pharmacokinetic model parameterized from field data; for mefloquine (MFQ) and CQ we used two-compartment non-parametric models, also parameterized from field data. Model equations, model parameters, and a description of the model fitting to average concentrations as well as population variation are detailed in **Text S1**. Notably, our study assumed that individuals were fully compliant with treatment and that parasites were sensitive to the various drug combinations. In future work we hope to examine how differences in patient compliance and parasite drug susceptibility impact transmission.

**Figure 2:**
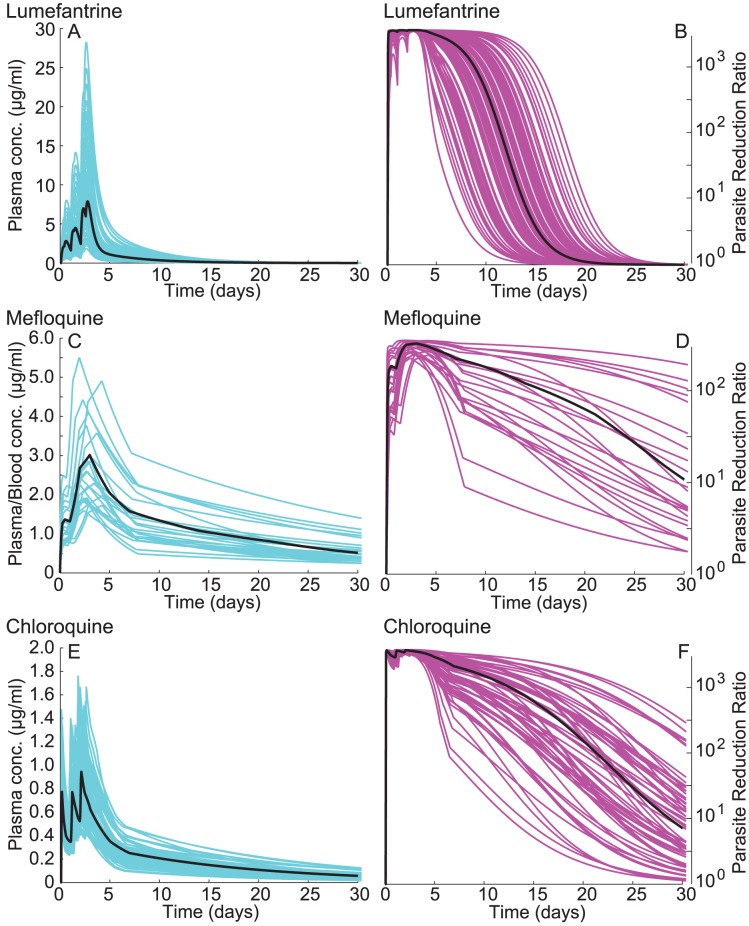
Modeled pharmacokinetic and pharmacodynamic profiles of lumefantrine (LMF), mefloquine (MFQ) and chloroquine (CQ) against asexual blood stage parasites. (**A**) Modeled plasma concentrations of LMF. The wide variability in concentrations reflects individual differences in absorption and clearance. The black line indicates population median drug concentrations. (**B**) Estimated parasite reduction ratios (PRR) over a 48 hr blood stage cycle for LMF as a function of time post onset of treatment. PRR is the fold reduction in asexual parasite densities due to drug action. PRR values were calculated from drug concentrations using a Hill function transformation. Each curve illustrates the PRR over time for a simulated individual; the black line illustrates the PRR for population median drug concentrations. (**C–D**) Plasma concentrations for MFQ and corresponding activities against asexual blood stage parasites. Drug concentrations reflect data from both plasma and whole blood studies. (**E–F**) Modeled plasma concentrations of both CQ and its metabolite monodesethyl-chloroquine (mdCQ) are shown in turquoise; PRR against asexual blood stages are in purple. All drug concentrations are in µg/ml.

**Figure 2**** (panels A, C, E)** illustrates the results of our PK simulations for LMF, MFQ, and CQ, respectively. The black lines indicate the median (LMF) or mean (MFQ, CQ) of the population concentrations, while the blue lines indicate simulated individual concentration profiles. Model outputs revealed wide variations in concentrations within a population, due to differences in rates of drug uptake and clearance. The number of concentrations depicted in each panel corresponds to the number of patients in each of the studies that provided data for model fitting. Of note, LMF plasma concentrations achieved considerably higher levels than the other two agents.

To simulate the effects of these drug concentrations against asexual parasites, we first calculated hourly plasma and/or blood concentration levels from our PK modeling. We then translated the hourly drug concentrations into asexual activities, assuming that the dose-response relationships could be modeled as Hill functions. These Hill functions were parameterized from *in vitro* and field data (see the **SI**). The asexual activities of drugs were quantified as 48-hour parasite reduction ratios (PRRs), i.e., the fold decreases in parasite numbers every 48 hr, corresponding to one cycle of intra-erythrocytic development and reinvasion. **Figure 2** **(panels B, D, F)** illustrates the drug concentrations translated into these PRRs over time. To calculate the asexual parasite densities in our model under the effects of drugs for a generic day *t*, we took the densities from day *t*-1, applied our within-host model to calculate densities on day *t* including both the effects of parasite growth and host immune responses, then multiplied densities by the square root of the mean 48-hour PRR for drug concentrations during day *t*. The resulting density was then used to calculate densities at *t*+1, and so on until the end of the simulation.

Regardless of the drug regimens simulated here, asexual parasite densities fell rapidly when an individual was treated. The PRRs nevertheless showed substantial differences between drugs in later time periods following treatment. For example, the LMF PPRs rapidly declined within 5–15 days of treatment, whereas CQ took longer to decline while showing more heterogeneity. MFQ was also heterogeneous but always showed lower PPRs even at peak plasma concentrations. The maximal PRRs, and the differential rates of absorption, clearance, and volumes of drug distribution are described more fully in the **SI**.

### Predicted effects of antimalarials on gametocyte development and transmission

While effective drug treatment rapidly clears asexual blood stage parasites, even successful regimens differ markedly in their effects on gametocytes. As gametocytes develop, they become metabolically more inert, thus reducing the number of drug targets available. Specifically, gametocytes mature over the course of ∼10–15 days through Stages I–V, with each stage differing in metabolic activity and drug susceptibility [26]. Stages I–IV are sequestered in the microvasculature, spleen or bone marrow and are not present in the blood circulation. Only Stage V gametocytes have matured to the point of releasing from sequestered sites, thus becoming visible by blood smear. Here, we simulated differential effects of drugs against each developmental stage. This feature is novel to the literature so far as we know, although a recent article did allow for a differential effect on non-circulating vs. circulating gametocytes [27].

To parameterize the modeled effects of drugs on gametocytes, we first conducted a literature review to examine the types of datasets that could inform the model. There are few *in vitro* studies that have measured the stage-specific effects of drugs on gametocytes [21]; however, many field studies have examined the clearance of gametocytes within a population after drug treatment. These studies typically measured the proportion of individuals who were gametocytemic by microscopy after treatment (threshold for detection: ∼5–10 gametocytes per µL [28], [29]).

**Figure S1** illustrates the prevalence of gametocytes from field studies of patients treated with a variety of antimalarials [28], [30]–[38]. The field studies are disaggregated by drug type: treatment with SP [28], [30], [33]; CQ or amodiaquine (AQ) (sometimes in combination with SP) [28], [30]–[33]; various ACTs [28], [30]–[32], [34]–[36], [38]; or ACTs plus PQ [35], [36], [38]. The inter-study variability observed in **Figure S1** was likely due to a variety of factors, including the levels of acquired immunity in the population, exact timing of treatment, age of treated individuals, differences in parasite biology, and drug treatment regimens.

**Figure 3:**
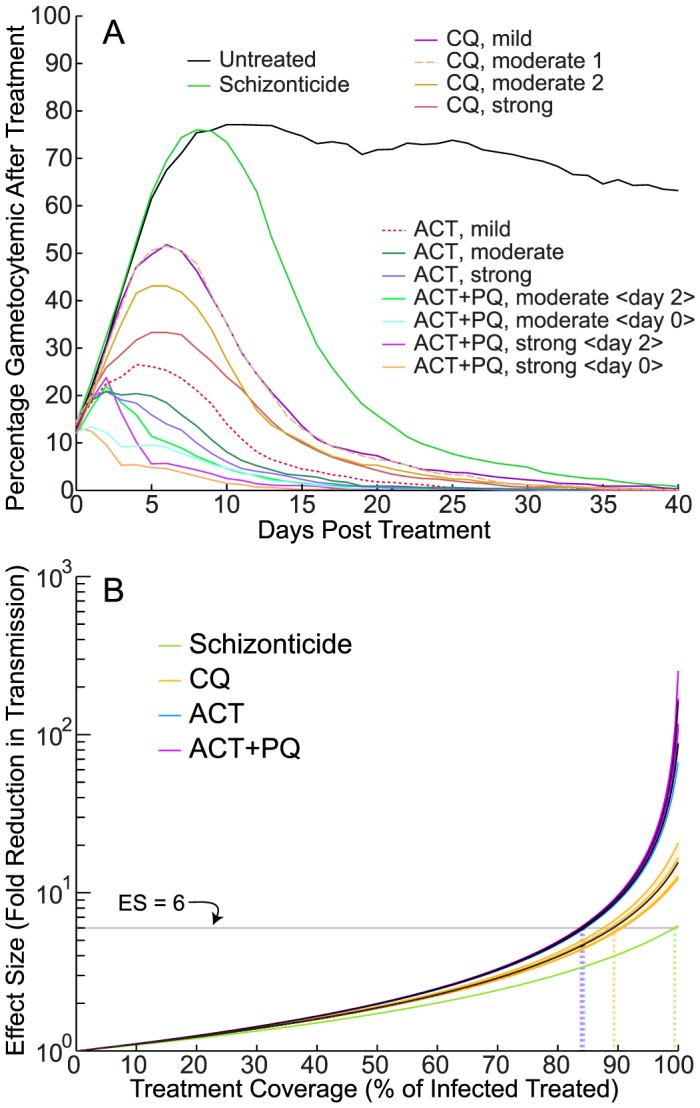
Modeled post-treatment gametocyte prevalence and treatment effect sizes. Treated and untreated malaria infections were simulated using our within-host malaria infection model. Modeled treatments differed according to the assumed level of gametocyte killing. Model treatment was assumed to start 5 days after the first onset of fever; all model outputs represent the mean of 1,000 runs. (**A**) The number of individuals predicted to be gametocyte positive by microscopy (threshold 5 gametocytes per µL) was tracked over time. Untreated model outputs are shown in black. Treatment was assumed to be a combination therapy with a short-lived component (active for 3 days) and a longer-lived component with the pharmacokinetic profile of mefloquine. The green line illustrates the effects of treatment assuming no gametocytocidal activity (‘Schizonticide’). The assumed gametocytocidal activity of each component was progressively increased and compared to field data to generate the rest of the curves, each labeled with their corresponding antimalarial (chloroquine, CQ; artemisinin-based combination therapy, ACT; primaquine, PQ). The curves labeled ‘ACT+PQ’ assumed the presence of a second short-lived partner that strongly killed both early and late stage gametocytes; the number indicates the day on which the simulated PQ component was administered. (**B**) Total effect sizes (fold-reductions in transmission) for each of the modeled drug parameterizations as a function of treatment coverage, including the oocidal effects of drugs, assuming net untreated infectivity of 30.5 days, and using the ‘Jeffery-Eyles’ density-to-infectivity parameterization for treated individuals (**Table 2**; [23]). Each drug class is depicted in a different color. The variation in each class is due to the different simulated levels of gametocytocidal activity for that drug type. Each line within a given drug class represents the result of 1,000 simulation runs; the black lines indicate the mean effect sizes for each class of drug. The horizontal line illustrates a six-fold reduction in transmission. The dotted vertical lines indicate the levels of treatment coverage needed to reach a six-fold reduction in total human-to-mosquito transmission for each drug class. The y-axis is in log-scale.

These data were used to calibrate the gametocyte component of our drug effects model. To generate a set of model outputs to compare against field data, we simulated treating individuals with a three-day course of AM and varied the assumed killing properties of the two component drugs. We assumed that treatment started relatively early in the infection, i.e., 5 days after first fever, in agreement with field studies from Thailand and Indonesia [34], [38]. Treatment timing will vary from place to place given the treatment-seeking behaviors of the local population; if treatment is delayed significantly beyond this point, the ability of drugs to reduce transmission is likely to be diminished. We began our simulations assuming that the treatment had no effect on gametocytes at all; we called this therapy purely schizonticidal. We then increased the simulated killing properties of the combination, assuming that the components only killed early stages of gametocytes (e.g. as for CQ). We gradually increased this presumed killing power against early gametocytes (from mild to varying levels of moderate to strong, as in **Figure 3A**): as the killing power increased, gametocyte prevalence post-treatment decreased. We then assumed that the short-lived component killed both early and late stage gametocytes, with a larger effect on the former (e.g. as for an ACT). Finally, we simulated adding a single dose of a third drug that killed both early and late stage gametocytes, with varying levels of activity (e.g. as for ACT+PQ). Additionally, we varied the timing of this third component as this has been a topic of debate [39], by assuming that treatment occurred either on the first day of ACT treatment, denoted by ‘<day 0>’, or last day, denoted by ‘<day 2>’. Our studies used the simulated prevalence of post-treatment gametocyte positive individuals as the output to be compared against field data, since this metric was most often tracked in the field.

Once the model outputs had been generated, these were compared to field studies of drug treatments with similar activity (see **Figures S1, S2**): the modeled schizonticidal treatment data were compared to field trials with SP; mild to moderate gametocytocidal outputs were compared to data from field trials of CQ, CQ+SP or AQ+SP; strong gametocytocidal outputs were compared to ACT clinical trials data, and the modeled triple-combination data were compared to field data for ACTs+PQ. For each drug activity type we then chose the sets of simulations that most closely resembled the mean, maximum, and minimum of observed responses to represent the effects of each class of drugs against gametocytes. We also included some intermediate sets of simulations for the sake of comparison. **Figure S2** illustrates the model outputs that best approximated the mean and observed variation in the field data; all model means were from 1,000 runs for each parameterization. We modeled the entire range of observed variation in post-treatment gametocytemias to allow for sensitivity and robustness analyses in our results. This ‘ensemble modeling’ approach has been used previously to model the effects of vaccines on malaria transmission [40] as well as within-host *P. falciparum* dynamics [41].

**Figure 3A** illustrates the results of the gametocyte activity model fitting. The untreated model gametocyte prevalence 5 days after first fever is shown in black. The modeled post-treatment gametocyte prevalence assuming a pure schizonticidal combination is shown in green. The four model parameterizations that best correspond to the observed field patterns after CQ treatment are labeled ‘CQ mild’, ‘CQ moderate 1’, ‘CQ moderate 2’, and ‘CQ strong’, respectively (indicating increasing levels of activity against very early stage gametocytes). The three model parameterizations that best correspond to the ACT field patterns are labeled ‘ACT mild’, ‘ACT moderate’, and ‘ACT strong’, respectively. The four model parameterizations that correspond to the ACT+PQ field studies are labeled ‘ACT+PQ moderate <day 0>’, ‘ACT+PQ moderate <day 2>’, ‘ACT+PQ strong <day 0>’, and ‘ACT+PQ strong <day 2>’; the bracketed number indicates the day on which the simulated PQ component was administered, relative to the other two drug components.

Once the gametocyte parameterizations were fitted for each type of drug combination, we then transformed the daily gametocytemias before and after treatment into predicted infectivities to mosquitoes. These transformations utilized gametocyte density-to-infectivity relationships derived from mosquito feeding studies, as described in [23]. We chose two transformations, one derived from studies of mosquito feeding on malaria therapy patients with no prior history of malaria infection (‘Jeffery-Eyles’ or JE) and the other derived from feeding studies conducted in field trials in Africa (‘Carter & Graves’ or CG). In short, the JE transformation assumes that 1) gametocytes appearing in the first few days of infection are non-infectious (immature); 2) low density gametocytemias are relatively non-infectious; 3) high density infections are highly infectious. The CG relationship assumes that 1) gametocytes are immediately infectious; 2) low density gametocytemias are relatively infectious; 3) high density infections are not as infectious as for JE [23]. These two functions are substantially different and represent some of the possible types of density-to-infectivity relationships. Calculation using both relationships allows us to highlight where differences in density-to-infectivity assumptions play an important role in interpreting model outputs [23]. It was assumed for both parameterizations that modeled gametocytemias were infectious at densities below the level of detection by microscopy (∼5–10 gametocytes per µL). Our model is thus able to capture the effects of ‘submicroscopic’ infections [23], [35], [42]. However, as densities decrease, infectivity decreases asymptotically toward 0 (see **Figure 3** of [23]). As a simplification we assumed that gametocyte densities below 2 gametocytes per 3 µL were non-infectious (given the need for 2 gametocytes per mosquito bite that typically collects ∼3 µL of blood) [23].

**Figure S3** illustrates the modeled gametocytemias of **Figure 3A** transformed into probabilities of mosquito infection, along with data from feeding studies in the field [28], [30]–[33], treatment with various ACTs [28], [30]–[32], [34]–[36], [38], and treatment with ACTs plus PQ [35], [36], [38]. Both the JE and CG transformed probabilities are shown; the data transformed using the JE assumptions are in bold. The coloring of modeled infectivity data corresponds to that of **Figure 3A**. The field feeding study data were disaggregated according to the same criteria as the gametocyte clearance data.

Once the model gametocyte and infectivity parameters were fitted to data, we then calculated the AUIC for each drug parameterization [23]. **Table 1** provides the unadjusted net human-to-mosquito infectivity for each of the drug parameterizations in **Figure 3A**. All data are from the mean of 1,000 model runs. To determine the transmission reduction achieved with each treatment, we divided the untreated AUIC by the treated AUIC. For example, to calculate the transmission reduction post-treatment, we first took untreated individuals and calculated the mean AUIC for 5 days after first fever until the end of simulation; the mean untreated AUIC values were 31.8 for the JE parameterization and 29.3 for the CG parameterization (**Table 1**). The post-treatment net infectivities of treated individuals, i.e. the treated AUIC values, were 0.70 for JE and 0.94 days for CG (**Table 1**). Mean fold reductions in transmission, post ACT treatment, were then 47.1 and 32.4 for JE and CG transformations, respectively (**Table 1**).

**Table 1:** Modeled net infectivities and effect sizes of antimalarial treatment.

	Pre-treatment	Untreated	Schizont-icide	CQ					ACT				ACT+PQ				
Parameterization				mild	mod 1	mod 2	strong	mean	mild	mod	strong	mean	mod <0>	mod <2>	strong <0>	strong <2>	mean
Net Infectivity (JE)	0.03	31.8	**5.12**	2.57	2.47	1.88	1.51	**2.11**	0.90	0.66	0.55	**0.70**	0.32	0.39	0.15	0.26	**0.40**
Net Infectivity (CG)	3.06	29.3	**5.16**	2.81	2.73	2.20	1.78	**2.38**	1.20	0.89	0.74	**0.94**	0.48	0.60	0.26	0.44	**0.58**
Fold-reduction (post-treatment, JE)			**6.2**	12.4	12.8	16.9	21.1	**15.8**	35.3	48.1	57.9	**47.1**	100.3	81.1	208.6	120.2	**101.7**
Fold-reduction (post-treatment, CG)			**5.7**	10.4	10.8	13.4	16.5	**12.8**	24.5	32.8	39.8	**32.4**	61.3	48.7	112.7	66.5	**59.7**
Effect size, JE			**6.2**	12.3	12.7	16.7	20.7	**15.6**	34.3	46.3	55.4	**45.3**	92.8	76.2	178.4	109.6	**92.2**
Effect size, CG			**3.9**	5.5	5.6	6.2	6.7	**6.0**	7.6	8.2	8.5	**8.1**	9.2	8.8	9.8	9.3	**8.9**

**Abbreviations**: JE, Jeffery-Eyles; CG, Carter & Graves; CQ, chloroquine; ACT, artemisinin-based combination therapy; PQ, primaquine. Net infectivity indicates the area under the infectivity curve, a measure of the malaria transmission potential. This value indicates the mean number of days that an individual is infectious for a mosquito vector. Fold-reductions indicate the fold changes in net infectivity either post-treatment or pre- and post-treatment. The latter change is known as the ‘effect size’ of an intervention. The modeled parameterizations for CQ are mild, moderate 1, moderate 2, and strong (increasing the assumed gametocytemic potency of treatment). For ACT the simulated gametocytocidal components were mild, moderate, and strong. For ACT+PQ the parameterizations are moderate and strong; the numbers in brackets indicate the treatment day on which the PQ component was administered.

The quantity most relevant for control efforts is the total effect size, i.e., the reduction in transmission that includes the period of transmissibility before treatment. The longer that individuals wait to be treated, the less the maximum effect size achievable, because these individuals could transmit the parasite prior to treatment. After adding pretreatment infectivity (0.03 or 3.06 infectious days for JE and CG transformations respectively), the total mean fold-reductions for ACTs were 45.3 and 8.1, respectively. For ACT+PQ, total mean effect sizes were predicted to be 92.2 (JE) and 8.9 (CG), respectively. The reason for the large differences between these two transformations is how they incorporate pre-treatment infectivity. Thus, pretreatment infectivity plays a major role in the total effect size. For JE, gametocytes were assumed to be non-infectious early in the course of an infection, thus pretreatment infectivity was almost nonexistent and the effect size was determined by post-treatment infectivity. In contrast, the CG model assumed that gametocytes were infectious upon emergence, thus pretreatment infectivity was relatively large compared to post-treatment infectivity.

Purely schizonticidal treatments (with zero gametocytocidal activity) were predicted to reduce post-treatment transmission 6.2 to 5.7 fold (JE and CG, respectively). Including pretreatment infectivity, the mean effect size of a pure schizonticide was 6.2 and 3.9 (JE and CG, respectively). For CQ, which is moderately gametocytocidal against stage I–II gametocytes, total effect sizes were estimated to be 15.6 and 6.0 for the JE and CG parameterizations, respectively. These findings highlight the substantial benefit of drugs with more potent gametocytocidal activity in reducing transmission.

### Incorporating effects on mosquito-stage parasite development

The fold reductions in **Table 1** illustrate how antimalarials reduce transmission assuming 100% treatment coverage. However, these calculations do not incorporate the oocidal effects of some antimalarials. Treatments that are also oocidal (such as SP, LMF, and MFQ [42]) will have larger effect sizes than predicted in **Table 1** because of greater reductions in overall human-to-mosquito transmission. To calibrate oocidal drug effects, we compared unadjusted model-predicted infectivity and observed field infectivity post-treatment (see **Text S1** and **Figure S3**). For our ACT model infectivity, at day 7 the feeding studies indicated an infectiousness of approximately 2–3.5%, whereas the model predicted 5–8% under the JE parameterization (see **Figure S3C**). This small difference between model and field studies nearly disappeared by day 14 (possibly because of a dose-response effect of LMF on mosquito stage development). To incorporate oocidal activity, we thus assumed that ACTs (AL or AM) reduced onward infectivity by 50% compared to the mean values in **Table 1**. Mean post-treatment infectivity values became 0.35 (JE) and 0.47 (CG) net infectious days while the total effect sizes became 83.7 (JE) and 9.2 (CG). These data are shown in **Table 2**. Again, the assumed importance of pretreatment infectivity played a crucial role in determining the effect sizes of treatment when the oocidal effects of treatment were included.

**Table 2:** Modeled net infectivities and effect sizes of antimalarial treatment (adjusted for oocidal effects).

	ACT				ACT+PQ				
Parameterization	mild	mod	strong	mean	mod <0>	mod <2>	strong <0>	strong <2>	mean
Net Infectivity (JE)	0.45	0.33	0.27	**0.35**	0.24	0.26	0.10	0.16	**0.19**
Net Infectivity (CG)	0.60	0.45	0.37	**0.47**	0.34	0.41	0.16	0.29	**0.30**
Fold-reduction (post-treatment, JE)	70.5	96.2	115.9	**94.2**	129.7	120.4	318.5	194.6	**190.8**
Fold-reduction (post-treatment, CG)	49.0	65.6	79.6	**64.8**	86.3	71.3	180.1	99.7	**109.4**
Effect size, JE	66.8	89.3	106.0	**87.3**	117.4	109.7	253.1	168.1	**162.1**
Effect size, CG	8.9	9.2	9.4	**9.2**	9.5	9.3	10.0	9.7	**9.6**

**Abbreviations**: JE, Jeffery-Eyles; CG, Carter & Graves; ACT, artemisinin-based combination therapy; PQ, primaquine. Net infectivity values indicate the mean number of days that an individual is infectious for a mosquito vector, as explained in **Table 1**. Effect sizes illustrate the fold reductions in transmission for an ACT or ACT+PQ treatment, modeled with distinct levels of gametocytocidal activity). The numbers in brackets for the ACT+PQ treatments indicate the treatment day on which the PQ component was administered.

When examining the effects of ACTs plus PQ, our adjustment for the oocidal effects of PQ was different than that for LMF or MFQ, because PQ is active against mosquito stages for only a few days after treatment, but reduces infectivity almost completely during its period of activity [43], [44]. If we assumed that infectivity in the first three days post ACT+PQ treatment was zero, then the net infectivity post-treatment was 0.19 (JE) and 0.30 (CG) and the total mean effect sizes were 162.1 (JE) and 9.6 (CG), respectively.

**Figure 3B** illustrates the total effect sizes for each of the modeled drug parameterizations as a function of treatment coverage, including the oocidal effects of drugs from **Table 2**, assuming that the mean period of infectivity in untreated individuals is 30.5 days (mean untreated infectivity from **Table 1**) and assuming the JE density-to-infectivity parameterization for treated individuals. Each drug class is depicted in a different color with individual lines showing simulation outputs assuming varying levels of gametocytocidal activity (CQ: mild, moderate 1, moderate 2, strong); (ACT: mild, moderate, strong); (ACT+PQ: moderate 0 days delay, moderate 2 days delay, strong 0 days delay, strong 2 days delay). The black lines clustered within each drug class indicate the mean effect size across all simulations. The horizontal line illustrates a six-fold reduction in transmission, a threshold discussed below. The dotted vertical lines indicate the levels of treatment coverage needed to reach the six-fold reduction in total human-to-mosquito transmission for each drug class. The y-axis is in log-scale. This figure graphically illustrates the importance of treatment coverage in determining the effect size of a control program.

At 100% coverage (i.e. all infected individuals), the effect size of ACTs is 87.3 (JE parameterization, **Table 2**), assuming untreated infectivity is 30.5 days. However, this value drops quickly, yielding 16.3 at 95% coverage, 9.0 at 90% coverage, 4.8 at 80% coverage, and 3.2 at 70% coverage. For ACT+PQ, the effect sizes are 162.1, 17.7, 9.4, 4.9, and 3.3 at 100%, 95%, 90%, 80%, and 70% coverage, respectively. Treatment with ACTs exceeds a six-fold reduction threshold at ∼84.3% coverage of the total population, whereas the ACT+PQ regimen exceeds a six-fold reduction at ∼83.9% coverage of the total population.

### Sensitivity analyses

The above effect sizes (and those in **Figure 3**) were calculated assuming the untreated infectivity is 30.5 days (the mean of the JE and CG parameterizations) and using the JE parameterization for treated individuals. If we use the average of the JE and CG parameterizations for treated individuals, and still assume that the untreated net infectivity is 30.5 days, the pretreatment net infectivity is 1.55 (average from **Table 1**), the post-treatment net infectivity for ACTs is 0.41 (average from **Table 2**), and the computed effect sizes of ACTs become 15.6 at 100% coverage, 9.0 at 95% treatment coverage, 6.3 at 90% treatment coverage, 4.0 at 80% coverage, and 2.9 at 70% coverage. Assuming a post-treatment net infectivity of 0.245 for ACT+PQ (average from **Table 2**), these values become 17.0, 9.4, 6.5, 4.0, and 2.9 at the coverage levels listed above. Treatment with ACTs reaches a five-fold reduction in transmission at 85.5% coverage of the total population; with ACTs+PQ, the coverage level required is 85.0%. Treatment with ACTs exceeds a six-fold reduction threshold at 89.1% coverage, whereas the ACT+PQ regimen exceeds a six-fold reduction at 88.5% coverage. These outputs suggest a barely detectable impact of adding PQ to ACTs in the context of reducing transmission levels with these model assumptions.

### Mapping of fold-reductions in transmission necessary for malaria elimination

To contextualize the fold-reductions in transmission theoretically achievable with various treatments, we developed a set of maps of the fold-reductions in malaria transmission necessary to achieve elimination in low-transmission settings. These maps were derived from the worldwide maps of the basic reproductive number of malaria, *R*_0_ [45], assuming the malaria control coverage of 2010 as the baseline. We can also consider these maps as calculating the *R*_C_, i.e. the reproductive number under control efforts, as of 2010, though here we use the terms *R*_0_ and *R*_C_ interchangeably. *R*_0_ is a threshold criterion for transmission: if *R*_0_>1 over a given region, the disease will spread within this region (unless there is significant migration), if *R*_0_<1, the disease will disappear within this region (unless there is significant importation).

In brief, the *R*_0_ values described in [45] were developed by regressing various malariometric data (such as elevation and rainfall) on tens of thousands of parasite rate surveys and modeling the spatio-temporal autocorrelation structure of the residual variation. The regressions used were Bayesian and geostatistical, producing a full Bayesian posterior distribution for the age-standardized parasite rate at each pixel (with a per-pixel size of 5×5 km, i.e. 5 km^2^).

These worldwide maps of predicted parasite rates varied in intensity from pixel to pixel, given different magnitudes of various malaria covariates. By utilizing empirical and theoretical relationships, the maps were combined with aspects of malaria that remain constant over time and space to calculate *R*_0_. One such malaria invariant is the net infectivity of infected humans to mosquitoes, assuming no acquired immunity developed over the course of repeated infections [23]. To calculate this invariant, we used our within-host model of malaria transmission to simulate the progression of infectivity in thousands of simulated individuals, and then calculated the mean area under the human-to-mosquito infectivity curves [23].

To achieve elimination in a given area, the fold-reduction in transmission under control must be greater than or equal to the *R*_0_ [46]. Thus, we took the worldwide maps of *R*_0_ in [45] and calculated the fold-reductions necessary over each pixel to reduce the estimated *R*_C_ to below 1. Our maps of the transmission reductions include estimates of uncertainty inherited from the *R*_C_ posterior densities at each pixel. **Table 3** summarizes these maps by providing the number of people living in areas requiring <2 fold reductions in transmission to interrupt transmission, as well as 2–5, 5–10, 10–20, 20–50, 50–100, and >100 fold reductions to interrupt transmission. **Table 3** calculates these necessary fold-reductions with 75% confidence, i.e., our posterior estimates of *R*_C_ fall within the given regions with 75% confidence.

**Table 3:** Required effect size required to reduce *R*_C_ below 1, worldwide and solely in Southeast Asia, stratified by populations at risk.

	Required effect size	Population	Population at risk (%)	Total population (%)	
**Worldwide**	no risk	4,362,130,000		62.89%	
	<2	1,672,204,000	64.97%	24.11%	
	2–5	223,901,000	8.70%	3.23%	
	5–10	94,173,000	3.66%	1.36%	
	10–20	83,986,800	3.26%	1.21%	
	20–50	123,546,000	4.80%	1.78%	
	50–100	84,695,100	3.29%	1.22%	
	>100	291,346,000	11.32%	4.20%	
**Total population**					6,935,981,900
**Total pop at risk**					2,573,851,900
**Southeast Asia**	no risk	56,595,000		24.98%	
	<2	135,508,000	79.71%	59.80%	
	2–5	19,389,600	11.41%	8.56%	
	5–10	7,800,520	4.59%	3.44%	
	10–20	3,838,410	2.26%	1.69%	
	20–50	2,955,060	1.74%	1.30%	
	50–100	477,035	0.28%	0.21%	
	>100	39,566	0.02%	0.02%	
**Total population**					226,603,191
**Total pop at risk**					170,008,191

**Abbreviations:** Southeast Asia is defined here as encompassing the countries Thailand, Myanmar, Laos, Vietnam, and Cambodia. Required effect size denotes the fold-reduction in malaria transmission needed to interrupt transmission over the given population. Confidence of interruption is at the 75% level.

Because our within-host model simulates the progression of infections for individuals with no prior history of malaria infection, our modeling conclusions are most relevant for areas of low transmission where individuals have accumulated little acquired immunity from prior infections. We thus restricted our analyses to areas with *R*_0_<10, which excludes the more endemic areas of Africa and aligns with the transmission levels prevalent in Southeast Asia. In terms of biting intensity, an *R*_0_ of 10 translates to a yearly entomological inoculation rate of approximately 3 infectious bites per person per year, which would result in approximately 1.5 infections every year [47]. This upper limit of analysis can be compared with the intensity reported in an area of ‘low and seasonal’ transmission in Thailand, where individuals had one infection every other year [34].

**Figure 4:**
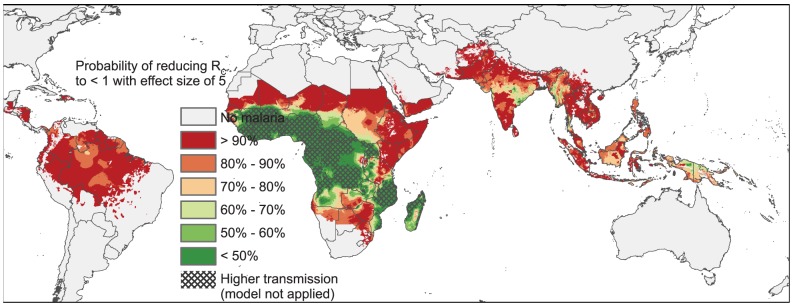
Worldwide map of the predicted probabilities that a five-fold effect size will interrupt malaria transmission. This map shows the predicted probabilities that a five-fold reduction in transmission (‘five-fold effect size’) would interrupt malaria transmission over a given pixel. Map pixel size is 5 km^2^. In order to interrupt malaria transmission in a given area, the basic reproductive number for malaria under control (*R*_C_) needs to be reduced below 1. Probabilities for each pixel are calculated according to Bayesian posterior estimates of uncertainty [45]. Probabilities have been binned into six categories for clarity. Areas with high transmission (defined as at last a 50% probability of *R*_0_>10) are masked because our model results are applicable to regions of relatively lower transmission. Most of Sub-Saharan Africa is masked because of the very intense transmission. However, most of Sahelian Africa, as well as East Africa, parts of Southern Africa, most of India, as well as most of Southeast Asia and essentially all of South America have high probabilities of interruption at this control level. Note that local conditions (within a given pixel) may be more or less favorable to transmission than the per-pixel averages shown here, and so these maps are most applicable for regional or country-level planning, rather than local-level control efforts. Microenvironments or ‘hotspots’ might require additional interventions and/or greater treatment coverage than the per-pixel average [67]. Data collection sites used to construct the maps are reported in [45]. A partial database of the actual site locations and the measured levels of malaria endemicity can be found on the Malaria Atlas Project (MAP) website: www.map.ox.ac.uk.

**Figure 5:**
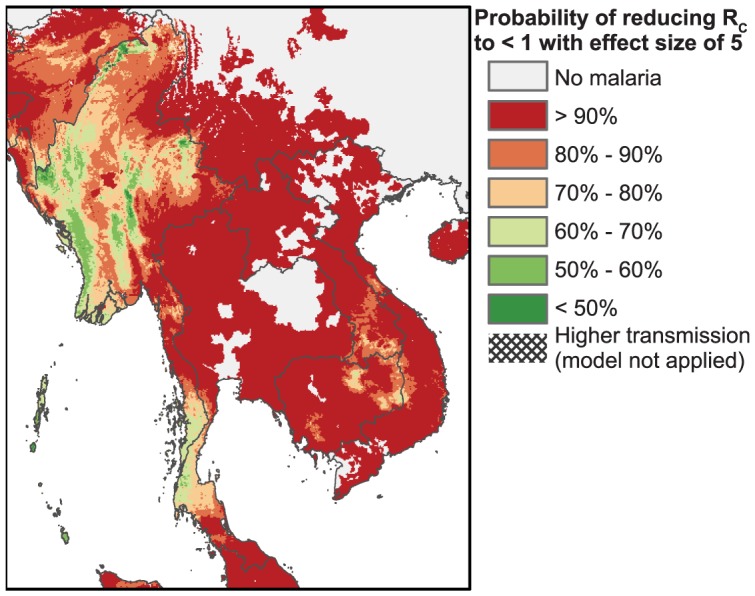
Map of the predicted probabilities that five-fold reductions will interrupt transmission in Southeast Asia. The predicted probabilities that a control effort with a five-fold reduction would interrupt transmission are shown for Southeast Asia, using the same masking of high transmission areas (*R*_0_>10) and mapping assumptions as for **Figure 4**. Areas that appear to be uniform may have small-scale heterogeneities in transmission that are beyond the scale of this map. Map pixel size is 5 km^2^.

**Figure 4** provides a worldwide map of the probabilities that areas can interrupt malaria transmission (*R*_C_<1) assuming a five-fold reduction in transmission. Areas with *R*_0_>10 are masked, as these regions have such high transmission that our modeling predictions are less relevant. Fold reductions were organized into 6 bins for clarity. **Figure 5** shows these probabilities of interruption for Southeast Asia, where transmission is generally much lower than in Africa. **Figures S4** and **S5** present maps of the probabilities of interrupting transmission assuming two- or ten-fold reductions in transmission, respectively. These maps are discussed below in the context of the reductions achievable with antimalarial drugs.

## Discussion

This study calculates the effects of antimalarial therapies on *P. falciparum* transmission, using a within-host model of malaria infection [23] and a PK/PD model parameterized from field studies. The effects of drugs are modeled on both asexual and sexual stages of parasite development under different assumptions of gametocyte-to-infectivity relationship. We also generate global maps of the fold reductions in malaria transmission, i.e. the effect sizes, necessary to achieve elimination in regions of low endemicity (defined as having local *R*_0_ values less than 10).

From our model outputs, we can generate three major conclusions. First, the infectivity of individuals before treatment plays a crucial role in determining effect size. If treatment is delayed more than only a few days after the onset of fever, and gametocytes are infectious during this period, then the effect sizes achievable even with first-line ACT therapies plus the gametocytocidal agent PQ are limited. Second, if we account for the effects that the partner drugs LMF and MFQ exert upon mosquito stages of the parasite life-cycle, then there is little difference in the benefits of ACTs versus ACTs+PQ in terms of transmission reductions. Both regimens are extremely effective at stopping onward transmission, with many fold greater benefits versus purely schizonticidal treatments that act only upon asexual blood stage parasites. Third, the proportion of individuals receiving treatment has a major impact on reductions in transmission (**Figure 3B**). In **Tables 1** and **2** our effect size calculations assumed 100% coverage. Because untreated individuals are so much more infectious than treated individuals, leaving even a few individuals untreated drastically reduces the effectiveness of a control program.

We can put these fold-reductions in context using our maps of transmission reductions necessary for elimination. **Figure 4** illustrates a worldwide map of the probabilities that a five-fold reduction in transmission would interrupt the spread of malaria. In this map the pixel size is 5 km^2^. Because our model is most applicable in regions of relatively low transmission, we masked out the regions where *R*_0_, the basic reproductive number, is predicted to be greater than 10 (with a probability exceeding 50%). Higher transmission regions are more difficult to model, given the complex interactions of immunity, superinfection, and control. As can be seen from the map, many areas of Africa have such intense transmission that *R*_0_ exceeds 10, and we cannot say how transmission might be affected by the use of drugs in such areas.

However, examining the map, one can visualize many regions of Africa, including the Sahel, most of East Africa, and parts of Southern Africa, where elimination would appear possible with a five-fold reduction in transmission. Further, much of India and Southeast Asia have low enough transmission that elimination would be possible at this level of control. Prospects for elimination in Myanmar and southern Thailand, however, do not appear to be favorable. **Figure 5** provides a zoomed-in view of the probabilities of malaria elimination with an effect size of five, focusing on Southeast Asia. **Table 3** provides a quantification of the populations at risk both worldwide and in Southeast Asia, as well as the populations where elimination is possible at different levels of control. Worldwide, regions where malaria can be interrupted with five-fold reductions contain 74% of the population at risk; in Southeast Asia regions that can interrupt transmission with five-fold reductions harbor 91% of the population at risk.

Given these maps and quantifications of populations at risk, we can apply our modeling results to determine the percentage of the population that needs to be treated promptly with antimalarials to interrupt transmission in various areas. **Figure 3B** illustrates the relationship between treatment coverage with different antimalarials and the resulting fold reductions in transmission, assuming the Jeffery-Eyles gametocyte density-to-infectivity relationship (used to calculate the needed treatment coverage levels below). To achieve a five-fold reduction in transmission, approximately 81% of the total infected population would need to be treated with ACTs or ACTs+PQ. As a comparison, achieving this fold reduction with CQ (with weak activity against early-stage gametocytes) or a schizonticide (with no gametocytocidal activity) would require treatment coverage of ∼86% and ∼96% respectively. To achieve a six-fold reduction in transmission, approximately 84% of the total infected population would need to be treated with ACTs or ACTs+PQ (illustrated in **Figure 3B**). Our modeling thus suggests that the addition of PQ to an ACT would provide almost negligible benefits at these levels of coverage, reducing the fraction of the population needing to be treated by less than 1% versus treatment with ACTs alone that already provide quite potent gametocytocidal activity (**Figure 3B**).

Combining the maps and the within-host modeling results based on our JE parameterization, we thus estimate that promptly treating ∼81% of the total infected population with ACTs and/or ACTs+PQ would interrupt transmission in areas covering 91% of the population in Southeast Asia. These coverage rates are for the infected population as a whole, regardless of whether individuals are symptomatic or not. In a study conducted in a region of western Thailand with low and seasonal transmission, most infections (87%) were found to be symptomatic [48]. In experimental challenge studies among human volunteers, all subjects displayed some degree of symptoms [49].

If we take the former proportion (87%) as the percentage of the infected population that is symptomatic, then 93% of the symptomatic population would need to be treated with ACTs and/or ACTs+PQ to achieve interruption in the areas of **Figure 5** based on the predicted five-fold reduction; the percentage rises to 97% to achieve six-fold reductions. Thus, it is possible that treating only symptomatic individuals may be sufficient to eliminate transmission throughout most of Southeast Asia. Almost all of these individuals, however, would need to be reached with treatment (either through a public campaign or private sector provisioning or a combination of both) in order to interrupt transmission, using drugs alone. Our result that prospects for malaria elimination are favorable for most of Southeast Asia is supported by two other studies that also find that elimination efforts are feasible using antimalarials in this region [50], [51]. **Figures S4** and **S5** illustrate the probabilities assuming control interventions with two-fold and ten-fold reductions, respectively. At the two-fold level, much of central Thailand can interrupt transmission, but there are significant portions of Myanmar, Cambodia, Southern Laos, and Southern Thailand where elimination is not likely. At the ten-fold level, there are small pockets in Southern Thailand as well as large areas in Myanmar where interruption is still not likely.

These estimates may be somewhat optimistic because we are using only the JE density-to-infectivity relationship when calculating the infectivity of treated individuals. As we are focusing on low transmission areas, this assumption seems reasonable (the JE relationship was derived from individuals with no prior infections). However, if we take the mean of the JE and CG relationships for treated individuals, then we predict that it would require treating 85.5% of the total population, or 98.3% of symptomatic patients with ACTs to achieve a five-fold reduction (85.0% of the total or 97.7% of symptomatic patients with ACTs+PQ). Further, we find that it is not possible to achieve a six-fold reduction only treating symptomatic individuals with either the ACTs or the ACTs+PQ modeled here, assuming the mean of the JE and CG relationships for treated individuals. Thus, the assumed density-to-infectivity relationship has a large effect on the calculated effectiveness of control programs.

We note that our maps predict the levels of control necessary to interrupt transmission at the per-pixel level (5 km^2^), incorporating uncertainty analysis. These average reductions needed to interrupt malaria transmission are not at the per-village or per-household level. Hotspots of transmission will need to be identified and treated in order to achieve elimination in a given region. The uncertainty for each pixel takes into account this heterogeneity to some degree, but nevertheless caution is advised before using these maps for local-scale planning. We would suggest our maps be used to guide elimination planning at a regional or national level; for elimination planning at a district or city level more intensive surveillance will likely be needed.

If we consider the timelines to elimination, the narrower the margin by which the effect size exceeds the threshold for elimination, the longer elimination will take, as population-wide transmission will decay more slowly [46]. Conversely, the higher the proportion of individuals above the needed threshold, the faster elimination will be achieved [46]. We do not compute the quantitative benefits of mass drug administration here, and instead focus on individual-level treatment. However, we would qualitatively expect that mass drug administration may provide a benefit to elimination efforts by speeding an area toward faster elimination, assuming that the critical level of coverage can be reached (i.e. the *R*_C_ of the region drops to below 1). Once an area has eliminated malaria, the costs of maintaining elimination may be less than those needed to achieve elimination in the first place, though more research is needed on strategies to maintain elimination in previously endemic areas [7].

In areas where antimalarials are predicted to be insufficient to achieve elimination, other interventions may be included in control efforts to increase the effect size of the combined control effort. The combined effect size is simply the product of both component interventions. For example, if the coverage level with antimalarials reduces transmission by three-fold and distribution of bed nets reduces transmission another three-fold, the combined effects are a nine-fold reduction in transmission (as long as there are no antagonistic interactions between the two efforts). Thus, high fold-reductions can be achieved by bundling interventions. While we do not compute the effect sizes of other interventions here, the results in this paper can be combined with other modeling efforts for the purposes of an integrated elimination effort.

Given these conclusions, serious efforts to eliminate malaria will require extensive planning and sustained support [6]. We note the encouraging prediction that high coverage (at least 81–85% of total infections, corresponding to an estimated 93–98% of symptomatic infections) with ACTs that act against *P. falciparum* asexual, sexual, and mosquito stages might suffice to interrupt transmission throughout most of Southeast Asia, especially if complimented by insecticide-treated bed net distribution to reduce population infectivity. We also note that the addition of a single dose of a purely gametocytocidal drug such as PQ to ACTs can reduce onward transmission slightly. However, the focus of control efforts should be on maintaining a high level of treatment coverage. Based on our modeling, PQ and similarly gametocytocidal therapies added to ACTs do not appear to be a magic bullet ensuring elimination and add only nominally to the transmission reductions achievable with ACTs that act against the various parasite stages at feasible levels of coverage.

Efforts are ongoing to utilize our model to predict the effects of possible emerging artemisinin resistance, which threatens existing ACT control strategies [10], [52], [53]. Additional modeling is also required to delineate better what measures beyond expanded ACT and insecticide-treated bed net coverage would help reduce the *R*_C_ in Africa to levels that might make elimination an achievable goal.

## Materials and Methods

### Model description

Our recently reported within-host mathematical model [23] was utilized to simulate asexual and sexual blood stage parasite densities over time in untreated and treated individuals. This model reproduces the range of observed parasite densities among individuals undergoing malaria therapy, wherein adult males with tertiary syphilis (and no acquired immunity to malaria) were infected with various strains of *P. falciparum* to induce a fever in order to clear the syphilis [29], [54]–[56]. Our model uses a combination of parasite antigenic variation and host immune responses to reproduce the observed range of responses in these patients. The model calculates the density of asexual parasites every two days and uses log-linear interpolation to generate daily counts. The model also calculates the daily human-to-mosquito infectivity using gametocyte density-to-infectivity relationships derived from mosquito feeding studies on human volunteers [23]. The source code for our model is provided in **Dataset S1** (see **SI**). We note that our modeling uses discrete-time difference equations rather than a continuous time model, to calculate both asexual densities and gametocyte densities over time. We chose the former, as the calculation of gametocyte densities from asexual densities is difficult with a continuous-time model because gametocyte densities are a function of weighted cumulative asexual densities and are highly stochastic. For a thorough description, we refer to [57].

We also note that an insightful report by Kay and Hastings [58], building on earlier work from this group [59], simulated the concentrations of both artemisinin derivatives and ACT partner drugs using compartmental PK/PD modeling. These authors also simulated the killing effects of these drugs against asexual parasites assuming Michaelis-Menten dose-response functions (which are similar to the Hill functions used here). These authors focused on the effects of emerging artemisinin resistance and provided substantial data on artemisinin PK/PD properties. Our two studies differ in that we simulated parasite densities daily and so we did not explicitly model artemisinin PK values because of the short half-lives of the artemisinins. Further, our stochastic difference equation model of within-host parasite growth and immune response was calibrated to match the range of variation in the malaria therapy data [23]. We also modeled the effects of drugs against asexual, sexual, and mosquito stages of development. Thus, The Kay and Hastings study [58] is a representation of the effects of drugs against asexual stages incorporating resistance, whereas our study focused on the effects of drugs on total human malarial transmission. Importantly, their simulations predict that the spread of ART resistance would result in a potentially rapid decline in ACT effectiveness. In future studies, we plan to investigate the effect of ART resistance on effect sizes and how this would impact the required treatment coverage to drive the *R*_C_ to below 1 in low-transmission settings.

### Antimalarial drug pharmacokinetics

PK modeling was used to simulate the concentrations of antimalarial drugs after uptake. The concentrations of CQ and its active metabolite, monodesethyl-chloroquine (mdCQ), were simulated using a non-compartmental model parameterized with data from Papua New Guinean children [60]. For LMF, a two-compartmental model was developed from plasma concentration data from the treatment of uncomplicated individuals in western Thailand (Mae La) [61]. For MFQ, a non-compartmental model was developed that incorporated data from two studies, one in Thailand [62] and the other in Peru [63] (the Peruvian study used whole blood concentrations, rather than plasma). The plasma concentrations of the artemisinins were not modeled, although such data exist [62]. This is because the half-lives of the artemisinins are so short (∼1–3 hr) that effective concentrations are gone within one day after uptake [62]. A full description of the PK modeling is provided in the **SI**.

### Drug pharmacodynamics against asexual parasites

The dose-response effect of antimalarials against asexual parasites was assumed to follow the commonly used ‘Hill function’ [64], a four-parameter dose-response function: 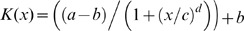, where *a* is set to 0, *b* is set to 1, *c* is what we term the EC_50_, *d* is the Hill slope, and *x* is the plasma concentration of the drug. The Hill function dose-response curve type was chosen because this function type was utilized by both of the references that provided *in vivo* EC_50_ values for MFQ [65], [66], and because many of the *in vitro* studies using CQ and LMF used Hill dose-response relationships to model the effects of drugs against asexual blood stage parasites. Because each drug has a characteristic maximum inhibitory effect, this dose-response function was scaled by the maximum parasite reduction ratio (PRR) for each drug. To determine the effect on asexual parasites of a drug concentration on a given day *t* of modeling, the asexual parasite densities from day *t*-1 were used as inputs into the within-host model. The predicted densities on day *t* were then calculated, incorporating the effects of host immunity and parasite growth. The mean of 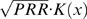 over day *t* was then subtracted from the within-host simulations after appropriate log transformation to calculate the end of day asexual parasite densities, incorporating the effects of host immune responses, parasite growth, and drug concentrations. These densities were then used to calculate the asexual parasite densities on day *t*+1, and so on until the end of the simulation time. A full description of the PD modeling against asexual blood stages is provided in the **SI**.

### Drug pharmacodynamics against gametocytes

The dose-response effect of antimalarials against gametocytes was assumed to follow a binary model, where antimalarials act against gametocytes only if drug concentrations are above a certain drug-specific threshold. This binary model was adopted because of the current paucity of dose-response data against gametocytes, as compared to asexual blood stage parasites where the many data sets permit the use of Hill slopes to define dose-response relationships. The threshold for gametocyte activity was chosen to be the *in vitro* IC_50_ against asexual blood stage parasites scaled by a factor of five [21]. For CQ, LMF, and MFQ, this value is 40, 174, and 322 ng/ml, respectively (**SI**). To determine the stage-specific gametocytocidal effects of drugs, the within-host malaria model was first run assuming treatment with a purely schizonticidal combination therapy. The post-treatment gametocyte clearance curves from these simulations were then compared to clearance curves from field studies using SP [28], [30]–[38] to validate the model outputs. In separate simulations, we assumed that individuals were treated with a combination that was weakly gametocytocidal, and these results were compared to field data from CQ trials [28], [30]–[33] to choose a drug parameterization. We also performed simulations assuming treatment with a stronger gametocytocidal combination, and modeled our parameters by comparing these results with ACT field trial data [28], [30]–[32], [34]–[36], [38]. Finally, we simulated treatment with a stronger gametocytocidal combination paired with a third highly gametocytocidal drug, and compared these results to ACT+PQ field data [35], [36], [38] to parameterize this combination. Outputs from modeled drug parameterizations that were consistent with observed trends were considered representative of that type of treatment. A full description of the process of model parameterization of antimalarial effects against gametocytes is provided in the **SI**.

### Drug pharmacodynamics against mosquito-stage parasites

Once the ensembles of gametocyte densities after treatment had been generated for various drug combinations, we used two different gametocyte density-to-infectivity relationships (‘Jeffery-Eyles’ and ‘Carter & Graves’) to translate the daily gametocyte densities into predicted human-to-mosquito infectivities [23]. These modeled daily infectivities were then compared to field studies in which mosquitoes were fed on human volunteers after treatment. For the effects of a single dose of PQ on drug transmission, we assumed that the first three days post-treatment were non-infectious (including the day of treatment). For the effects of partner drugs with longer half-lives that are active against sexual-stage parasites for longer periods (LMF, MFQ), we scaled the area under the infectivity curve (AUIC).

### Analysis of model uncertainty

To quantify the uncertainty associated with our predictions, we utilized an ensemble modeling approach [40], [41]. In ensemble modeling, various scenarios are simulated to illustrate the effects of changing assumptions of model outputs. Ensemble modeling is especially appropriate when insufficient or conflicting data exist to determine the relative likelihoods of the possible scenarios. For our ensembles, we used different sets of assumptions about the stage-specific effects of drugs against gametocytes and the type of gametocyte density-to-infectivity relationship to map out the uncertainties associated with our best-estimate predictions of the effects of drugs on transmission.

## Supporting Information

Dataset S1 **Text of source code for model (*final_model.rtf*).**
(RTF)

Figure S1 **Post-treatment gametocyte prevalences from field studies.** The graphs show gametocyte prevalences of field populations after antimalarial treatment. Gametocyte positivity was assessed using microscopy (threshold ∼5–10 gametocytes per µL blood). The notation (‘− Day 0’) indicates that only individuals who were gametocyte negative at admission were included in the study. Field notes include the location of study and subset of population treated. All study curves represent mean population values linearly interpolated from measured prevalences. (**A**) The percentage of individuals positive for gametocytes after treatment with sulfadoxine-pyrimethamine (SP) [28], [30], [33]. The pattern after SP treatment can be described as an inverted-V: few mature gametocytes were present at treatment because treatment was relatively prompt and some studies excluded gametocyte carriers at admission. A peak in prevalence was caused by sequestered gametocytes emerging into the blood stream. The peak gradually declined as the immune system cleared gametocytes from the blood. (**B**) Gametocyte prevalences after treatment with chloroquine (CQ) or amodiaquine (AQ) (sometimes in combination with SP) [28], [30]–[33]. (**C**) Gametocyte prevalences after treatment with various artemisinin-based combination therapies (ACTs): (artemether-lumefantrine, AL), (artesunate, A1, A3, AS), (artesunate-mefloquine, AM), (dihydroartemisinin-piperaquine, DHP) [28], [30]–[32], [34]–[36], [38]. (**D**) Gametocyte prevalences after treatment with ACTs plus primaquine (PQ) [35], [36], [38].
(PDF)

Figure S2 **Comparison of modeled post-treatment gametocyte prevalences to field study data.** The post-treatment gametocyte prevalences from **Figure S1** were averaged to create a set of target data to parameterize the modeled effects of antimalarials on transmission. (**A**) The mean of the field data after treatment with sulfadoxine-pyrimethamine (SP) [28], [30], [33] is illustrated by the red line and the range of observed responses are depicted in light blue. The modeled gametocyte carriage curves are shown in black and green. All model treatment was assumed to start 5 days after the first onset of fever, consistent with average behavior from field studies [34], [38]. All model outputs represent the mean of 1,000 runs. The solid black line illustrates modeled gametocyte carriage among untreated individuals; the dashed black line illustrates modeled clearance in untreated individuals among gametocyte negatives at admission (‘- Day 0’). Modeled gametocyte clearance in untreated individuals is mediated only by immune processes as described in [23]. The solid green line depicts modeled gametocyte prevalence after treatment with a schizonticidal combination therapy (i.e. a short-lived component that rapidly kills asexual parasites and a longer-lived one that is less potent; neither are assumed to affect gametocytes); the dashed green line depicts modeled gametocyte carriage after schizonticidal treatment, including only gametocyte negative individuals at treatment (‘- Day 0’). (**B**) Gametocyte prevalences after treatment with chloroquine (CQ) or amodiaquine (AQ) (sometimes in combination with SP) [28], [30]–[33]; mean values are illustrated in red, range in blue. Modeled gametocyte prevalences are also provided. Model prevalences assume treatment with a combination of drugs (short-lived and long-lived) that kill asexual parasites but only affect early stage gametocytes. Mild, moderate (2 being stronger than 1), and strong model outputs vary in the assumed intensity of early stage gametocyte killing. (**C**) Gametocyte prevalences after treatment with various artemisinin-based combination therapies (ACTs) [28], [30]–[32], [34]–[36], [38]; mean values are in red and the range is illustrated in blue. Model data illustrate treatment with a combination of drugs, a shorter-lived one that kills both early and late stage gametocytes and a longer-lived partner that only kills early stages. Model outputs vary in their assumed gametocyte killing strengths. The curve labeled ‘no late stage effects’ illustrates the effects of a combination that has no effect on gametocytes aged >13 days old. (**D**) The same data as in (**C**) are illustrated with the exception that two ACT field studies were removed from the field data curves because of potentially confounding drug resistance effects (CQ+AS, [31] and SP+AS, [35]). (**E**) Gametocyte prevalences after treatment with ACTs plus primaquine (PQ) [35], [36], [38]; the red line indicates the mean of field studies; the range of studies is shown in blue. Model outputs assume treatment with three drugs: a short-lived drug that kills early and late stage gametocytes, a long-lived partner that kills late stage gametocytes only (these first two drugs were parameterized from the ACT field data, using the ‘mild’ parameterization), and a second short-lived partner drug that strongly kills both early and late stage gametocytes. The designation of <day 0> or <day 2> refers to the numbers of days that single dose PQ was delayed after initiating ACT treatment (i.e. simultaneously or 2 days after).
(PDF)

Figure S3 **Infectivity to mosquitoes after treatment.** These graphs illustrate the probability that a human will infect a mosquito following antimalarial treatment. Infectivity is defined as the probability that a mosquito bite will produce oocysts. Field study data are indicated with markers; model outputs are indicated by curves. All model outputs represent the mean of 1,000 runs; treatment was assumed to begin 5 days after first fever. Field markers represent the mean from a set of mosquito feedings. Model output curve coloring is taken from **Figure S1**. Two different gametocyte density-to-infectivity relationships were used to model infectivity: Jeffery-Eyles (in bold; JE) and Carter & Graves (CG) [23]. Some field and model data included only gametocyte negative individuals at admission, as indicated by (‘Day 0 −’); others included all individuals (‘Day 0 +/−’). (**A**) Field data post-treatment with SP (sulfadoxine-pyrimethamine) or SP plus amodiaquine (AQ) [28]. The modeled outputs are from simulations approximating the effects of chloroquine (CQ) treatment. Field-measured infectivity after SP treatment resembles that of modeled CQ treatment, even though gametocyte densities after SP treatment were much higher than after CQ. The discrepancy is explained in part by evidence that SP acts against the mosquito stages of development, thus reducing the human-to-mosquito infectivity for given levels of gametocytemia [68]–[70]. (**B**) Field-measured infectivity after treatment with CQ, SP, or CQ+SP [30]–[33]. Model outputs were normalized to remove simulated individuals positive at treatment. The JE parameterization is more consistent with field data, although it is unclear how infectious individuals were 0–3 days post-treatment. (**C**) Field-measured infectivity [28], [37] and modeled outputs after treatment with artemisinin-based combination therapies (ACTs), including only individuals that were gametocyte negative at admission. (**D**) Field-measured infectivity [30]–[32] and modeled outputs after treatment with artemisinin-based combination therapies (ACTs), including all treated patients. The JE parameterization is more consistent with field data, at least after the initial period of infection. (**E**) Field-measured infectivity after treatment with ACTs [28], [37]; modeled infectivity is for ACTs plus primaquine (ACT+PQ). The lack of field infectivity data for the ACT+PQ combination treatment precludes direct comparison, but the ACT-treated field data is provided for reference. (**F**) Field-measured infectivity after treatment with ACTs, excluding individuals positive for gametocytes [30]–[32]; modeled infectivity is for ACT+PQ, also after exclusion. The JE parameterization produced infections that are infectious only at an extremely low level.
(PDF)

Figure S4 **Maps of the predicted probabilities that a two-fold effect size will interrupt malaria transmission.** The upper map shows the predicted probabilities that a two-fold reduction in transmission (‘two-fold effect size’) would interrupt malaria transmission over a given pixel. Map pixel size is 5 km^2^. In order to interrupt malaria transmission in a given area, the basic reproductive number for malaria under control (*R*_C_) needs to be reduced below 1. Probabilities for each pixel are calculated according to Bayesian posterior estimates of uncertainty (45). Probabilities have been binned into six categories for clarity. Areas with high transmission (*R*_0_>10) are masked because our model results are applicable to regions of relatively lower transmission. Note that local conditions (within a given pixel) may be more or less favorable to transmission than the per-pixel averages shown here, and so these maps are most applicable for regional or country-level planning, rather than local-level control efforts. Microenvironments or ‘hotspots’ might require additional interventions and/or greater treatment coverage than the pixel average [67]. The lower map inset illustrates the predicted probabilities that a control effort with a two-fold reduction would interrupt transmission in Southeast Asia, using the same masking of high transmission areas (*R*_0_>10) and mapping assumptions as for the upper map. Areas that appear to be uniform may have small-scale heterogeneities in transmission that are beyond the scale of this map.
(PDF)

Figure S5 **Maps of the predicted probabilities that a ten-fold effect size will interrupt malaria transmission.** The upper and lower maps are illustrated as per **Figure S4**, except that **Figure S5** shows the predicted probabilities that a ten-fold reduction in transmission (‘ten-fold effect size’) would interrupt malaria transmission over a given pixel (size is 5 km^2^).
(PDF)

Text S1 **Pharmacokinetic and pharmacodynamic equations and distributions ****[71]**–**[90]****.**
(PDF)
