# Supplementary material for: Modeling Within-Host Effects of Drugs on Plasmodium falciparum Transmission and Prospects for Malaria Elimination
Source: PLoS Comput Biol. 2014 Jan 23;10(1):e1003434. doi: 10.1371/journal.pcbi.1003434 (PMC3900379; doi:10.1371/journal.pcbi.1003434)
Supplement: Dataset S1 — Text of source code for model (final_model.rtf). (RTF) [file pcbi.1003434.s001.rtf]

%final_model.m%HOTFOOT instantiation function final_model(lambda) %% Prepare the Computing Space     %clear all    %clc    format short g    format compact %% Begin the clock     tic; %% Set and store the random number seed for the random number stream     %seed = randi(2^32);                                %not supported for multiple clones    newtime=clock; seed = (newtime(6)*10^4);    stream = RandStream('mt19937ar','Seed',seed);       %use to view default: RandStream.getDefaultStream    RandStream.setDefaultStream(stream);    defaultStream = RandStream.getDefaultStream;        %use reset(defaultStream) to reset the defaultStream value    get(defaultStream)     %seed = randi(2^32);                                %not supported for multiple clones    %newtime=clock; seed = (newtime(6)*10^4);    %rng(seed);    %rng %% Major adjustable parameters     numofruns = 10;                         %Global constant     RRfstdrug = log10(1/10000); %-4;        %Maximum reduction ratio of first drug (default value 10^4)                      %RRsnddrug                              %Defined below    treatmentwait = 0;                      %This determines DHA gam effect and wait for treatment    probtreat = 1;                          %Probability that the infection will be treated    drug = 'MEF';                           %Determines the partner drug used: CQ, MEF, LMF    MEF_type = 'loose';                     %Determines the type of MEF dosage    infect_fn = 'JefferyEyles';             %Determines relationship between gametocytemia and infectivity    pretreated = 0;                         %If pretreated = 1, then there is residual partner drug regardless of treatment                                       rand_PK = 1;                            %If rand_PK = 1; the pharmacokinetics of the partner drug will be stochastic    trans_block = 0;                        %If trans_block = 1, then kill all gametocytes after consumption    shift = 0;                              %Number of days partner drug is shifted    random_pretreat = 0;                    %If random_pretreat = 1, then the pretreatment drug levels will be randomized     Adjalley = 1;                           %Assignment of gametocytocidal assumptions; LMF & MEF = 1 or 2(w/delay)        treatmentoverride = [0 23];             %If treatmentoverride(1) == 1, feverday will be ignored and treatment will start                                                %on day treatmentoverride(2)         stochastic_start = 0;                   %Determines whether treatment start is stochastic based on oldfeverday        params = [8.214976698893794   3.641801143963211];   % See line 770; Weibull distribution        %% Important Constants (Default)     %    DHA_invitro_fact = 1;                   %This factor converts in vitro GAM killing to in vivo phenotype (DEPRECIATED)    prtnr_invitro_fact = 1;                 %This factor converts in vitro GAM killing to in vivo phenotype (DEPRECIATED)    snd_stage_cst = 1;    fst_stage_cst = .55;                    %modifies DHA killing effect    %        gam_effect = 1;                         %Determine whether first drug has an effect on gametocytemia    medfevereffect = 1;                     %Effects of fever on transmission    notrans = 2;                            %Gametocytemia observed first two days after gametocyte patency are nontransmissible    JEwaittime = 17;                        %Maximum time to emergence of infective gametocytes for all cases     minasexualval = 10^-15;                 %Zero on log scale     mingamval = 10^-15;                     %Zero on log scale  %% Apply DHA treatment/no treatment     COMB_effect_daily                   = zeros(1,40);    full_DHA_day                        = 3; % Determines gametocytemic effect of DHA-equivalent    COMB_effect_daily(1:full_DHA_day)   = 1;     treatmentcourse_fstdg               = 3; % Determines asexuals effect of DHA-equivalent        %COMB_effect_daily                   = zeros(1,40);    %full_DHA_day                        = 0; % Determines gametocytemic effect of DHA-equivalent    %COMB_effect_daily(1:full_DHA_day)   = 1;     %treatmentcourse_fstdg               = 0; % Determines asexuals effect of DHA-equivalent    %% Additional constant     full_time = 0;   % This value determines the full activity of the partner drug against asexuals  %% GAM Killing Constants (Adjalley et al.)     GAM = zeros(12,4);     GAM(1,:) = [0.776 1 1 1];                                   % LMF    GAM(2,:) = [0.776 0.791 0.838 0.889];                       % LMF (delayed action)    GAM(3,:) = [0.874 1 1 1];                                   % ATQ    GAM(4,:) = [0.874 0.792 0.685 0.704];                       % ATQ (delayed action)    GAM(5,:) = [0.823 1 1 1];                                   % TFQ    GAM(6,:) = [0.823 0.457 0.819 0.713];                       % TFQ (delayed action)    GAM(7,:) = [0.697 0.895 1 1];                               % PPQ    GAM(8,:) = [0.767 0.821 0.884 0.841];                       % PMQ    GAM(9,:) = [0.62 0.858 1 1];                                % PND    GAM(10,:) = [0.526 0.775 1 1];                              % mdAQ    GAM(11,:) = [0.547 0.826 0.812 0.831];                      % DHA    GAM(12,:) = [0.333 0.724 0.695 0.605];                      % MB %% Shared PK parameters for each drug     treatmentcourse_prtnrdrug = 100;     CQ_conv_fact = 319.872/1000;    % nM --> ng/ml    mdCQ_conv_fact = 291.819/1000;  % nM --> ng/ml    mef_conv_fact = (1000/378.312); % ng/ml --> nM    lum_conv_fact = (1000/528.939); % ng/ml --> nM     resis_fact_IC50     = 1;                                resis_fact_slope    = 1;     numofruns_PKPD = numofruns;        if strcmp(drug,'LMF')            RRsnddrug = log10(1/3000);      %Maximum reduction ratio of second drug            tmt_absorption_failure = .05;            upd_incrmnt = 1; % upd_incrmnt = 60;                                   gamma               = 2.5;            MIC_lum             = (350*66/170) / 2.^(1./gamma); % 235 / 2.^(1./gamma);                                                     C_50_lum            = (350*66/170); % 235; %IC50 in ng/ml;                                                    p_model             = [gamma C_50_lum];                                                                    adjalley_5x_const   = 66*5/lum_conv_fact;                                                         % from Adjalley et al. nM --> ng/ml                                           % With full treatment, Cdaily_drug is above this value for ? days...    end     if strcmp(drug,'CQ')            RRfstdrug           = 1;            RRsnddrug           = log10(1/3000); % -3;            full_DHA_day        = 0;                   gamma               = 2.5;            MIC_CQ              = (350*25/170) * resis_fact_IC50 / 2.^(1./gamma); % 90 * resis_fact_IC50 / 2.^(1./gamma);            C_50_cq             = (350*25/170) * resis_fact_IC50; % 90 * resis_fact_IC50; %IC50 in ng/ml;            p_model             = [gamma C_50_cq];                                     adjalley_5x_const   = 25 * CQ_conv_fact * 5;                  % from "Chloroquine Resistance in Plasmodium falciparum Malaria Parasites Conferred by pfcrt Mutations" nM --> ng/ml             % With full treatment, Cdaily_drug is above this value for 14 days... conc_mn_cq + conc_mn_mdcq                                               end_tm_cq = 100;                       rd_data = xlsread('CQ.xls');                hr = rd_data(:,1);                conc = rd_data(:,2);            rd_data = xlsread('mdCQ.xls');                hr_mdCQ = rd_data(:,1);                conc_mdCQ = rd_data(:,2);            k_1_2_1 = 55;            k_1_2_2 = 233;            break_point = 6;                          end                  if strcmp(drug,'MEF') && strcmp(MEF_type,'fixed')            RRsnddrug = log10(1/300);      %Maximum reduction ratio of second drug             gamma               = 2.5;            MIC_mef             = 600 ./ (2.^(1./gamma));            C_50_mef            = 600;                              %IC50 in ng/ml;            p_model             = [gamma C_50_mef];                                 adjalley_5x_const   = 170.4*5/mef_conv_fact;                  % from Adjalley et al. nM --> ng/ml            % With full loose treatment, Cdaily_drug is above this value for 40 days...             k_1_2_1 = 104;            %k_1_2_2 = 290;            k_1_2_2 = 347;            break_point = 8;    end      if strcmp(drug,'MEF') && strcmp(MEF_type,'loose')            RRsnddrug = log10(1/300);       %Maximum reduction ratio of second drug            gamma               = 2.5;            MIC_mef             = 600 ./ (2.^(1./gamma));            C_50_mef            = 600;                              %IC50 in ng/ml;            p_model             = [gamma C_50_mef];                                 adjalley_5x_const   = 170.4*5/mef_conv_fact;                  % from Adjalley et al. nM --> ng/ml            % With full loose treatment, Cdaily_drug is above this value for 40 days...            k_1_2_1 = 120;            %k_1_2_2 = 290;            k_1_2_2 = 295;            break_point = 8;    end %% Initialize mem     Cdaily_drug = zeros(numofruns_PKPD,treatmentcourse_prtnrdrug+1);         Cdaily_drug_pretreat = [];     mn_PD = zeros(numofruns_PKPD,treatmentcourse_prtnrdrug+1);  %% Initialize progressbar    % progressbar('Simulations') %% Number of repeats     number_of_rpts = @(x,y) ((x+1)./2 - 1)./(1 - ((1-y)./2 + y)); %% Standard PK      Cdaily_drug_standard = zeros(1,treatmentcourse_prtnrdrug+1);       % total_conc = zeros(1,treatmentcourse_prtnrdrug+1);          % LMF         % Notation:        % C_lum --> total_conc        % x_C_lum        % conc_mn_LUM --> Cdaily_drug         if strcmp(drug,'LMF')                          for j = 1:1                                 endtmhr = (treatmentcourse_prtnrdrug+1)*24;                                C_lum = zeros(1,endtmhr*upd_incrmnt);                                         %% Alternative model specification                                                        % Pharmacokinetics and Pharmacodynamics of Lumefantrine(Benflumetol) in                                                        % Acute Falciparum Malaria (PPL)                                                        % 51 patients Bangkok, 215 Mae La                                                        % 18 patients Bangkok, 72 Mae La <-- regimen B                                         %% Calculate stochastic constants                                         %Residual plots (not provided)                                        %indicated that there was no bias in estimation and that                                        %patient-specific profiles were characterized adequately (data                                        %not shown)                                             % Constant F1-6                                                                                        rand_mult_F = 1;                                             % Volume                                             rand_mult_V = 1;                                                                                        % Constant Ka: The bigger this constant, the smaller Cmax                                             rand_mult_Ka = 1;                                                                                        % Constant alpha: DONE                                             rand_mult_alpha = 1;                                                                                  %% Calculate PK parameters                                                                                                        %Constant                                               Source                                                        ka      = rand_mult_Ka .* 0.17;                 %absorption constant                                    PPL                                                        alpha   = rand_mult_alpha .* 0.114;             %initial plasma phase elimination constant              PPL                                                        beta    = 0.009;                                %terminal plasma phase elimination constant             PPL                                                        k21     = 0.015;                                %rate from peripheral to central compartment            PPL                                                        V       = rand_mult_V .* 103;                   %volume of distribution of the central compartment      PPL                                                            L       = [0 8 24 32 48 56];                    %dosage times (in hours)                                Coartem dosing card                                                        lag     = 2;                                    %lag time                                               PPL                                                            F1      = rand_mult_F .* (1.0)    *(4*120*1000);                                                                                                     F2      = rand_mult_F .* (.51)    *(4*120*1000);                                                                                                     F3      = rand_mult_F .* (1.49)   *(4*120*1000);                                                                                                   F4      = rand_mult_F .* (.51)    *(4*120*1000);                                                                                                   F5      = rand_mult_F .* (2.54)   *(4*120*1000);                                                                                                    F6      = rand_mult_F .* (1.68)   *(4*120*1000);  % 1.1 for Bangkok, more severe                                                           %F5      = 0;           Novartis four-dose test                                                                                                     %F6      = 0;  % 1.1    Novartis four-dose test                                          %% Conversion to Standard Form                                                         A = (ka/V)*(k21-alpha)/((ka-alpha)*(beta-alpha));                                                        B = (ka/V)*(k21-beta)/((ka-beta)*(alpha-beta));                                         %% Equations describing drug dynamics                                                        C1 = @(t)  F1*( A * exp( -alpha*(t - L(1) - lag) )+ B *...                                                        exp( -beta*(t - L(1) - lag) ) - (A + B) * exp( -ka*(t - L(1) -lag) ) );                                                         C2 = @(t)  F1*( A * exp( -alpha*(t - L(1) - lag) )+ B *...                                                        exp( -beta*(t - L(1) - lag) ) - (A + B) * exp( -ka*(t - L(1) -lag) ) ) +...                                                                    F2*( A * exp( -alpha*(t - L(2) - lag) )+ B *...                                                        exp( -beta*(t - L(2) - lag) ) - (A + B) * exp( -ka*(t - L(2) -lag) ) );                                                         C3 = @(t)  F1*( A * exp( -alpha*(t - L(1) - lag) )+ B *...                                                        exp( -beta*(t - L(1) - lag) ) - (A + B) * exp( -ka*(t - L(1) -lag) ) ) +...                                                                    F2*( A * exp( -alpha*(t - L(2) - lag) )+ B *...                                                        exp( -beta*(t - L(2) - lag) ) - (A + B) * exp( -ka*(t - L(2) -lag) ) ) +...                                                                    F3*( A * exp( -alpha*(t - L(3) - lag) )+ B *...                                                        exp( -beta*(t - L(3) - lag) ) - (A + B) * exp( -ka*(t - L(3) -lag) ) );                                                         C4 = @(t)  F1*( A * exp( -alpha*(t - L(1) - lag) )+ B *...                                                        exp( -beta*(t - L(1) - lag) ) - (A + B) * exp( -ka*(t - L(1) -lag) ) ) +...                                                                    F2*( A * exp( -alpha*(t - L(2) - lag) )+ B *...                                                        exp( -beta*(t - L(2) - lag) ) - (A + B) * exp( -ka*(t - L(2) -lag) ) ) +...                                                                    F3*( A * exp( -alpha*(t - L(3) - lag) )+ B *...                                                        exp( -beta*(t - L(3) - lag) ) - (A + B) * exp( -ka*(t - L(3) -lag) ) ) +...                                                                    F4*( A * exp( -alpha*(t - L(4) - lag) )+ B *...                                                        exp( -beta*(t - L(4) - lag) ) - (A + B) * exp( -ka*(t - L(4) -lag) ) );                                                         C5 = @(t)  F1*( A * exp( -alpha*(t - L(1) - lag) )+ B *...                                                        exp( -beta*(t - L(1) - lag) ) - (A + B) * exp( -ka*(t - L(1) -lag) ) ) +...                                                                    F2*( A * exp( -alpha*(t - L(2) - lag) )+ B *...                                                        exp( -beta*(t - L(2) - lag) ) - (A + B) * exp( -ka*(t - L(2) -lag) ) ) +...                                                                    F3*( A * exp( -alpha*(t - L(3) - lag) )+ B *...                                                        exp( -beta*(t - L(3) - lag) ) - (A + B) * exp( -ka*(t - L(3) -lag) ) ) +...                                                                    F4*( A * exp( -alpha*(t - L(4) - lag) )+ B *...                                                        exp( -beta*(t - L(4) - lag) ) - (A + B) * exp( -ka*(t - L(4) -lag) ) ) +...                                                                    F5*( A * exp( -alpha*(t - L(5) - lag) )+ B *...                                                        exp( -beta*(t - L(5) - lag) ) - (A + B) * exp( -ka*(t - L(5) -lag) ) );                                                         C6 = @(t)  F1*( A * exp( -alpha*(t - L(1) - lag) )+ B *...                                                        exp( -beta*(t - L(1) - lag) ) - (A + B) * exp( -ka*(t - L(1) -lag) ) ) +...                                                                    F2*( A * exp( -alpha*(t - L(2) - lag) )+ B *...                                                        exp( -beta*(t - L(2) - lag) ) - (A + B) * exp( -ka*(t - L(2) -lag) ) ) +...                                                                    F3*( A * exp( -alpha*(t - L(3) - lag) )+ B *...                                                        exp( -beta*(t - L(3) - lag) ) - (A + B) * exp( -ka*(t - L(3) -lag) ) ) +...                                                                    F4*( A * exp( -alpha*(t - L(4) - lag) )+ B *...                                                        exp( -beta*(t - L(4) - lag) ) - (A + B) * exp( -ka*(t - L(4) -lag) ) ) +...                                                                    F5*( A * exp( -alpha*(t - L(5) - lag) )+ B *...                                                        exp( -beta*(t - L(5) - lag) ) - (A + B) * exp( -ka*(t - L(5) -lag) ) ) +...                                                                    F6*( A * exp( -alpha*(t - L(6) - lag) )+ B *...                                                        exp( -beta*(t - L(6) - lag) ) - (A + B) * exp( -ka*(t - L(6) -lag) ) );                                         %% Calculating daily plasma levels (units are hours)                                                         counter = 1;                                                        for t = 1:(endtmhr*upd_incrmnt) % loop variable calculates plasma concentrations in minutes, starting at minute 1                                                             if t>=1 && t<=((L(1)*upd_incrmnt+lag*upd_incrmnt))                                                                       C_lum(1,t) = 0;                                                                end                                                                if t>((L(1)*upd_incrmnt+lag*upd_incrmnt)) && t<=((L(2)*upd_incrmnt+lag*upd_incrmnt))                                                                        C_lum(1,counter)  = C1((t/upd_incrmnt));                                                               end                                                                 if t>((L(2)*upd_incrmnt+lag*upd_incrmnt)) && t<=((L(3)*upd_incrmnt+lag*upd_incrmnt))                                                                        C_lum(1,counter)  = C2((t/upd_incrmnt));                                                                end                                                            if t>((L(3)*upd_incrmnt+lag*upd_incrmnt)) && t<=((L(4)*upd_incrmnt+lag*upd_incrmnt))                                                                      C_lum(1,counter)  = C3((t/upd_incrmnt));                                                               end                                                               if t>((L(4)*upd_incrmnt+lag*upd_incrmnt)) && t<=((L(5)*upd_incrmnt+lag*upd_incrmnt))                                                                       C_lum(1,counter)  = C4((t/upd_incrmnt));                                                                end                                                               if t>((L(5)*upd_incrmnt+lag*upd_incrmnt)) && t<=((L(6)*upd_incrmnt+lag*upd_incrmnt))                                                                       C_lum(1,counter)  = C5((t/upd_incrmnt));                                                              end                                                                if t>((L(6)*upd_incrmnt+lag*upd_incrmnt))                                                                       C_lum(1,counter)  = C6((t/upd_incrmnt));                                                               end                                                           counter = counter + 1;                                                        end                                                        counter = 0;                                         %% Error Correction                                                         for i = 1:length(C_lum(1,:))                                                            if C_lum(1,i)<0                                                                C_lum(1,i) = 0;                                                            end                                                        end                                         %% Save C_lum, compute PD                                                 C_lum = cat(2,0,C_lum); % include 0th minute                                                x_C_lum = 0:((treatmentcourse_prtnrdrug+1)*24*upd_incrmnt);                                          %% Calculate daily drug levels                                             for ctr = 1:1                                                         for i = 1:treatmentcourse_prtnrdrug+1                                                                                        conc_mn_LUM(ctr,i) = mean(C_lum(ctr,find(((0 + 24*(i-1)) <= x_C_lum/upd_incrmnt) .* (x_C_lum/upd_incrmnt <= (24 + 24*(i-1)) ) ==  1)));                                                        end                                              end                                         total_conc(j,:) = C_lum;                                                                      Cdaily_drug_standard(j,:) = conc_mn_LUM;                         end % end of the individual LMF loop                         %% Include the effects of treatment absorption failure                         % index = 1:length(rand_mult_holder);                        % index(rand_mult_holder==min(rand_mult_holder));                         % if random('unif',0,1)<tmt_absorption_failure                           %   total_conc(ind,:) = 0.*total_conc(ind,:);                                    % end         end % end of LMF loop      % CQ         % Notation:        % CQ_interp + mdCQ_interp --> total_conc_CQ + total_conc_mdCQ --> total_conc        % x_C_cq        % conc_mn_CQ + conc_mn_mdCQ --> Cdaily_drug         if strcmp(drug,'CQ')                        adult_to_child = 1.5/.564;      % convert from child to adult                                        for j = 1:1                                                         CQ_y(1)= 10^-5;                                                        CQ_x = hr./24;                                                        CQ_y = conc;                                                         %% Stages                                                         CQ_x_first_stage = CQ_x(1:15)';                                                        CQ_y_first_stage = CQ_y(1:15)';                                                         %% Calculate stochastic constants                                                         % Factor 1: Tmax shift                                                         rand_mult_tmax = 1; % non-stochastic                                                         % Factor 2: Cmax shift                                                         rand_mult_Cmax = 1; % non-stochastic                                                                CQ_y(:,1) = CQ_y(:,1)' .* rand_mult_Cmax; % Multiplication by the Cmax constant %                                                                                                                % Factor 3: t1/2 shift                                                         rand_mult_t_clear_init = 1; % non-stochastic                                                         % Factor 4: t1/2 terminal shift                                                                                                                rand_mult_t_clear_term = 1; % non-stochastic                                                         %% Dilation Effect                                                         CQ_x = CQ_x_first_stage .* rand_mult_tmax; % in days                                                         %% Extrapolating Plasma Concetrations, inital clearance                                                         k = log(1/2)/(k_1_2_1/24) * rand_mult_t_clear_init; %If we want an hourly rate, we can take hourly factor directly                                                         end_tm_cq = break_point;                                                         diff = end_tm_cq-max(CQ_x);                                                         clear new_x new_y                                                                     for i = 1: floor(diff) % no +1 here                                                                         new_x(i) = max(CQ_x) + i ;                                                                         new_y(i) = CQ_y(15,:) * exp(k * i);                                                                     end                                                         CQ_x = cat(2,CQ_x,new_x);                                                         CQ_y = cat(2,CQ_y(1:15,1)',new_y);                                                         %% Extrapolating Plasma Concetrations, terminal clearance                                                         k = log(1/2)/(k_1_2_2/24) * rand_mult_t_clear_term; %If we want an hourly rate, we can take hourly factor directly                                                         end_tm_cq = 102;                                                         diff = end_tm_cq-max(CQ_x);                                                         clear new_x new_y                                                                     for i = 1: floor(diff)+1 % REVISED                                                                         new_x(i) = max(CQ_x) + i ;                                                                         new_y(i) = CQ_y(1,length(CQ_y)) * exp(k * i);                                                                     end                                                         CQ_x = cat(2,CQ_x,new_x);                                                         CQ_y = cat(2,CQ_y,new_y);                                                         %% Define x_C_cq                                                                                                                    num_of_daily_samples = 24; % Sample hourly                                                            initval = 0;                                                            x_C_cq = initval:1/num_of_daily_samples:(end_tm_cq);                                                                                                                     %% Interpolating Plasma Concetrations                                                                        for ctr = 1:1                                                                        CQ_interp(ctr,:) = interp1(CQ_x,log(CQ_y(ctr,:)),x_C_cq,'linear');                                                                     end                                                                     CQ_interp = exp(CQ_interp);                                                                                                                             %% Calculating daily drug levels                                                                     for ctr = 1:1                                                                        for i = 1:treatmentcourse_prtnrdrug+1                                                                                                        conc_mn_CQ(ctr,i) = mean(CQ_interp(ctr,find(((0 + (i-1)) <= x_C_cq) .* (x_C_cq <= (1 + (i-1)) ) ==  1)));                                                                        end                                                                      end                                 k_1_2_1 = 60;                            k_1_2_2 = 290;                            break_point = 7;                                                             mdCQ_y(1)= 10^-5;                                                            % mdCQ_y(2)= 10^-5;                                                            mdCQ_x = hr_mdCQ./24;                                                            mdCQ_y = conc_mdCQ;                                                         %% Stages                                                         mdCQ_x_first_stage = mdCQ_x(1:5)';                                                        mdCQ_y_first_stage = mdCQ_y(1:5)';                                                         mdCQ_y(:,1) = mdCQ_y(:,1)' .* rand_mult_Cmax; % Multiplication by the Cmax constant %                                                           %% Dilation Effect                                                         mdCQ_x = mdCQ_x_first_stage .* rand_mult_tmax; % in days                                                         %% Extrapolating Plasma Concetrations, inital clearance                                                         k = log(1/2)/(k_1_2_1/24) * rand_mult_t_clear_init; %If we want an hourly rate, we can take hourly factor directly                                                         end_tm_cq = break_point;                                                         diff = end_tm_cq-max(mdCQ_x);                                                         clear new_x new_y                                                                     for i = 1: floor(diff) % no +1 here                                                                         new_x(i) = max(mdCQ_x) + i ;                                                                         new_y(i) = mdCQ_y(5,:) * exp(k * i);                                                                     end                                                         mdCQ_x = cat(2,mdCQ_x,new_x);                                                         mdCQ_y = cat(2,mdCQ_y(1:5,1)',new_y);                                                         %% Extrapolating Plasma Concetrations, terminal clearance                                                         k = log(1/2)/(k_1_2_2/24) * rand_mult_t_clear_term; %If we want an hourly rate, we can take hourly factor directly                                                         end_tm_cq = 102;                                                         diff = end_tm_cq-max(mdCQ_x);                                                         clear new_x new_y                                                                     for i = 1: floor(diff)+1 % REVISED                                                                         new_x(i) = max(mdCQ_x) + i ;                                                                         new_y(i) = mdCQ_y(1,length(mdCQ_y)) * exp(k * i);                                                                     end                                                         mdCQ_x = cat(2,mdCQ_x,new_x);                                                         mdCQ_y = cat(2,mdCQ_y,new_y);                                                         %% Define x_C_cq                                                                                                                    num_of_daily_samples = 24; % Sample hourly                                                            initval = 0;                                                            x_C_cq = initval:1/num_of_daily_samples:(end_tm_cq);                                                                                                                     %% Interpolating Plasma Concetrations                                                                     for ctr = 1:1                                                                        mdCQ_interp(ctr,:) = interp1(mdCQ_x,log(mdCQ_y(ctr,:)),x_C_cq,'linear');                                                                     end                                                                     mdCQ_interp = exp(mdCQ_interp);                                                          %% Calculating daily drug levels                                                                     for ctr = 1:1                                                                        for i = 1:treatmentcourse_prtnrdrug+1                                                                                                        conc_mn_mdCQ(ctr,i) = mean(mdCQ_interp(ctr,find(((0 + (i-1)) <= x_C_cq) .* (x_C_cq <= (1 + (i-1)) ) ==  1)));                                                                        end                                                                      end                                 total_conc_CQ(j,:) = CQ_interp(1,:);                             total_conc_mdCQ(j,:) = mdCQ_interp(1,:);                                                         Cdaily_drug_standard(j,:) = conc_mn_CQ + conc_mn_mdCQ;                             end % end of the individual loop                             %% Convert from child nM to adult ng/ml                            Cdaily_drug_standard = Cdaily_drug_standard.*CQ_conv_fact.*adult_to_child;                                   total_conc = total_conc_CQ + total_conc_mdCQ;                               %% Convert from child nM to adult ng/ml                             total_conc = total_conc .* CQ_conv_fact.*adult_to_child;                                    end % end of CQ loop             % MFQ_fixed         % Notation:        % MEF_interp --> total_conc        % x_C_mef        % conc_mn_MEF --> Cdaily_drug                 if strcmp(drug,'MEF') && strcmp(MEF_type,'fixed')                        for j = 1:1                                                          load MEF_PK_fixed.mat                                      %DELETE!!!!!!!!!!!                                                        MEF_y(1)= 10^-5;                                                        % MEF_y(2)= 10^-5;                                                        % mfq_cnst = 20;                                             %DELETE!!!!!!!!!!!                                                        MEF_x = MEF_x'./24; % day 28!                                                        MEF_y_hldr = MEF_y;                                                        % MEF_y = zeros(mfq_cnst,numofruns_PKPD);                                             %% Stages                                                         % MEF_x_first_stage = MEF_x(1:15)'./24; <-- Hmmm...                                                        MEF_x_first_stage = MEF_x(1:15)';                                                        MEF_y_first_stage = MEF_y(1:15);                                             %% Calculate stochastic constants                                             % 2 - 1/2: fails symmetry test (geomean and mean: geomean([1.5 1/1.5]) geomean([mean([1 1.5]) mean([1 1/1.5])]))                                            % 2 - 0: passes mean test, fails geomean, fails for greater than 2                                            % 2 - 1/2: skewed                                             % Factor 1: Tmax shift                                             rand_mult_tmax = 1; % non-stochastic                                             % Factor 2: Cmax shift                                             rand_mult_Cmax = 1; % non-stochastic                                             MEF_y(1,:) = MEF_y_hldr .* rand_mult_Cmax;                                             % Factor 3: t1/2 shift                                             rand_mult_t_clear_init = 1; % non-stochastic                                                                                       % Factor 4: t1/2 terminal shift                                             rand_mult_t_clear_term = 1; % non-stochastic                                             %% Dilation Effect                                             MEF_x = MEF_x_first_stage .* rand_mult_tmax;                                             %% Extrapolating Plasma Concetrations, inital clearance                                             k = log(1/2)/(k_1_2_1/24) * rand_mult_t_clear_init; %If we want an hourly rate, we can take hourly factor directly                                             end_tm_mef = break_point;                                             diff = end_tm_mef-max(MEF_x);                                             clear new_x new_y                                                         for i = 1: floor(diff)                                                             new_x(i) = max(MEF_x) + i ;                                                             new_y(i) = MEF_y(:,15) * exp(k * i);                                                         end                                             MEF_x = cat(2,MEF_x,new_x);                                             MEF_y = cat(2,MEF_y(1,1:15),new_y);                                             %% Extrapolating Plasma Concetrations, terminal clearance                                             k = log(1/2)/(k_1_2_2/24) * rand_mult_t_clear_term; %If we want an hourly rate, we can take hourly factor directly                                             end_tm_mef = 102;                                             diff = end_tm_mef-max(MEF_x);                                             clear new_x new_y                                                         for i = 1: floor(diff)                                                             new_x(i) = max(MEF_x) + i ;                                                             new_y(i) = MEF_y(1,length(MEF_y)) * exp(k * i);                                                         end                                             MEF_x = cat(2,MEF_x,new_x);                                             MEF_y = cat(2,MEF_y,new_y);                                             end_tm_mef = 100;                                             %% Interpolating Plasma Concetrations                                                         num_of_daily_samples = 24; % Sample hourly                                                        initval = 0;                                                        for ctr = 1:1                                                            MEF_interp(ctr,:) = interp1(MEF_x,log(MEF_y(ctr,:)),initval:1/num_of_daily_samples:(end_tm_mef+1),'linear');                                                         end                                                         MEF_interp = exp(MEF_interp);                                              %% Save MEF_interp, compute PD                                                 x_C_mef = initval:1/num_of_daily_samples:(end_tm_mef+1);                                              %% Calculating daily drug levels                                                         for ctr = 1:1                                                            for i = 1:treatmentcourse_prtnrdrug+1                                                                                            conc_mn_MEF(ctr,i) = mean(MEF_interp(ctr,find(((0 + (i-1)) <= x_C_mef) .* (x_C_mef <= (1 + (i-1)) ) ==  1)));                                                            end                                                          end                                      total_conc(j,:) = MEF_interp(1,:);                                                                                Cdaily_drug_standard(j,:) = conc_mn_MEF;                         end         end %MFQ_fixed                             % MFQ_loose         % Notation:        % MEF_interp --> total_conc        % x_C_mef        % conc_mn_MEF --> Cdaily_drug         if strcmp(drug,'MEF') && strcmp(MEF_type,'loose')                        for j = 1:1                                                          load MEF_PK_loose.mat                                      %DELETE!!!!!!!!!!!                                                        MEF_y(1)= 10^-5;                                                        MEF_y(2)= 10^-5;                                                        % mfq_cnst = 20;                                             %DELETE!!!!!!!!!!!                                                        MEF_x = MEF_x'./24; % day 28!                                                        MEF_y_hldr = MEF_y;                                                        % MEF_y = zeros(mfq_cnst,numofruns_PKPD);                                             %% Stages                                                         % MEF_x_first_stage = MEF_x(1:4)'./24; <-- Hmmm...                                                        MEF_x_first_stage = MEF_x(1:4)';                                                        MEF_y_first_stage = MEF_y(1:4);                                             %% Calculate stochastic constants                                             % 2 - 1/2: fails symmetry test (geomean and mean: geomean([1.5 1/1.5]) geomean([mean([1 1.5]) mean([1 1/1.5])]))                                            % 2 - 0: passes mean test, fails geomean, fails for greater than 2                                            % 2 - 1/2: skewed                                             % Factor 1: Tmax shift                                             rand_mult_tmax = 1; % non-stochastic                                             % Factor 2: Cmax shift                                             rand_mult_Cmax = 1; % non-stochastic                                                                                                           MEF_y(1,:) = MEF_y_hldr .* rand_mult_Cmax;                                                % Factor 3: t1/2 shift                                             rand_mult_t_clear_init = 1; % non-stochastic                                                                                        % Factor 4: t1/2 terminal shift                                                                                        rand_mult_t_clear_term = 1; % non-stochastic                                             %% Dilation Effect                                             MEF_x = MEF_x_first_stage .* rand_mult_tmax;                                             %% Extrapolating Plasma Concetrations, inital clearance                                             k = log(1/2)/(k_1_2_1/24) * rand_mult_t_clear_init; %If we want an hourly rate, we can take hourly factor directly                                             end_tm_mef = break_point;                                             diff = end_tm_mef-max(MEF_x);                                             clear new_x new_y                                                         for i = 1: floor(diff)                                                             new_x(i) = max(MEF_x) + i ;                                                             new_y(i) = MEF_y(:,4) * exp(k * i);                                                         end                                             MEF_x = cat(2,MEF_x,new_x);                                             MEF_y = cat(2,MEF_y(1,1:4),new_y);                                             %% Extrapolating Plasma Concetrations, terminal clearance                                             k = log(1/2)/(k_1_2_2/24) * rand_mult_t_clear_term; %If we want an hourly rate, we can take hourly factor directly                                             end_tm_mef = 102;                                             diff = end_tm_mef-max(MEF_x);                                             clear new_x new_y                                                         for i = 1: floor(diff)                                                             new_x(i) = max(MEF_x) + i ;                                                             new_y(i) = MEF_y(1,length(MEF_y)) * exp(k * i);                                                         end                                             MEF_x = cat(2,MEF_x,new_x);                                             MEF_y = cat(2,MEF_y,new_y);                                             end_tm_mef = 100;                                             %% Interpolating Plasma Concetrations                                                         num_of_daily_samples = 24; % Sample hourly                                                        initval = 0;                                                        for ctr = 1:1                                                            MEF_interp(ctr,:) = interp1(MEF_x,log(MEF_y(ctr,:)),initval:1/num_of_daily_samples:(end_tm_mef+1),'linear');                                                         end                                                         MEF_interp = exp(MEF_interp);                                              %% Save MEF_interp, compute PD                                                 x_C_mef = initval:1/num_of_daily_samples:(end_tm_mef+1);                                              %% Calculating daily drug levels                                                         for ctr = 1:1                                                            for i = 1:treatmentcourse_prtnrdrug+1                                                                                            conc_mn_MEF(ctr,i) = mean(MEF_interp(ctr,find(((0 + (i-1)) <= x_C_mef) .* (x_C_mef <= (1 + (i-1)) ) ==  1)));                                                            end                                                          end                             total_conc(j,:) = MEF_interp(1,:);                                                                                Cdaily_drug_standard(j,:) = conc_mn_MEF;                         end         end %MFQ_loose      if rand_PK == 0        for j = 1:numofruns_PKPD            Cdaily_drug(j,:) = Cdaily_drug_standard;            rand_mult_holder_t_clear_term(j) = 1;        end    end  %% Stochastic PK      if rand_PK == 1 || random_pretreat == 1        % LMF         % Notation:        % C_lum --> total_conc        % x_C_lum        % conc_mn_LUM --> Cdaily_drug         if strcmp(drug,'LMF')                          for j = 1:numofruns_PKPD                                 rand_mult_holder = zeros(1,numofruns_PKPD);                                endtmhr = (treatmentcourse_prtnrdrug+1)*24;                                C_lum = zeros(1,endtmhr*upd_incrmnt);                                         %% Alternative model specification                                                        % Pharmacokinetics and Pharmacodynamics of Lumefantrine(Benflumetol) in                                                        % Acute Falciparum Malaria (PPL)                                                        % 51 patients Bangkok, 215 Mae La                                                        % 18 patients Bangkok, 72 Mae La <-- regimen B                                         %% Calculate stochastic constants                                         %Residual plots (not provided)                                        %indicated that there was no bias in estimation and that                                        %patient-specific profiles were characterized adequately (data                                        %not shown)                                             % Constant F1-6                                             if rand_PK == 1                                                                                                            upper_lim = 1.5; % bounded above                                                            lower_lim = 3; % bounded below                                                             rand_mult = random('unif',1,upper_lim);     %Upper plasma levels raised by factor of 3                                                            rand_mult_1 = random('unif',1,lower_lim);   %Lower plasma levels decreased by factor of 9                                                            rand_mult_2 = random('unif',0,1);                                                             if rand_mult_2 < .5                                                                rand_mult_F = rand_mult;                                                            end                                                             if rand_mult_2 >= .5                                                                rand_mult_F = 1/rand_mult_1;                                                            end                                                             rand_mult_holder_F(j) = rand_mult_F;                                             else                                                            rand_mult_F = 1;                                            end                                                                                        % Volume                                             if rand_PK == 1                                                                                                            upper_lim = 1.5; % bounded above                                                            lower_lim = 1.5; % bounded below                                                             rand_mult = random('unif',1,upper_lim);     %Upper plasma levels raised by factor of 3                                                            rand_mult_1 = random('unif',1,lower_lim);   %Lower plasma levels decreased by factor of 9                                                            rand_mult_2 = random('unif',0,1);                                                             if rand_mult_2 < .5                                                                rand_mult_V = rand_mult;                                                            end                                                             if rand_mult_2 >= .5                                                                rand_mult_V = 1/rand_mult_1;                                                            end                                                             rand_mult_holder_V(j) = rand_mult_V;                                             else                                                            rand_mult_V = 1;                                            end                                                                                        % Constant Ka: The bigger this constant, the smaller Cmax                                             if rand_PK == 1                                                                                                            upper_lim = 1.5;                                                             lower_lim = 1.5;                                                             rand_mult = random('unif',1,upper_lim);     %Upper plasma levels raised by factor of 3                                                            rand_mult_1 = random('unif',1,lower_lim);   %Lower plasma levels decreased by factor of 9                                                            rand_mult_2 = random('unif',0,1);                                                             if rand_mult_2 < .5                                                                rand_mult_Ka = rand_mult;                                                            end                                                             if rand_mult_2 >= .5                                                                rand_mult_Ka = 1/rand_mult_1;                                                            end                                                             rand_mult_holder_Ka(j) = rand_mult_Ka;                                            else                                                            rand_mult_Ka = 1;                                            end                                                                                        % Constant alpha: DONE                                             if rand_PK == 1                                                                                                            upper_lim = 1.1;                                                            lower_lim = 2;                                                             rand_mult = random('unif',1,upper_lim);     %Upper plasma levels raised by factor of 3                                                            rand_mult_1 = random('unif',1,lower_lim);   %Lower plasma levels decreased by factor of 9                                                            rand_mult_2 = random('unif',0,1);                                                             if rand_mult_2 < .5                                                                rand_mult_alpha = rand_mult;                                                            end                                                             if rand_mult_2 >= .5                                                                rand_mult_alpha = 1/rand_mult_1;                                                            end                                                             rand_mult_holder_alpha(j) = rand_mult_alpha;                                             else                                                            rand_mult_alpha = 1;                                            end                                         %% Calculate PK parameters                                                                                                        %Constant                                               Source                                                        ka      = rand_mult_Ka .* 0.17;                 %absorption constant                                    PPL                                                        alpha   = rand_mult_alpha .* 0.114;             %initial plasma phase elimination constant              PPL                                                        beta    = 0.009;                                %terminal plasma phase elimination constant             PPL                                                        k21     = 0.015;                                %rate from peripheral to central compartment            PPL                                                        V       = rand_mult_V .* 103;                   %volume of distribution of the central compartment      PPL                                                            L       = [0 8 24 32 48 56];                    %dosage times (in hours)                                Coartem dosing card                                                        lag     = 2;                                    %lag time                                               PPL                                                            F1      = rand_mult_F .* (1.0)    *(4*120*1000);                                                                                                     F2      = rand_mult_F .* (.51)    *(4*120*1000);                                                                                                     F3      = rand_mult_F .* (1.49)   *(4*120*1000);                                                                                                   F4      = rand_mult_F .* (.51)    *(4*120*1000);                                                                                                   F5      = rand_mult_F .* (2.54)   *(4*120*1000);                                                                                                    F6      = rand_mult_F .* (1.68)   *(4*120*1000);  % 1.1 for Bangkok, more severe                                                           %F5      = 0;                                                                                                    %F6      = 0;  % 1.1                                                                                                ka_holder(j) = ka;                                        alpha_holder(j) = alpha;                                        V_holder(j) = V;                                        F1_holder(j) = F1;                                                       F2_holder(j) = F2;                                        F3_holder(j) = F3;                                        F4_holder(j) = F4;                                        F5_holder(j) = F5;                                        F6_holder(j) = F6;                                         %% Conversion to Standard Form                                                         A = (ka/V)*(k21-alpha)/((ka-alpha)*(beta-alpha));                                                        B = (ka/V)*(k21-beta)/((ka-beta)*(alpha-beta));                                         %% Equations describing drug dynamics                                                        C1 = @(t)  F1*( A * exp( -alpha*(t - L(1) - lag) )+ B *...                                                        exp( -beta*(t - L(1) - lag) ) - (A + B) * exp( -ka*(t - L(1) -lag) ) );                                                         C2 = @(t)  F1*( A * exp( -alpha*(t - L(1) - lag) )+ B *...                                                        exp( -beta*(t - L(1) - lag) ) - (A + B) * exp( -ka*(t - L(1) -lag) ) ) +...                                                                    F2*( A * exp( -alpha*(t - L(2) - lag) )+ B *...                                                        exp( -beta*(t - L(2) - lag) ) - (A + B) * exp( -ka*(t - L(2) -lag) ) );                                                         C3 = @(t)  F1*( A * exp( -alpha*(t - L(1) - lag) )+ B *...                                                        exp( -beta*(t - L(1) - lag) ) - (A + B) * exp( -ka*(t - L(1) -lag) ) ) +...                                                                    F2*( A * exp( -alpha*(t - L(2) - lag) )+ B *...                                                        exp( -beta*(t - L(2) - lag) ) - (A + B) * exp( -ka*(t - L(2) -lag) ) ) +...                                                                    F3*( A * exp( -alpha*(t - L(3) - lag) )+ B *...                                                        exp( -beta*(t - L(3) - lag) ) - (A + B) * exp( -ka*(t - L(3) -lag) ) );                                                         C4 = @(t)  F1*( A * exp( -alpha*(t - L(1) - lag) )+ B *...                                                        exp( -beta*(t - L(1) - lag) ) - (A + B) * exp( -ka*(t - L(1) -lag) ) ) +...                                                                    F2*( A * exp( -alpha*(t - L(2) - lag) )+ B *...                                                        exp( -beta*(t - L(2) - lag) ) - (A + B) * exp( -ka*(t - L(2) -lag) ) ) +...                                                                    F3*( A * exp( -alpha*(t - L(3) - lag) )+ B *...                                                        exp( -beta*(t - L(3) - lag) ) - (A + B) * exp( -ka*(t - L(3) -lag) ) ) +...                                                                    F4*( A * exp( -alpha*(t - L(4) - lag) )+ B *...                                                        exp( -beta*(t - L(4) - lag) ) - (A + B) * exp( -ka*(t - L(4) -lag) ) );                                                         C5 = @(t)  F1*( A * exp( -alpha*(t - L(1) - lag) )+ B *...                                                        exp( -beta*(t - L(1) - lag) ) - (A + B) * exp( -ka*(t - L(1) -lag) ) ) +...                                                                    F2*( A * exp( -alpha*(t - L(2) - lag) )+ B *...                                                        exp( -beta*(t - L(2) - lag) ) - (A + B) * exp( -ka*(t - L(2) -lag) ) ) +...                                                                    F3*( A * exp( -alpha*(t - L(3) - lag) )+ B *...                                                        exp( -beta*(t - L(3) - lag) ) - (A + B) * exp( -ka*(t - L(3) -lag) ) ) +...                                                                    F4*( A * exp( -alpha*(t - L(4) - lag) )+ B *...                                                        exp( -beta*(t - L(4) - lag) ) - (A + B) * exp( -ka*(t - L(4) -lag) ) ) +...                                                                    F5*( A * exp( -alpha*(t - L(5) - lag) )+ B *...                                                        exp( -beta*(t - L(5) - lag) ) - (A + B) * exp( -ka*(t - L(5) -lag) ) );                                                         C6 = @(t)  F1*( A * exp( -alpha*(t - L(1) - lag) )+ B *...                                                        exp( -beta*(t - L(1) - lag) ) - (A + B) * exp( -ka*(t - L(1) -lag) ) ) +...                                                                    F2*( A * exp( -alpha*(t - L(2) - lag) )+ B *...                                                        exp( -beta*(t - L(2) - lag) ) - (A + B) * exp( -ka*(t - L(2) -lag) ) ) +...                                                                    F3*( A * exp( -alpha*(t - L(3) - lag) )+ B *...                                                        exp( -beta*(t - L(3) - lag) ) - (A + B) * exp( -ka*(t - L(3) -lag) ) ) +...                                                                    F4*( A * exp( -alpha*(t - L(4) - lag) )+ B *...                                                        exp( -beta*(t - L(4) - lag) ) - (A + B) * exp( -ka*(t - L(4) -lag) ) ) +...                                                                    F5*( A * exp( -alpha*(t - L(5) - lag) )+ B *...                                                        exp( -beta*(t - L(5) - lag) ) - (A + B) * exp( -ka*(t - L(5) -lag) ) ) +...                                                                    F6*( A * exp( -alpha*(t - L(6) - lag) )+ B *...                                                        exp( -beta*(t - L(6) - lag) ) - (A + B) * exp( -ka*(t - L(6) -lag) ) );                                         %% Calculating daily plasma levels (units are hours)                                                         counter = 1;                                                        for t = 1:(endtmhr*upd_incrmnt) % loop variable calculates plasma concentrations in minutes, starting at minute 1                                                             if t>=1 && t<=((L(1)*upd_incrmnt+lag*upd_incrmnt))                                                                       C_lum(1,t) = 0;                                                                end                                                                if t>((L(1)*upd_incrmnt+lag*upd_incrmnt)) && t<=((L(2)*upd_incrmnt+lag*upd_incrmnt))                                                                        C_lum(1,counter)  = C1((t/upd_incrmnt));                                                               end                                                                 if t>((L(2)*upd_incrmnt+lag*upd_incrmnt)) && t<=((L(3)*upd_incrmnt+lag*upd_incrmnt))                                                                        C_lum(1,counter)  = C2((t/upd_incrmnt));                                                                end                                                            if t>((L(3)*upd_incrmnt+lag*upd_incrmnt)) && t<=((L(4)*upd_incrmnt+lag*upd_incrmnt))                                                                      C_lum(1,counter)  = C3((t/upd_incrmnt));                                                               end                                                               if t>((L(4)*upd_incrmnt+lag*upd_incrmnt)) && t<=((L(5)*upd_incrmnt+lag*upd_incrmnt))                                                                       C_lum(1,counter)  = C4((t/upd_incrmnt));                                                                end                                                               if t>((L(5)*upd_incrmnt+lag*upd_incrmnt)) && t<=((L(6)*upd_incrmnt+lag*upd_incrmnt))                                                                       C_lum(1,counter)  = C5((t/upd_incrmnt));                                                              end                                                                if t>((L(6)*upd_incrmnt+lag*upd_incrmnt))                                                                       C_lum(1,counter)  = C6((t/upd_incrmnt));                                                               end                                                           counter = counter + 1;                                                        end                                                        counter = 0;                                         %% Error Correction                                                         for i = 1:length(C_lum(1,:))                                                            if C_lum(1,i)<0                                                                C_lum(1,i) = 0;                                                            end                                                        end                                         %% Save C_lum, compute PD                                                 C_lum = cat(2,0,C_lum); % include 0th minute                                                x_C_lum = 0:((treatmentcourse_prtnrdrug+1)*24*upd_incrmnt);                                           %% Calculate daily drug levels                                             for ctr = 1:1                                                         for i = 1:treatmentcourse_prtnrdrug+1                                                                                        conc_mn_LUM(ctr,i) = mean(C_lum(ctr,find(((0 + 24*(i-1)) <= x_C_lum/upd_incrmnt) .* (x_C_lum/upd_incrmnt <= (24 + 24*(i-1)) ) ==  1)));                                                        end                                              end                         total_conc(j,:) = C_lum;                                                                    if rand_PK == 1                            Cdaily_drug(j,:) = conc_mn_LUM;                        end                                                    if random_pretreat == 1                            Cdaily_drug_pretreat(j,:) = conc_mn_LUM;                        end                                                end % end of the individual LMF loop                         %% Include the effects of treatment absorption failure                         % index = 1:length(rand_mult_holder);                        % index(rand_mult_holder==min(rand_mult_holder));                         % if random('unif',0,1)<tmt_absorption_failure                           %   total_conc(ind,:) = 0.*total_conc(ind,:);                                    % end         end % end of LMF loop      % CQ         % Notation:        % CQ_interp + mdCQ_interp --> total_conc_CQ + total_conc_mdCQ --> total_conc        % x_C_cq        % conc_mn_CQ + conc_mn_mdCQ --> Cdaily_drug         if strcmp(drug,'CQ')                        adult_to_child = 1.5/.564;      % convert from child to adult                                        for j = 1:numofruns_PKPD                                                         CQ_y(1)= 10^-5;                                                        CQ_x = hr./24;                                                        CQ_y = conc;                                                         %% Stages                                                         CQ_x_first_stage = CQ_x(1:15)';                                                        CQ_y_first_stage = CQ_y(1:15)';                                                         %% Calculate stochastic constants                                                         % Factor 1: Tmax shift                                                         if rand_PK == 1                                                                                                                        upper_lim = 1.3;                                                            lower_lim = 1/1.3;                                                             rand_mult = random('unif',1,upper_lim);     %Upper plasma levels raised by factor of 3                                                            rand_mult_1 = random('unif',lower_lim,1);   %Lower plasma levels decreased by factor of 9                                                              rand_mult_2 = random('unif',0,1);                                                             if rand_mult_2 < 1/(number_of_rpts(upper_lim,lower_lim)+1)                                                                rand_mult_tmax = rand_mult;                                                            end                                                             if rand_mult_2 >= 1/(number_of_rpts(upper_lim,lower_lim)+1)                                                                rand_mult_tmax = rand_mult_1;                                                                                        end                                                        else                                                            rand_mult_tmax = 1; % non-stochastic                                                        end                                                            rand_mult_holder_tmax(j) = rand_mult_tmax;                                                         % Factor 2: Cmax shift                                                                                                                 for ctr = 1:1                                                                                                                                                    if rand_PK == 1                                                                                                                            upper_lim = 1.9;                                                                lower_lim = 1/1.5;                                                                 rand_mult = random('unif',1,upper_lim);     %Upper plasma levels raised by factor of 3                                                                                           rand_mult_1 = random('unif',lower_lim,1);   %Lower plasma levels decreased by factor of 9                                                                                             rand_mult_2 = random('unif',0,1);                                                                                            if rand_mult_2 < 1/2                                                                                                rand_mult_Cmax = rand_mult;                                                                                           end                                                                                                                                 if rand_mult_2 >= 1/2                                                                                                 rand_mult_Cmax = rand_mult_1;                                                                                            end                                                                                                                                               else                                                                rand_mult_Cmax = 1; % non-stochastic                                                                   end                                                                                                                        CQ_y(:,ctr) = CQ_y(:,1)' .* rand_mult_Cmax; % Multiplication by the Cmax constant %                                                                   end                                                                                                                rand_mult_holder_Cmax(j) = rand_mult_Cmax;                                                                                                                                                                        % Factor 3: t1/2 shift                                                         if rand_PK == 1                                                                                                                        upper_lim = 1.7;                                                            lower_lim = 1;                                                             rand_mult = random('unif',1,upper_lim);     %Upper plasma levels raised by factor of 3                                                            rand_mult_1 = random('unif',lower_lim,1);   %Lower plasma levels decreased by factor of 9                                                              rand_mult_2 = random('unif',0,1);                                                             if rand_mult_2 < 1/2                                                                rand_mult_t_clear_init = rand_mult;                                                            end                                                             if rand_mult_2 >= 1/2                                                                rand_mult_t_clear_init = rand_mult_1;                                                                                        end                                                         else                                                            rand_mult_t_clear_init = 1; % non-stochastic                                                        end                                                            rand_mult_holder_t_clear_init(j) = rand_mult_t_clear_init;                                                         % Factor 4: t1/2 terminal shift                                                                                                                if rand_PK == 1                                                                upper_lim = 1.5;                                                            lower_lim = 1/1.5;                                                             rand_mult = random('unif',1,upper_lim);     %Upper plasma levels raised by factor of 3                                                            rand_mult_1 = random('unif',lower_lim,1);   %Lower plasma levels decreased by factor of 9                                                              rand_mult_2 = random('unif',0,1);                                                             if rand_mult_2 < 1/(number_of_rpts(upper_lim,lower_lim)+1)                                                                rand_mult_t_clear_term = rand_mult;                                                            end                                                             if rand_mult_2 >= 1/(number_of_rpts(upper_lim,lower_lim)+1)                                                                rand_mult_t_clear_term = rand_mult_1;                                                                                        end                                                        else                                                            rand_mult_t_clear_term = 1; % non-stochastic                                                        end                                                            rand_mult_holder_t_clear_term(j) = rand_mult_t_clear_term;                                                         %% Dilation Effect                                                         CQ_x = CQ_x_first_stage .* rand_mult_tmax; % in days                                                         %% Extrapolating Plasma Concetrations, inital clearance                                                         k = log(1/2)/(k_1_2_1/24) * rand_mult_t_clear_init; %If we want an hourly rate, we can take hourly factor directly                                                         end_tm_cq = break_point;                                                         diff = end_tm_cq-max(CQ_x);                                                         clear new_x new_y                                                                     for i = 1: floor(diff) % no +1 here                                                                         new_x(i) = max(CQ_x) + i ;                                                                         new_y(i) = CQ_y(15,:) * exp(k * i);                                                                     end                                                         CQ_x = cat(2,CQ_x,new_x);                                                         CQ_y = cat(2,CQ_y(1:15,1)',new_y);                                                         %% Extrapolating Plasma Concetrations, terminal clearance                                                         k = log(1/2)/(k_1_2_2/24) * rand_mult_t_clear_term; %If we want an hourly rate, we can take hourly factor directly                                                         end_tm_cq = 102;                                                         diff = end_tm_cq-max(CQ_x);                                                         clear new_x new_y                                                                     for i = 1: floor(diff)+1 % REVISED                                                                         new_x(i) = max(CQ_x) + i ;                                                                         new_y(i) = CQ_y(1,length(CQ_y)) * exp(k * i);                                                                     end                                                         CQ_x = cat(2,CQ_x,new_x);                                                         CQ_y = cat(2,CQ_y,new_y);                                                         %% Define x_C_cq                                                                                                                    num_of_daily_samples = 24; % Sample hourly                                                            initval = 0;                                                            x_C_cq = initval:1/num_of_daily_samples:(end_tm_cq);                                                                                                                     %% Interpolating Plasma Concetrations                                                                        for ctr = 1:1                                                                        CQ_interp(ctr,:) = interp1(CQ_x,log(CQ_y(ctr,:)),x_C_cq,'linear');                                                                     end                                                                     CQ_interp = exp(CQ_interp);                                                                                                                             %% Calculating daily drug levels                                                                     for ctr = 1:1                                                                        for i = 1:treatmentcourse_prtnrdrug+1                                                                                                        conc_mn_CQ(ctr,i) = mean(CQ_interp(ctr,find(((0 + (i-1)) <= x_C_cq) .* (x_C_cq <= (1 + (i-1)) ) ==  1)));                                                                        end                                                                      end                                 k_1_2_1 = 60;                            k_1_2_2 = 290;                            break_point = 7;                                                             mdCQ_y(1)= 10^-5;                                                            % mdCQ_y(2)= 10^-5;                                                            mdCQ_x = hr_mdCQ./24;                                                            mdCQ_y = conc_mdCQ;                                                         %% Stages                                                         mdCQ_x_first_stage = mdCQ_x(1:5)';                                                        mdCQ_y_first_stage = mdCQ_y(1:5)';                                                         mdCQ_y(:,1) = mdCQ_y(:,1)' .* rand_mult_Cmax; % Multiplication by the Cmax constant %                                                           %% Dilation Effect                                                         mdCQ_x = mdCQ_x_first_stage .* rand_mult_tmax; % in days                                                         %% Extrapolating Plasma Concetrations, inital clearance                                                         k = log(1/2)/(k_1_2_1/24) * rand_mult_t_clear_init; %If we want an hourly rate, we can take hourly factor directly                                                         end_tm_cq = break_point;                                                         diff = end_tm_cq-max(mdCQ_x);                                                         clear new_x new_y                                                                     for i = 1: floor(diff) % no +1 here                                                                         new_x(i) = max(mdCQ_x) + i ;                                                                         new_y(i) = mdCQ_y(5,:) * exp(k * i);                                                                     end                                                         mdCQ_x = cat(2,mdCQ_x,new_x);                                                         mdCQ_y = cat(2,mdCQ_y(1:5,1)',new_y);                                                         %% Extrapolating Plasma Concetrations, terminal clearance                                                         k = log(1/2)/(k_1_2_2/24) * rand_mult_t_clear_term; %If we want an hourly rate, we can take hourly factor directly                                                         end_tm_cq = 102;                                                         diff = end_tm_cq-max(mdCQ_x);                                                         clear new_x new_y                                                                     for i = 1: floor(diff)+1 % REVISED                                                                         new_x(i) = max(mdCQ_x) + i ;                                                                         new_y(i) = mdCQ_y(1,length(mdCQ_y)) * exp(k * i);                                                                     end                                                         mdCQ_x = cat(2,mdCQ_x,new_x);                                                         mdCQ_y = cat(2,mdCQ_y,new_y);                                                         %% Define x_C_cq                                                                                                                    num_of_daily_samples = 24; % Sample hourly                                                            initval = 0;                                                            x_C_cq = initval:1/num_of_daily_samples:(end_tm_cq);                                                                                                                     %% Interpolating Plasma Concetrations                                                                     for ctr = 1:1                                                                        mdCQ_interp(ctr,:) = interp1(mdCQ_x,log(mdCQ_y(ctr,:)),x_C_cq,'linear');                                                                     end                                                                     mdCQ_interp = exp(mdCQ_interp);                                                          %% Calculating daily drug levels                                                                     for ctr = 1:1                                                                        for i = 1:treatmentcourse_prtnrdrug+1                                                                                                        conc_mn_mdCQ(ctr,i) = mean(mdCQ_interp(ctr,find(((0 + (i-1)) <= x_C_cq) .* (x_C_cq <= (1 + (i-1)) ) ==  1)));                                                                        end                                                                      end                                  total_conc_CQ(j,:) = CQ_interp(1,:);                             total_conc_mdCQ(j,:) = mdCQ_interp(1,:);                                                         if rand_PK == 1                                Cdaily_drug(j,:) = conc_mn_CQ + conc_mn_mdCQ;                            end                                                        if random_pretreat == 1                                Cdaily_drug_pretreat(j,:) = conc_mn_CQ + conc_mn_mdCQ;                            end                                                            end % end of the individual loop                             total_conc = total_conc_CQ + total_conc_mdCQ;                               %% Convert from child nM to adult ng/ml                            if rand_PK == 1                                Cdaily_drug = Cdaily_drug .* CQ_conv_fact.*adult_to_child;                            end                                                        if random_pretreat == 1                                Cdaily_drug_pretreat = Cdaily_drug_pretreat .* CQ_conv_fact.*adult_to_child;                            end                            total_conc = total_conc .* CQ_conv_fact.*adult_to_child;                                    end % end of CQ loop             % MFQ_fixed         % Notation:        % MEF_interp --> total_conc        % x_C_mef        % conc_mn_MEF --> Cdaily_drug                 if strcmp(drug,'MEF') && strcmp(MEF_type,'fixed')                        for j = 1:numofruns_PKPD                                                          load MEF_PK_fixed.mat                                      %DELETE!!!!!!!!!!!                                                        MEF_y(1)= 10^-5;                                                        % MEF_y(2)= 10^-5;                                                        % mfq_cnst = 20;                                             %DELETE!!!!!!!!!!!                                                        MEF_x = MEF_x'./24; % day 28!                                                        MEF_y_hldr = MEF_y;                                                        % MEF_y = zeros(mfq_cnst,numofruns_PKPD);                                             %% Stages                                                         % MEF_x_first_stage = MEF_x(1:15)'./24; <-- Hmmm...                                                        MEF_x_first_stage = MEF_x(1:15)';                                                        MEF_y_first_stage = MEF_y(1:15);                                             %% Calculate stochastic constants                                             % 2 - 1/2: fails symmetry test (geomean and mean: geomean([1.5 1/1.5]) geomean([mean([1 1.5]) mean([1 1/1.5])]))                                            % 2 - 0: passes mean test, fails geomean, fails for greater than 2                                            % 2 - 1/2: skewed                                             % Factor 1: Tmax shift                                             if rand_PK == 1                                                    upper_lim = 1.55;                                                    lower_lim = 1/1.55;                                                     rand_mult = random('unif',1,upper_lim);     %Upper plasma levels raised by factor of 3                                                    rand_mult_1 = random('unif',lower_lim,1);   %Lower plasma levels decreased by factor of 9                                                      rand_mult_2 = random('unif',0,1);                                                     if rand_mult_2 < 1/(number_of_rpts(upper_lim,lower_lim)+1)                                                        rand_mult_tmax = rand_mult;                                                    end                                                     if rand_mult_2 >= 1/(number_of_rpts(upper_lim,lower_lim)+1)                                                        rand_mult_tmax = rand_mult_1;                                                                                end                                            else                                                    rand_mult_tmax = 1; % non-stochastic                                            end                                                    rand_mult_holder_tmax(j) = rand_mult_tmax;                                             % Factor 2: Cmax shift                                                     upper_lim = 1.9;                                                    lower_lim = 1/1.9;                                                     for ctr = 1:1                                                                                                                 if rand_PK == 1                                                                 rand_mult = random('unif',1,upper_lim);     %Upper plasma levels raised by factor of 3                                                                                           rand_mult_1 = random('unif',lower_lim,1);   %Lower plasma levels decreased by factor of 9                                                                                             rand_mult_2 = random('unif',0,1);                                                                                                                                  if rand_mult_2 < 1/(number_of_rpts(upper_lim,lower_lim)+1)                                                                                                rand_mult_Cmax = rand_mult;                                                                                           end                                                                                                                                    if rand_mult_2 >= 1/(number_of_rpts(upper_lim,lower_lim)+1)                                                                                                   rand_mult_Cmax = rand_mult_1;                                                                                            end                                                         else                                                                rand_mult_Cmax = 1; % non-stochastic                                                        end                                                                                                                MEF_y(ctr,:) = MEF_y_hldr .* rand_mult_Cmax;                                                                                                                 end                                                              rand_mult_holder_Cmax(j) = rand_mult_Cmax;                                             % Factor 3: t1/2 shift                                             if rand_PK == 1                                                                                                    upper_lim = 1.7;                                                    lower_lim = 1/1.7;                                                     rand_mult = random('unif',1,upper_lim);     %Upper plasma levels raised by factor of 3                                                    rand_mult_1 = random('unif',lower_lim,1);   %Lower plasma levels decreased by factor of 9                                                      rand_mult_2 = random('unif',0,1);                                                     if rand_mult_2 < 1/(number_of_rpts(upper_lim,lower_lim)+1)                                                        rand_mult_t_clear_init = rand_mult;                                                    end                                                     if rand_mult_2 >= 1/(number_of_rpts(upper_lim,lower_lim)+1)                                                        rand_mult_t_clear_init = rand_mult_1;                                                                                end                                            else                                                    rand_mult_t_clear_init = 1; % non-stochastic                                            end                                                    rand_mult_holder_t_clear_init(j) = rand_mult_t_clear_init;                                             % Factor 4: t1/2 terminal shift                                             if rand_PK == 1                                                                                                    upper_lim = 1.9;                                                    lower_lim = 1/1.9;                                                     rand_mult = random('unif',1,upper_lim);     %Upper plasma levels raised by factor of 3                                                    rand_mult_1 = random('unif',lower_lim,1);   %Lower plasma levels decreased by factor of 9                                                      rand_mult_2 = random('unif',0,1);                                                     if rand_mult_2 < 1/(number_of_rpts(upper_lim,lower_lim)+1)                                                        rand_mult_t_clear_term = rand_mult;                                                    end                                                     if rand_mult_2 >= 1/(number_of_rpts(upper_lim,lower_lim)+1)                                                        rand_mult_t_clear_term = rand_mult_1;                                                                                end                                            else                                                    rand_mult_t_clear_term = 1; % non-stochastic                                            end                                                    rand_mult_holder_t_clear_term(j) = rand_mult_t_clear_term;                                              %% Dilation Effect                                             MEF_x = MEF_x_first_stage .* rand_mult_tmax;                                             %% Extrapolating Plasma Concetrations, inital clearance                                             k = log(1/2)/(k_1_2_1/24) * rand_mult_t_clear_init; %If we want an hourly rate, we can take hourly factor directly                                             end_tm_mef = break_point;                                             diff = end_tm_mef-max(MEF_x);                                             clear new_x new_y                                                         for i = 1: floor(diff)                                                             new_x(i) = max(MEF_x) + i ;                                                             new_y(i) = MEF_y(:,15) * exp(k * i);                                                         end                                             MEF_x = cat(2,MEF_x,new_x);                                             MEF_y = cat(2,MEF_y(1,1:15),new_y);                                             %% Extrapolating Plasma Concetrations, terminal clearance                                             k = log(1/2)/(k_1_2_2/24) * rand_mult_t_clear_term; %If we want an hourly rate, we can take hourly factor directly                                             end_tm_mef = 102;                                             diff = end_tm_mef-max(MEF_x);                                             clear new_x new_y                                                         for i = 1: floor(diff)                                                             new_x(i) = max(MEF_x) + i ;                                                             new_y(i) = MEF_y(1,length(MEF_y)) * exp(k * i);                                                         end                                             MEF_x = cat(2,MEF_x,new_x);                                             MEF_y = cat(2,MEF_y,new_y);                                             end_tm_mef = 100;                                             %% Interpolating Plasma Concetrations                                                         num_of_daily_samples = 24; % Sample hourly                                                        initval = 0;                                                        for ctr = 1:1                                                            MEF_interp(ctr,:) = interp1(MEF_x,log(MEF_y(ctr,:)),initval:1/num_of_daily_samples:(end_tm_mef+1),'linear');                                                         end                                                         MEF_interp = exp(MEF_interp);                                              %% Save MEF_interp, compute PD                                                 x_C_mef = initval:1/num_of_daily_samples:(end_tm_mef+1);                                              %% Calculating daily drug levels                                                         for ctr = 1:1                                                            for i = 1:treatmentcourse_prtnrdrug+1                                                                                            conc_mn_MEF(ctr,i) = mean(MEF_interp(ctr,find(((0 + (i-1)) <= x_C_mef) .* (x_C_mef <= (1 + (i-1)) ) ==  1)));                                                            end                                                          end                                             %% Sampling                                            % increments are in hours                                                 s_t = [2 4 8 24 48 72 7*24 14*24 21*24 28*24 35*24 42*24 56*24]+1;                             sampling_vals(j,:) = MEF_interp(s_t);                                                           total_conc(j,:) = MEF_interp(1,:);                                                if rand_PK == 1                            Cdaily_drug(j,:) = conc_mn_MEF;                        end                                                    if random_pretreat == 1                            Cdaily_drug_pretreat(j,:) = conc_mn_MEF;                        end                         end         end %MFQ_fixed                             % MFQ_loose         % Notation:        % MEF_interp --> total_conc        % x_C_mef        % conc_mn_MEF --> Cdaily_drug         if strcmp(drug,'MEF') && strcmp(MEF_type,'loose')                        for j = 1:numofruns_PKPD                                                          load MEF_PK_loose.mat                                      %DELETE!!!!!!!!!!!                                                        MEF_y(1)= 10^-5;                                                        MEF_y(2)= 10^-5;                                                        % mfq_cnst = 20;                                             %DELETE!!!!!!!!!!!                                                        MEF_x = MEF_x'./24; % day 28!                                                        MEF_y_hldr = MEF_y;                                                        % MEF_y = zeros(mfq_cnst,numofruns_PKPD);                                             %% Stages                                                         % MEF_x_first_stage = MEF_x(1:4)'./24; <-- Hmmm...                                                        MEF_x_first_stage = MEF_x(1:4)';                                                        MEF_y_first_stage = MEF_y(1:4);                                             %% Calculate stochastic constants                                             % 2 - 1/2: fails symmetry test (geomean and mean: geomean([1.5 1/1.5]) geomean([mean([1 1.5]) mean([1 1/1.5])]))                                            % 2 - 0: passes mean test, fails geomean, fails for greater than 2                                            % 2 - 1/2: skewed                                             % Factor 1: Tmax shift                                             if rand_PK == 1                                                                                                    upper_lim = 1.55;                                                    lower_lim = 1/1.55;                                                     rand_mult = random('unif',1,upper_lim);     %Upper plasma levels raised by factor of 3                                                    rand_mult_1 = random('unif',lower_lim,1);   %Lower plasma levels decreased by factor of 9                                                      rand_mult_2 = random('unif',0,1);                                                     if rand_mult_2 < 1/(number_of_rpts(upper_lim,lower_lim)+1)                                                        rand_mult_tmax = rand_mult;                                                    end                                                     if rand_mult_2 >= 1/(number_of_rpts(upper_lim,lower_lim)+1)                                                        rand_mult_tmax = rand_mult_1;                                                                                end                                            else                                                    rand_mult_tmax = 1; % non-stochastic                                            end                                                    rand_mult_holder_tmax(j) = rand_mult_tmax;                                             % Factor 2: Cmax shift                                                     upper_lim = 1.9;                                                    lower_lim = 1/1.9;                                                     for ctr = 1:1                                                               if rand_PK == 1                                                                                                                                  rand_mult = random('unif',1,upper_lim);     %Upper plasma levels raised by factor of 3                                                                                       rand_mult_1 = random('unif',lower_lim,1);   %Lower plasma levels decreased by factor of 9                                                                                         rand_mult_2 = random('unif',0,1);                                                                                                                                                         if rand_mult_2 < 1/(number_of_rpts(upper_lim,lower_lim)+1)                                                                                            rand_mult_Cmax = rand_mult;                                                                                       end                                                                                                                             if rand_mult_2 >= 1/(number_of_rpts(upper_lim,lower_lim)+1)                                                                                               rand_mult_Cmax = rand_mult_1;                                                                                        end                                                                                                                        else                                                                rand_mult_Cmax = 1; % non-stochastic                                                                                                                          end                                                                MEF_y(ctr,:) = MEF_y_hldr .* rand_mult_Cmax;                                                       end                                                               rand_mult_holder_Cmax(j) = rand_mult_Cmax;                                             % Factor 3: t1/2 shift                                            if rand_PK == 1                                                                                                    upper_lim = 1.7;                                                    lower_lim = 1/1.7;                                                     rand_mult = random('unif',1,upper_lim);     %Upper plasma levels raised by factor of 3                                                    rand_mult_1 = random('unif',lower_lim,1);   %Lower plasma levels decreased by factor of 9                                                      rand_mult_2 = random('unif',0,1);                                                     if rand_mult_2 < 1/(number_of_rpts(upper_lim,lower_lim)+1)                                                        rand_mult_t_clear_init = rand_mult;                                                    end                                                     if rand_mult_2 >= 1/(number_of_rpts(upper_lim,lower_lim)+1)                                                        rand_mult_t_clear_init = rand_mult_1;                                                                                end                                            else                                                    rand_mult_t_clear_init = 1; % non-stochastic                                            end                                                    rand_mult_holder_t_clear_init(j) = rand_mult_t_clear_init;                                             % Factor 4: t1/2 terminal shift                                            if rand_PK == 1                                                    upper_lim = 1.9;                                                    lower_lim = 1/1.9;                                                     rand_mult = random('unif',1,upper_lim);     %Upper plasma levels raised by factor of 3                                                    rand_mult_1 = random('unif',lower_lim,1);   %Lower plasma levels decreased by factor of 9                                                      rand_mult_2 = random('unif',0,1);                                                     if rand_mult_2 < 1/(number_of_rpts(upper_lim,lower_lim)+1)                                                        rand_mult_t_clear_term = rand_mult;                                                    end                                                     if rand_mult_2 >= 1/(number_of_rpts(upper_lim,lower_lim)+1)                                                        rand_mult_t_clear_term = rand_mult_1;                                                                                end                                            else                                                    rand_mult_t_clear_term = 1; % non-stochastic                                            end                                                    rand_mult_holder_t_clear_term(j) = rand_mult_t_clear_term;                                             %% Dilation Effect                                             MEF_x = MEF_x_first_stage .* rand_mult_tmax;                                             %% Extrapolating Plasma Concetrations, inital clearance                                             k = log(1/2)/(k_1_2_1/24) * rand_mult_t_clear_init; %If we want an hourly rate, we can take hourly factor directly                                             end_tm_mef = break_point;                                             diff = end_tm_mef-max(MEF_x);                                             clear new_x new_y                                                         for i = 1: floor(diff)                                                             new_x(i) = max(MEF_x) + i ;                                                             new_y(i) = MEF_y(:,4) * exp(k * i);                                                         end                                             MEF_x = cat(2,MEF_x,new_x);                                             MEF_y = cat(2,MEF_y(1,1:4),new_y);                                             %% Extrapolating Plasma Concetrations, terminal clearance                                             k = log(1/2)/(k_1_2_2/24) * rand_mult_t_clear_term; %If we want an hourly rate, we can take hourly factor directly                                             end_tm_mef = 102;                                             diff = end_tm_mef-max(MEF_x);                                             clear new_x new_y                                                         for i = 1: floor(diff)                                                             new_x(i) = max(MEF_x) + i ;                                                             new_y(i) = MEF_y(1,length(MEF_y)) * exp(k * i);                                                         end                                             MEF_x = cat(2,MEF_x,new_x);                                             MEF_y = cat(2,MEF_y,new_y);                                             end_tm_mef = 100;                                             %% Interpolating Plasma Concetrations                                                         num_of_daily_samples = 24; % Sample hourly                                                        initval = 0;                                                        for ctr = 1:1                                                            MEF_interp(ctr,:) = interp1(MEF_x,log(MEF_y(ctr,:)),initval:1/num_of_daily_samples:(end_tm_mef+1),'linear');                                                         end                                                         MEF_interp = exp(MEF_interp);                                              %% Save MEF_interp, compute PD                                                 x_C_mef = initval:1/num_of_daily_samples:(end_tm_mef+1);                                              %% Calculating daily drug levels                                                         for ctr = 1:1                                                            for i = 1:treatmentcourse_prtnrdrug+1                                                                                            conc_mn_MEF(ctr,i) = mean(MEF_interp(ctr,find(((0 + (i-1)) <= x_C_mef) .* (x_C_mef <= (1 + (i-1)) ) ==  1)));                                                            end                                                          end                                             %% Sampling                                            % increments are in hours                                                 s_t = [2 4 8 24 48 72 7*24 14*24 21*24 28*24 35*24 42*24 56*24]+1;                            sampling_vals(j,:) = MEF_interp(s_t);                        total_conc(j,:) = MEF_interp(1,:);                                                 if rand_PK == 1                            Cdaily_drug(j,:) = conc_mn_MEF;                        end                                                    if random_pretreat == 1                            Cdaily_drug_pretreat(j,:) = conc_mn_MEF;                        end                         end         end %MFQ_loose     end       %% PD --> 0 - 100     g1 = @(x) ((0-1)./(1+((x./p_model(2)).^p_model(1)))) + 1 ; %This function takes drug plasma concentrations in ng/ml!!!        PD_drug = g1(total_conc);       if probtreat == 0;        numofruns_PKPD = 1;    end         for j = 1:numofruns_PKPD                if strcmp(drug,'LMF')                         for i = 1:treatmentcourse_prtnrdrug+1 % right endpoints                                mn_PD(j,i) = mean(PD_drug(j, (find(((0 + 24*(i-1)) <= x_C_lum/upd_incrmnt) .* (x_C_lum/upd_incrmnt <= (24 + 24*(i-1)) ) ==  1)) ) );                            end                                end         if strcmp(drug,'CQ')                         for i = 1:treatmentcourse_prtnrdrug+1 % right endpoints                                mn_PD(j,i) = mean(PD_drug(j, (find(((0 + (i-1)) <= x_C_cq) .* (x_C_cq <= (1 + (i-1)) ) ==  1)) ) );                            end                                end         if strcmp(drug,'MEF') && strcmp(MEF_type,'fixed')                         for i = 1:treatmentcourse_prtnrdrug+1 % right endpoints                                mn_PD(j,i) = mean(PD_drug(j, (find(((0 + (i-1)) <= x_C_mef) .* (x_C_mef <= (1 + (i-1)) ) ==  1)) ) );                            end                                end                if strcmp(drug,'MEF') && strcmp(MEF_type,'loose')                         for i = 1:treatmentcourse_prtnrdrug+1 % right endpoints                                mn_PD(j,i) = mean(PD_drug(j, (find(((0 + (i-1)) <= x_C_mef) .* (x_C_mef <= (1 + (i-1)) ) ==  1)) ) );                            end                                end            end        %% User-Defined Constants     %{    if isfloat(input_1)        numofruns = input_1;    end         if isfloat(input_2)        probtreat = input_2;    end     if isfloat(input_3)        treatmentwait = input_3;    end     if ischar(input_4)        drug = input_4;    end     if ischar(input_5)        MEF_type = input_5;    end     if isfloat(input_6)        RRfstdrug = -log10(input_6);    end     if isfloat(input_7)        RRsnddrug = -log10(input_7);    end        if isfloat(input_8)        p_model(2) = input_8;    end        if isfloat(input_9)        rand_PK = input_9;    end     if isfloat(input_10)        pretreated = input_10;    end        if isfloat(input_11)        Adjalley = input_11;    end        if isfloat(input_12)        trans_block = input_12;    end        if isfloat(input_13)        shift = input_13;    end    %}    %% Fixed Constants     %numoferrors = 1;     initval = 1;    tstep = 2;    endtm = 801;                                     %This value must be odd    factor = ones(1,endtm);                          %Default Values    s = .02;                                         %.02    v = 50;                                          %50        kc = .2*.82;                                 %.2                   Pvstar = 30;                                     %30    deltav = 8;                                      %8        sigma = 0.1500;                              %.02 *consider .2*    beta = .01;                                      %.01    C = 1;                                           %1        %km = .0250;                                 %.04    deltam = 8;                                      %8                         rho = 0;                                         %0    mum = 16;                                        %16    stdev = 10.4;                                    %10.4    q = .3;                                          %.3    kapc = 3;                                        %3    kapv = 3;                                        %3    kapm = 1;                                        %1    minparasitemia = 10^-5;                          %10^-5    fevconst = .0002;    mu_a = 10^(4.79);         Geomean = 0;    varmax = 1.14766;                                %1.14766    gompcst1 = .0311;    gompcst2 = .0004;    % gompcst1 = .0085;    % gompcst2 = .0012;    detectionthresh = 10;    checker1 = 0;    checker2 = 0;    checker3 = 0;    firstobs = zeros(1,numofruns);                   %first observation day    tstar = zeros(1,numofruns);                      %last observation day    %Poissonthresh = 100;    tinterp = 1;     %%%%%%    twalk = 1;    %%%%%%     begtm = 1;    lengthtm = (endtm-begtm)*(1/twalk) + 1;    mingametocytemia =  10^-5;    %Poissonthresh_gam = 100;           %Values for the Poisson error calculation    tinterp = 1;    %numoferrors_gam = 1;    maxgamma = .135 * 1.4;              %Conversion factor was necessary only for data analysis, not theoretical values    minpeak = 100;                      mu = 7;    a = 1;    b = 33;    sig = 1.5;    PHIl = normcdf((a-mu)/sig);    PHIr = normcdf((b-mu)/sig);    logn1 = -6;                         % logn1 = -6.2898;     logn2 = 4;                          % logn2 = 3.7272;    beta_gam = .0029 * (1/log(10));    muzero = .03;    alphaGconst = 2;     %%%%%%     mintrans = 2/3;    feverduration = 0;                  fevernumber = 5;    feverperiodicity = 2;    mininfectivity = 1.0 * 10^-5;    maxinfectivity = 1.0; %% Drug pretreatment     Cdaily_drug_shift = zeros(1,endtm);        % Deterministic pretreatment drug levels         if pretreated == 1             if random_pretreat == 0                %% Generate shifted partner drug concentrations                for i = 1:treatmentcourse_prtnrdrug-shift                     Cdaily_drug_shift(i) = Cdaily_drug_standard(i+shift);                    end            end        end     %% Determine effect of parter drug treatment on gametocytemia death rates      fstboostconstant = 1;    sndboostconstant = 1;      gam_killing_treat = zeros(numofruns,endtm);    gam_killing_shift = zeros(1,endtm);    %% Prefetching memory     Ptot = zeros(numofruns,endtm);      peaks = zeros(numofruns,1);    Ptotstar = zeros(numofruns,1);    Pmstar = zeros(numofruns,1);    Pinterp = zeros(numofruns,endtm);    %Ptotpoisson = zeros(numoferrors,numofruns,endtm);    %Pinterppoisson = zeros(numoferrors,numofruns,endtm);    Pfstar = zeros(1, numofruns);    feverday = zeros(1, numofruns);    oldfeverday = zeros(1, numofruns);      posday = zeros(1, numofruns);   %This is the first day with 0 asexual parasitemia    Para_fevday = zeros(1, numofruns);      m = zeros(numofruns,v);                 km = zeros(numofruns,1);                %%%%%%    treatparasit = zeros(numofruns,endtm);    fstdruglevels = zeros(numofruns,endtm);    prtnrdruglevels = zeros(numofruns,endtm);    randval = zeros(1,numofruns);    treated_asexual = zeros(1,numofruns);    mod_real_P = zeros(numofruns,endtm);    mod_real_DHA = zeros(numofruns,endtm);    total_real_P = zeros(numofruns,endtm);    total_real_DHA = zeros(numofruns,endtm);    repeatcounter = zeros(1,numofruns);    %%%%%%    G = zeros(numofruns,lengthtm);         gamma = zeros(numofruns,1);         delay = zeros(1,numofruns);    Astar = zeros(1,numofruns);    trueAstar = zeros(1,numofruns);    Gstar = zeros(1,numofruns);    trueGstar = zeros(1,numofruns);    gprod = zeros(1,numofruns);    gbar = zeros(1,numofruns);    truegbar = zeros(1,numofruns);    L = zeros(1,numofruns);    trueL = zeros(1,numofruns);    %Gpoisson = zeros(numoferrors,numofruns,endtm);    endconstant = zeros(1,numofruns);    treated_gam = zeros(1,numofruns);    gamobsday = zeros(1,numofruns);    alphaG = zeros(numofruns,endtm);    %%%%%%    c = zeros(numofruns,endtm);    feverset = zeros(numofruns,(fevernumber*(feverduration+1)));    initval_treat = ones(numofruns,1);     % progressbar('Asexual Calculations')     %% Thailand values        % a_thai = .5;                                                                          % THAILAND    % b_thai  = 18.5;                                                                       % THAILAND    % mu_thai  = 8;                                                                         % THAILAND    % sigma_thai  = 4;                                                                      % THAILAND     % PHIl_thai = normcdf((a_thai-mu_thai)/sigma_thai);    % PHIr_thai = normcdf((b_thai-mu_thai)/sigma_thai);     % thresh = round(mu_thai + sigma_thai*( sqrt(2)*erfinv(2*(PHIl_thai+(PHIr_thai-PHIl_thai)*rand(1,numofruns))-1) )); %% Asexual Calculations         for d = 1:numofruns;             % Stochastic pretreatment drug levels                 if pretreated == 1                     if random_pretreat == 1 % the existence of Cdaily_drug_pretreat allows for random_pretreat == 1 and rand_PK == 0                        %% Generate shifted partner drug concentrations                        for i = 1:treatmentcourse_prtnrdrug-shift                             Cdaily_drug_shift(i) = Cdaily_drug_pretreat(d,i+shift); % Will replace with more compliated algorithm if necessary                           end                    end                end             % Shifted Partner Drug Asexuals PD            real_P_shift = zeros(1,endtm);            total_real_P_shft = zeros(1,endtm);             real_P_shift(1:treatmentcourse_prtnrdrug) = g1(Cdaily_drug_shift(1:treatmentcourse_prtnrdrug));             RR_ctr = 1;            for i = initval:twalk:endtm                                              if ismember(RR_ctr,initval:tstep:endtm) && (i < endtm)                                                total_real_P_shft(i) = mean([real_P_shift(i) real_P_shift(i+1)]); % earlier concentration begins on day 1                             end            RR_ctr = RR_ctr + 1;            end            RR_ctr = 1;            % if random('unif',0,1) <= (3/12)                                                   % THAILAND        %   treatmentoverride = [1 15];     %Overrides feverday treatment and substitutes given start day for all individuals        % else        %   treatmentoverride = [1 16];     %16        % end                %% Prefetching memory        checker1 = 0;        checker2 = 0;        checker3 = 0;        P = zeros(v,endtm);             %Parasitemias of all of the variants        Pprime = zeros(v,endtm);        S = zeros(v,endtm);        Phatc = zeros(1,endtm);        Sc = zeros(1,endtm);        Pinternal = zeros(v,endtm);        Sm = zeros(1,endtm);        p = zeros(v,endtm);        Phtint = zeros(1,endtm);        Sum = 0;        holder = 0;        posdayctr = 0;        new_ctr = 1;         %*******************************************************************        P(1,1) = .1;         %*******************************************************************         %% Random Constants           km(d) = 0.0250;         Ptotstar(d) = kc * exp( Geomean + varmax * randn )*mu_a;                                        while log10( (1/kc) *Ptotstar(d)) >= 5.5 % --> ~10% max model parasitemia            % 5.66 was the maximum observed!!!            % 5.7782 = 10% parasitemia for a high Hematocrit male --> ~16% max model parasitemia            Ptotstar(d) = kc * exp( Geomean + varmax * randn )*mu_a;          end         Pmstar(d) = km(d) *( (log( 1 - log(1-rand(1))*gompcst1/gompcst2 ) / gompcst1));                                                                        %% Defining the phenotypic-inherent growth rates (censored normal distribution)        for x=1:v                  m(d,x) = mum + stdev*randn;                     if m(d,x)>=35                    m(d,x) = 35;                   end                    if m(d,x)<1                    m(d,x)=1;                end                       end    %% Start of the asexuals calculation loop        while repeatcounter(d) < 2;             for i = (initval_treat(d)):tstep:endtm              %% Total parasites at the time step i: NON-STOCHASTIC                Ptot(d,i)= sum(P(:,i));                        %% I) Innate immune response: NON-STOCHASTIC                  Sc(i) = (1 + ( (1/Ptotstar(d)) * Ptot(d,i))^kapc )^-1;                                %% II) Acquired variant-specific immune response: NON-STOCHASTIC                if i - deltav > 0                                              for x = 1:v                                            for k = initval:tstep:i-deltav                                                               holder = P(x,k)*exp(-sigma * (i - k - deltav) ) + holder;                                                                   end                                           Pinternal(x,i) = holder;                                                              holder = 0;                                             end                                        end                      for x = 1:v                                                      S(x,i) = (1 + ( (1/Pvstar) * Pinternal(x,i))^kapv  )^-1;                                             end                    %clear x                          %% III) Acquired variant-transcending immune response: NON-STOCHASTIC                if Ptot(d,i) < C,                    Phatc(i) = Ptot(d,i);                                        else                    Phatc(i) = C;                end                 if i - deltam > 0                    for k = initval:tstep:i-deltam                         holder = Phatc(k)*exp(-rho * (i - k - deltam) ) + holder;                                                                            end                                                    Phtint(i) = holder;                                             holder = 0;                                          end                                    Sm(i) = (1-beta)*(1 + ((1/Pmstar(d)) * Phtint(i))^kapm  )^-1 + beta;     %% Defining the switching rates: NON-STOCHASTIC                              for x = 1:v                                        if S(x,i) < .1                                                 p(x,i)= 0;                             else                                                for ind = 1:v                            Sum = Sum + (q^ind)*S(ind,i);                        end                                                                   p(x,i) = (q^x)*S(x,i)/Sum;                    Sum = 0;                    end                                                end                %clear x    %% Grow Parasites in Presence of Immune Response & Drug Response: NON-STOCHASTIC                 if i<=endtm-tstep,                    for x = 1:v                                             Pprime(x,i+tstep) =  10^( log10 (( (1-s)*P(x,i)+s*p(x,i)*Ptot(d,i) )*m(d,x)*Sc(i)*S(x,i)*Sm(i)) + total_real_P(d,i) * RRsnddrug + total_real_DHA(d,i) * RRfstdrug + total_real_P_shft(i) * RRsnddrug);                                               end                              end    %% Kill off low levels of parasitemia: NON-STOCHASTIC                 if i<=endtm-tstep,                        for x = 1:v                                            if Pprime(x,i+tstep) >= minparasitemia,                                                    P(x,i+tstep) = Pprime(x,i+tstep);                                                else                                                   P(x,i+tstep) = minasexualval;                                                                       end                    end                                    %clear x                end            end %This is the end of the initval_treat(d) time loop         %% Interpolating Asexual Parasitemia: NON-STOCHASTIC         Pinterp(d,:) = interp1(initval:tstep:length(Ptot(d,:)),log10(Ptot(d,initval:tstep:length(Ptot(d,:)))),initval:tinterp:endtm,'linear');        Pinterp(d,:) = 10.^Pinterp(d,:);        for i = 1:endtm             if isnan(Pinterp(d,i))==1;                 Pinterp(d,i) = 0;             end        end         %% Finding first detection day: NON-STOCHASTIC         for i = initval:tstep:endtm                     if checker1 == 0                    if Pinterp(d,i) >= detectionthresh,                     firstobs(d) = i;                    checker1 = 1;                        end                     end            end        %% Computing the fever threshold and finding the last positive day of asexual parasitemia: STOCHASTIC                 Pfstar(d) = ( 10^(log10(fevconst) + (0-log10(fevconst))*rand) ) * max(Ptot(d,:))  ;        for i = initval:tinterp:endtm               if checker3 == 0                   if Pinterp(d,i) >= Pfstar(d)                                   feverday(d) = i;                     checker3 = 1;                        end               end                if posdayctr == 0                    if Pinterp(d,i) == 0 || i == endtm                                   posday(d) = i;                     posdayctr = 1;                        end                end            end                %% Apply the treatmentoverride/stochastic_start, if necessary                if repeatcounter(d) == 0            oldfeverday(d) = feverday(d);        end                treatmentoverride = [1 0];                                                          % THAILAND                if (treatmentoverride(1) == 1  && repeatcounter(d) == 0 && stochastic_start ~= 1)                         treatmentoverride = [1 oldfeverday(d) + 5];                                     % THAILAND            feverday(d) = treatmentoverride(2);        end                    if (stochastic_start == 1 && repeatcounter(d) == 0 && treatmentoverride(1) ~= 1)                feverday(d) = oldfeverday(d) + round(random('wbl',params(1),params(2),1)); %a/k - shape; b/theta - scale        end                %% Record parasitemia on Fever Day, before drug effects                if repeatcounter(d) == 0            Para_fevday(d) = Pinterp(d,feverday(d));         end         %% Calculate Treated Drug Levels                randval(d) = rand;                %randval(d) = random('unif',0,1);         if (randval(d) <= probtreat) && (repeatcounter(d) == 0)                 treated_asexual(d) = 1;               drug_ctr = 1;                             %% Calculate plasma levels and PD effects of partner drug                    for i = initval:twalk:endtm                         % DHA-equivalent effects                            if i >= (feverday(d) + treatmentwait) && i < (feverday(d) + treatmentwait + treatmentcourse_fstdg)                                                 fstdruglevels(d,i) = 1;                                mod_real_DHA(d,i) = COMB_effect_daily(drug_ctr);                                                    else                                                        fstdruglevels(d,i) = 0;                                mod_real_DHA(d,i) = 0;                                                    end                                                %Partner drug effects                            if i >= (feverday(d) + treatmentwait) && i < (feverday(d) + treatmentwait + treatmentcourse_prtnrdrug)                                                                                 prtnrdruglevels(d,i) = Cdaily_drug(d,drug_ctr); % Calculates partner drug levels based on time of treatment                                mod_real_P(d,i) = mn_PD(d,drug_ctr);            % Calculates partner drug PD effects on asexuals                                                      drug_ctr = drug_ctr + 1;                                                                                                      if i >= (feverday(d) + treatmentwait) && i < (feverday(d) + treatmentwait) + full_time                                          mod_real_P(d,i) = 1;                    % If partner has full killing (full time), set mod_real_P = 1                                    end                            else                                                       prtnrdruglevels(d,i) = 0;                                mod_real_P(d,i) = 0;                                                end                          end % End of the for time loop                 %% Calculate daily reduction ratios, after interpolation                %Asexual drug effects to operate on the same day as drug uptake                         %Partner drug effects                        RR_ctr = 1;                         for i = initval:twalk:endtm                                                         if ismember(RR_ctr,initval:tstep:endtm) && (i < endtm)                                                            total_real_P(d,i) = mean([mod_real_P(d,i) mod_real_P(d,i+1)]);                                        end                            RR_ctr = RR_ctr + 1;                        end                         % DHA-equivalent effects                        RR_ctr = 1;                        for i = initval:twalk:endtm                                                         if ismember(RR_ctr,initval:tstep:endtm) && (i < endtm)                                                            total_real_DHA(d,i) = mean([mod_real_DHA(d,i) mod_real_DHA(d,i+1)]);                                         end                            RR_ctr = RR_ctr + 1;                        end                        RR_ctr = 1;                 %% Calculate new initial value to start asexuals loop                            for i = initval:twalk:endtm                            if ((total_real_P(d,i) ~= 0) || (total_real_DHA(d,i) ~= 0)) && (initval_treat(d) == 1)                                                       initval_treat(d) = (i - tstep);                                                    if initval_treat(d) == -1                                    initval_treat(d) = 1;                                end                                                    end                        end             repeatcounter(d) = repeatcounter(d) + 1;            else                   repeatcounter(d) = 2;           end %End of the if treatment statement         end %This is the end of the while loop     %% Finding last detection day     checker3 = endtm;        while tstar(d) == 0 && checker3 > 1;                    if Pinterp(d,checker3)>=detectionthresh                 tstar(d) = checker3;            end            checker3 = checker3 - twalk;        end     %% Report asexual treatment completion    d    % progressbar(d/numofruns)     end %This is the end of the d = number of individuals loop %% Calculate the Total Duration of Infection % tstar = tstar - firstobs;    %% Introduction of Poisson Errors (see Molineaux, et al., 2001, pg. 383)    %{    for z  = numoferrors;      for d = 1:numofruns            for i = initval:tstep:endtm               if Ptot(d,i) <= Poissonthresh                           Ptotpoisson(z,d,i) = random('poiss',Ptot(d,i),1);                          else                        Ptotpoisson(z,d,i) = random('poiss',Poissonthresh,1)*Ptot(d,i);                        end                      end     Pinterppoisson(z,d,:) = interp1(initval:tstep:length(Ptotpoisson(z,d,:)), log10( reshape(Ptotpoisson(z,d,initval:tstep:length(Ptotpoisson(z,d,:))), 1, []) ) ,initval:tinterp:endtm,'linear');    Pinterppoisson(z,d,:) = 10.^Pinterppoisson(z,d,:);    Pinterppoisson(z,d,:) = interp1(initval:tstep:length(Ptotpoisson(z,d,:)), reshape(Ptotpoisson(z,d,initval:tstep:length(Ptotpoisson(z,d,:))), 1, []) ,initval:tinterp:endtm,'linear');    end    end    %}     % Treated and shifted drug levels have been calculated %% Calculate Direct Gametocyte Killing Properties    % Cdaily_drug_shift <-- shifted contribution% prtnrdruglevels <-- treatment contribution         for j = 1:endtm            if Cdaily_drug_shift(1,j) > adjalley_5x_const;                gam_killing_shift(1,j) = j; % CORRECTED            end        end         gam_killing_shift = gam_killing_shift(gam_killing_shift~=0); % CORRECTED         for i = 1:numofruns             for j = 1:endtm                if prtnrdruglevels(i,j) > adjalley_5x_const;                    gam_killing_treat(i,j) = j; % CORRECTED                end            end           end         if pretreated == 0            prtnrdrug_set = gam_killing_treat; % CORRECTED        else            prtnrdrug_set_holder = [];            prtnrdrug_set = cat(2,gam_killing_treat,ones(numofruns,1)*gam_killing_shift); %CORRECTED!!!            for d = 1:numofruns                prtnrdrug_set_holder(d,:) = cat(2,unique(prtnrdrug_set(d,:)),zeros(1,endtm-length(unique(prtnrdrug_set(d,:))))); % CORRECTED            end            prtnrdrug_set = prtnrdrug_set_holder;        end        %% Calculate Gametocyte Inhibition Time     selection_set = zeros(numofruns,endtm); %% Gametocytemia with Drug Effects     factor = ones(numofruns,endtm);     factor_DHA = ones(1,endtm);    factor_prtnrdrug = ones(1,endtm);    factor_thrd_drug = ones(1,endtm);                                                                       % GAMETOCYTOCIDAL DRUG        if Adjalley == 0                      factor_DHA(1:13) = DHA_invitro_fact.*[.2 .2 .2 .2 .25 .25 .25 .25 0.8 0.8 0.8 0.8 0.8];             factor_DHA(14:endtm) = factor_DHA(13);         if strcmp(drug,'CQ')             factor_DHA = ones(1,endtm);        end                if strcmp(drug,'LMF')             factor_prtnrdrug(1:13) = prtnr_invitro_fact.*[.25 .25 .25 .25 1 1 1 1 1 1 1 1 1];        end        if strcmp(drug,'MEF')            factor_prtnrdrug(1:13) = prtnr_invitro_fact.*[.25 .25 .25 .25 1 1 1 1 1 1 1 1 1];        end        if strcmp(drug,'CQ')            factor_prtnrdrug(1:13) = prtnr_invitro_fact.*[.25 .25 .25 .25 1 1 1 1 1 1 1 1 1];        end                factor_prtnrdrug(14:endtm) = factor_prtnrdrug(13);     end        %% Apply the proportional loss constants from Adjalley et al.    % No delay/Delay        if Adjalley ~= 0              %factor_DHA(1:13) = DHA_invitro_fact.*[fst_stage_cst*0.547 fst_stage_cst*0.547 fst_stage_cst*0.547 fst_stage_cst*0.547 fst_stage_cst*0.826 fst_stage_cst*0.826 fst_stage_cst*0.826 snd_stage_cst*0.812 snd_stage_cst*0.812 snd_stage_cst*0.812 snd_stage_cst*0.831 snd_stage_cst*0.831 snd_stage_cst*0.831];        %factor_DHA(14:endtm) = snd_stage_cst*DHA_invitro_fact.*0.831;                factor_DHA(1:13) = DHA_invitro_fact.*[fst_stage_cst*0.547 fst_stage_cst*0.547 fst_stage_cst*0.547 fst_stage_cst*0.547 fst_stage_cst*0.826 fst_stage_cst*0.826 fst_stage_cst*0.826 snd_stage_cst* 1 snd_stage_cst* 1 snd_stage_cst* 1 snd_stage_cst*1 snd_stage_cst*1 snd_stage_cst*1];        factor_DHA(14:endtm) = 1; % snd_stage_cst*DHA_invitro_fact.*0.831;                if strcmp(drug,'CQ')             factor_DHA = ones(1,endtm);        end            %factor_prtnrdrug(1:13) = prtnr_invitro_fact.*[fst_stage_cst*GAM(Adjalley,1) fst_stage_cst*GAM(Adjalley,1) fst_stage_cst*GAM(Adjalley,1) fst_stage_cst*GAM(Adjalley,1) fst_stage_cst*GAM(Adjalley,2) fst_stage_cst*GAM(Adjalley,2) fst_stage_cst*GAM(Adjalley,2) snd_stage_cst*GAM(Adjalley,3) snd_stage_cst*GAM(Adjalley,3) snd_stage_cst*GAM(Adjalley,3) snd_stage_cst*GAM(Adjalley,4) snd_stage_cst*GAM(Adjalley,4) snd_stage_cst*GAM(Adjalley,4)];            %factor_prtnrdrug(14:endtm) = snd_stage_cst*prtnr_invitro_fact.*GAM(Adjalley,4); % = prtnr_invitro_fact.*1;  % = 1;                        factor_prtnrdrug(1:13) = prtnr_invitro_fact.*[fst_stage_cst*GAM(Adjalley,1) fst_stage_cst*GAM(Adjalley,1) fst_stage_cst*GAM(Adjalley,1) fst_stage_cst*GAM(Adjalley,1) fst_stage_cst*GAM(Adjalley,2) fst_stage_cst*GAM(Adjalley,2) fst_stage_cst*GAM(Adjalley,2) snd_stage_cst*GAM(Adjalley,3) snd_stage_cst*GAM(Adjalley,3) snd_stage_cst*GAM(Adjalley,3) snd_stage_cst*GAM(Adjalley,4) snd_stage_cst*GAM(Adjalley,4) snd_stage_cst*GAM(Adjalley,4)];            factor_prtnrdrug(14:endtm) = snd_stage_cst*prtnr_invitro_fact.*GAM(Adjalley,4); % = prtnr_invitro_fact.*1;  % = 1;             %factor_thrd_drug(1:13) = .33 .* (.75.*[.2 .2 .2 .2 .25 .25 .25 .25 0.8 0.8 0.8 0.8 0.8]);       % GAMETOCYTOCIDAL DRUG            %factor_thrd_drug(1:13) = [0.767 0.767 0.767 0.767 0.821 0.821 0.821 0.884 0.884 0.884 0.841 0.841 0.841]; %PMQ            %factor_thrd_drug(1:13) = .2.*[0.333 0.333 0.333 0.333 0.724 0.724 0.724 0.695 0.695 0.695 0.605 0.605 0.605]; %MB                         %factor_thrd_drug(1:13) = .9 .* [0.333 0.333 0.333 0.333 0.724 0.724 0.724 0.695 0.695 0.695 0.605 0.605 0.605]; %MB              %factor_thrd_drug(14:endtm) = factor_thrd_drug(13);                                              % GAMETOCYTOCIDAL DRUG                end            if trans_block == 1  % CORRECTED            factor_DHA = zeros(1,endtm);            factor_prtnrdrug = zeros(1,endtm);        end                inf_thresh = 2; %<-- define before gam loop; with set to 0, no diff btwn inf_G and G                    %IMMATURE GAMETOCYTES    inf_G = zeros(numofruns,endtm); %<-- define before gam loop                                             %IMMATURE GAMETOCYTES                % progressbar('Gametocyte Calculations')            for d = 1:numofruns;             factor_inf = zeros(1,endtm); %<-- define before gam loop                                                %IMMATURE GAMETOCYTES        %% Random constants                        holder = 0;    holder_inf = 0;                                                                                         %IMMATURE GAMETOCYTES                                                %% alphaG                         holder = .06 + (1-.06).*rand;       % holder = .013 + (1-.013).*rand;                        for i = 1:endtm                                                 alphaG(d,i) = holder;           % alphaG(d,i) = .5;   %6.3333                        end                        holder = 0; % solves the jumping problem                                                % Earlier Method for Calculating alphaG                        % DEPRECIATED                            % holder = 0;                             % holder = -log(rand)/alphaGconst;                            % while (holder < 0.039003) || (holder > 2.007) %7.5th percentile                               %     holder = -log(rand)/alphaGconst;                            % end                            % holder = 0;         %% Delay                         delay(d) = round(mu + sig*( sqrt(2)*erfinv(2*(PHIl+(PHIr-PHIl)*rand(1))-1) ));                          while (delay(d)<4) || (delay(d)>12)                            delay(d) = round(mu + sig*( sqrt(2)*erfinv(2*(PHIl+(PHIr-PHIl)*rand(1))-1) )); % delay(d) = 4 + (12-4).*rand                        end         %% Calculate Gametocyte Inhibition Time                                % DEPRECIATED                            % for i = 1:twalk:endtm                                       %   if selection_set(d,i)==1                                    %       alphaG(d,i) = holder;   %6.3333                               %   end                                            % end         %% Peaks                      counter = 1;                    for i = initval + 6*tinterp : tinterp : (length(Pinterp(d,:)) - 6*tinterp - initval)                          if Pinterp(d,i)>Pinterp(d,i - 6*tinterp) && Pinterp(d,i)>Pinterp(d,i - 5*tinterp) && Pinterp(d,i)>Pinterp(d,i - 4*tinterp)...                                && Pinterp(d,i)>Pinterp(d,i - 3*tinterp) && Pinterp(d,i)>Pinterp(d,i - 2*tinterp) && Pinterp(d,i)>Pinterp(d,i - tinterp)...                                && Pinterp(d,i)>=Pinterp(d,i+tinterp) && Pinterp(d,i)>=Pinterp(d,i+2*tinterp) && Pinterp(d,i)>=Pinterp(d,i+3*tinterp)...                                && Pinterp(d,i)>=Pinterp(d,i+4*tinterp) && Pinterp(d,i)>=Pinterp(d,i+5*tinterp) && Pinterp(d,i)>=Pinterp(d,i+6*tinterp)...                                && Pinterp(d,i)>=minpeak                         peaks(d,counter) = i;                        counter = counter + 1;                        end                    end         %% Calculate Gamma for Each Peak                   for i = 1:length(peaks(d,:))                        gammacst(i) = exp(logn1 + logn2 * randn); % fstboostconstant, sndboostconstant can be utilized here                        while gammacst(i) >= maxgamma                           gammacst(i) = exp(logn1 + logn2 * randn);                        end                end         %% Calculate Intermediate Points Between Peaks                  for i = 1:(length(peaks(d,:))-1)                    intvals(i) = (peaks(d,i+1)-peaks(d,i))/2; %These are the intermediate values that start a new peak                    intvals(i) = intvals(i) + peaks(d,i);                   end                 intvals(length(peaks(d,:))) = endtm + 1;         %% Define the Gamma Function                 counter = 1;                    for i = begtm:twalk:endtm                        if i < intvals(counter)  %We will include the nadir with the next peak, not the prior, for determination of production rate                            gamma(d,i) = gammacst(counter);                    else                            counter = counter + 1;                        gamma(d,i) = gammacst(counter);                       end                   end       %% Calculate DHA Gametocyte Effect Time                  DHA_set = ( feverday(d)+treatmentwait : feverday(d)+treatmentwait+full_DHA_day-1 );                %DHA_set changes every interation with feverday(d)                 thrd_drug_set = DHA_set + 2;                                                                % GAMETOCYTOCIDAL DRUG                %thrd_drug_set = DHA_set;                                                                     % GAMETOCYTOCIDAL DRUG          %% Calculate Gametocyte Production with Stepwise Gamma Function Assuming Treatment                 A = Pinterp(d,:); %The above calculation is unnecessary if we use the treated parasitemia values                A_hldr = Pinterp(d,:) + 1;                % A_hldr = Pinterp(d,:);                                DHA_counter = 1;                prtnrdrug_counter = 1;                thrd_drug_counter = 1;                                                                      % GAMETOCYTOCIDAL DRUG                                if (treated_asexual(d) == 1) && (gam_effect == 1);                                            treated_gam(d) = 1;                    for i = begtm:twalk:endtm                                                                  age = fliplr(((delay(d)+1):i)-delay(d)); %<-- define in gam loop                %IMMATURE GAMETOCYTES                            factor_inf(1,1:length(age)) = (age > inf_thresh) + (age < inf_thresh).*0;       %IMMATURE GAMETOCYTES                                                         if  i > delay(d) %For justification of the < as opposed to <=, see page 498, Eichner, 1943.pdf                %%  Calculate Gametocytemia: Multiplicative Effects, Staggered                                 if ismember(i,DHA_set)                                    for k = 1:(feverday(d)+treatmentwait)+DHA_counter-1                                        factor(d,feverday(d)+treatmentwait-k+DHA_counter) = factor_DHA(k)*factor(d,feverday(d)+treatmentwait-k+DHA_counter);                                                          end                                               DHA_counter = 1 + DHA_counter;                                          end                                 if ismember(i,prtnrdrug_set(d,:)) % This is the loop for the partner drug effect                                    for k = 1:(feverday(d)+treatmentwait)+prtnrdrug_counter-1                                        factor(d,feverday(d)+treatmentwait-k+prtnrdrug_counter) = factor_prtnrdrug(k)*factor(d,feverday(d)+treatmentwait-k+prtnrdrug_counter);                                                          end                                                prtnrdrug_counter = 1 + prtnrdrug_counter;                                end                                 if ismember(i,thrd_drug_set)                                                % GAMETOCYTOCIDAL DRUG                                    for k = 1:(feverday(d)+treatmentwait)+thrd_drug_counter-1                                        factor(d,feverday(d)+treatmentwait-k+thrd_drug_counter) = factor_thrd_drug(k)*factor(d,feverday(d)+treatmentwait-k+thrd_drug_counter);                                                          end                                                thrd_drug_counter = 1 + thrd_drug_counter;                              % GAMETOCYTOCIDAL DRUG                                end                                                                                for tao = (delay(d) + 1):twalk:i                                                                             holder = factor(d,tao - delay(d))*gamma(d,(tao - delay(d))) * A(tao - delay(d)) * exp(- muzero/alphaG(d,tao) *(exp (alphaG(d,tao)*(i - tao)) - 1) - beta_gam * sum(log(A_hldr(tao: twalk: i))) ) + holder;                                                                      end                                                  for tao = (delay(d) + 1):twalk:i                            %IMMATURE GAMETOCYTES                                                                            holder_inf = factor_inf(1,tao - delay(d))*factor(d,tao - delay(d))*gamma(d,(tao - delay(d))) * A(tao - delay(d)) * exp(- muzero/alphaG(d,tao) *(exp (alphaG(d,tao)*(i - tao)) - 1) - beta_gam * sum(log(A_hldr(tao: twalk: i))) ) + holder_inf; %IMMATURE GAMETOCYTES                                                                     end                                                         %IMMATURE GAMETOCYTES                                                 G(d,i) = holder;                                                                                                 holder = 0; %resets the dummy                                                      inf_G(d,i) = holder_inf;                                    %IMMATURE GAMETOCYTES                                                holder_inf = 0; %resets the dummy                           %IMMATURE GAMETOCYTES                                                                %% Kill Low Levels of Gametocytemia                                  if G(d,i) < minparasitemia                                                         G(d,i) = mingamval;                                 end                                                                  if inf_G(d,i) < minparasitemia                                            %IMMATURE GAMETOCYTES                                                        inf_G(d,i) = mingamval;                                                 %IMMATURE GAMETOCYTES                                  end                                                                       %IMMATURE GAMETOCYTES                                                                                         end %This is the end of the i > delay(d) loop                         end %This is the end of the time loop                                        else %This is the else of the randval(d) <= probval statement                %% Calculate Gametocytemia Assuming No Drug Effects                       for i = begtm:twalk:endtm                         if i > delay(d)                             for tao = (delay(d) + 1):twalk:i                                                              holder = gamma(d,(tao - delay(d))) * A(tao - delay(d)) * exp(- muzero/alphaG(d,tao) *(exp (alphaG(d,tao)*(i - tao)) - 1) - beta_gam * sum(log(A_hldr(tao: twalk: i)))) + holder;                                     end                             age = fliplr(((delay(d)+1):i)-delay(d)); %<-- define in gam loop                %IMMATURE GAMETOCYTES                            factor_inf(1,1:length(age)) = (age > inf_thresh) + (age < inf_thresh).*0;       %IMMATURE GAMETOCYTES                                                                            for tao = (delay(d) + 1):twalk:i                            %IMMATURE GAMETOCYTES                                                                       holder_inf = factor_inf(1,tao - delay(d))*factor(d,tao - delay(d))*gamma(d,(tao - delay(d))) * A(tao - delay(d)) * exp(- muzero/alphaG(d,tao) *(exp (alphaG(d,tao)*(i - tao)) - 1) - beta_gam * sum(log(A_hldr(tao: twalk: i))) ) + holder_inf; %IMMATURE GAMETOCYTES                                                                     end                                                         %IMMATURE GAMETOCYTES                              G(d,i) = holder;                             holder = 0; %resets the dummy                                                                       inf_G(d,i) = holder_inf;                                    %IMMATURE GAMETOCYTES                                                holder_inf = 0; %resets the dummy                           %IMMATURE GAMETOCYTES                                                                             if G(d,i) < minparasitemia                                              G(d,i) = mingamval;                             end                                                           if inf_G(d,i) < minparasitemia                                                 %IMMATURE GAMETOCYTES                                                   inf_G(d,i) = mingamval;                                                     %IMMATURE GAMETOCYTES                             end                                                                            %IMMATURE GAMETOCYTES                                                      end %This is the end of the i > delay(d) loop                 %% Calculate Gametocytemia Assuming Transmission Blocking                       end %End of the time loop                 end %End of the treatment if/else statement                 d                % progressbar(d/numofruns)                        %% Calculate the fitting values gbar and L                 for i = begtm:twalk:(tstar(d)-delay(d))                         Astar(d) = A(i) + Astar(d);                end                 for i = begtm:twalk:(tstar(d)-delay(d))                      gprod(d) = (gamma(d,i)*A(i)) + gprod(d);                   end                 gbar(d) = gprod(d)/Astar(d);                 for i = (delay(d)):twalk:tstar(d)                         L(d) = G(d,i) + L(d);                end                 L(d)= L(d)/gprod(d);                 %************************************************************                 for i = begtm:twalk:(posday(d)-delay(d)) %'true' in the sense of model-predicted, not measured                        trueAstar(d) = A(i) + trueAstar(d);                end                 for i = begtm:twalk:(posday(d)-delay(d))                        truegbar(d) = (gamma(d,i)*A(i)) + truegbar(d);                    end                 truegbar(d)=truegbar(d)/trueAstar(d);                 for i = begtm:twalk:(posday(d)-delay(d))                        trueGstar(d) = (gamma(d,i)*A(i)) + trueGstar(d);                    end                 for i = (delay(d)):twalk:posday(d)                         trueL(d) = G(d,i) + trueL(d);                    end                 trueL(d)= trueL(d)/trueGstar(d);                 clear dummy holder i s t tao %(counter peaks Ptotshift) are cleared by prefetch     end %End of the number of runs loop %% Introduction of Poisson Errors (see Molineaux, et al., 2001, pg. 383)    %Recall that this ditribution can take only integer values; thus we have    %restricted the range of possible outputs (nothing less than 1/microliter).     %{    for z  = numoferrors_gam;        for d = 1:numofruns        for i = initval:tstep:endtm               if G(d,i) <= Poissonthresh_gam                           Gpoisson(z,d,i) = random('poiss',G(d,i),1);   %To use this matrix for data analysis, use Ptotpoisson(z,d,:);                                                                     %this gives the zth error-corrected result from the dth run                        else                       Gpoisson(z,d,i) = random('poiss',Poissonthresh,1)*G(d,i);                       end                     end      end    end    %} %% Calculation of First Observable Gametocyte Day     for d = 1:numofruns           counter = 0; i = 1;            while (counter == 0) && (i <= endtm)            if G(d,i)>=detectionthresh;                gamobsday(d) = i;                counter = 1;            else                i = i+1;            end        end    end %% Infectivity Model    for d = 1:numofruns;%% Fever Simulation          for i = 1:fevernumber                                  cntr = 0;                                  for j = 1:(feverduration+1)                     feverset(d, ( j + (i-1)*(feverduration+1)) ) = oldfeverday(d) + cntr + (i-1)*feverperiodicity; % changed from feverday to oldfeverday to account for treatment                                 cntr = cntr + 1;                                     end                         end             %% Infectivity Simulation                             for t = begtm:twalk:endtm %For the entire period of Gametocyte modeling                            if strcmp(infect_fn,'JefferyEyles')                        JefferyEyles = @(x) 0 + 1.08*exp(-exp (-.86*(log10(x) - 1.48) ) );                                              if ismember(t, feverset(d,:))                                c(d,t) = medfevereffect*JefferyEyles(G(d,t));                                    else                                           c(d,t) = JefferyEyles(G(d,t));                        end                                end % End of the JeffereyEyles loop                 if strcmp(infect_fn,'Drakeley')                    Drakeley = @(x) .07 + .38*exp(-exp (-4.09*(log10(x) - 2.09) ) );                                        if ismember(t, feverset(d,:))                                       c(d,t) = medfevereffect*Drakeley(G(d,t));                                   else                        c(d,t) = Drakeley(G(d,t));                                        end                                end % End of the Drakeley loop %% Minimum Gametocyte Level    if G(d,t) < mintrans                      c(d,t) = 0;    end%% Max Infectivity Level    if c(d,t) > maxinfectivity              c(d,t) = 1;    end%% Minimum Infectivity Level    %if c(d,t) < mininfectivity          %c(d,t) = 0;    %end         end % This is the end of the for time loop           end % This is the end of the d number of runs loop %% Jeffery & Eyles Effect     for d = 1:numofruns;        if ((gamobsday(d) - firstobs(d)) <= 15 ) && (gamobsday(d)~=0)            for i = 1:gamobsday(d) + notrans;                                               c(d,i) = 0;                           end        else            for i = 1:(firstobs(d) + JEwaittime);                                              c(d,i) = 0;                                 end        end    end %% Calculate infectivity days per person      for i = 1:numofruns           pp(i,:) = interp1([1:endtm],c(i,1:endtm),'pchip','pp');        %pp(i,:) = interp1([1:endtm],Pinterp(i,1:endtm),'pchip','pp');       end     endinttime = endtm;    for i = 1:numofruns           int(i) = quadgk(@(x)ppval(pp(i,:),x),1,endinttime);         end     mean(int);        %prctile(int,[5 50 95])    %CAN ALSO USE trapz(c(i,:))    %% Prepare for export and export data (use Mat format for now)     tElapsed = toc;     %IMMATURE GAMETOCYTES    s = ['save new_data_' sprintf('%d',lambda) ' DHA_set prtnrdrug_set factor_inf inf_G Pfstar Ptot Pmstar Ptotstar Pinterp posday tstep initval numofruns firstobs tstar minparasitemia treatparasit fstdruglevels prtnrdruglevels probtreat randval treatmentwait treatmentcourse_fstdg treatmentcourse_prtnrdrug treated_asexual twalk A G gamma delay gbar L begtm truegbar trueL twalk endtm numofruns feverday c numofruns feverset seed stream alphaG pp int oldfeverday m P repeatcounter total_real_P total_real_DHA selection_set tElapsed factor;'];    %IMMATURE GAMETOCYTES        eval(s)                if strcmp(drug,'LMF')                currentFile = sprintf('drug_output_%d.mat',lambda);                if rand_PK == 1                    save(currentFile,'ka_holder','alpha_holder','V_holder','F1_holder','F2_holder','F3_holder','F4_holder','F5_holder','F6_holder','mn_PD','Cdaily_drug','drug','upd_incrmnt','total_conc','upd_incrmnt','x_C_lum','Cdaily_drug_standard','numofruns_PKPD','rand_mult_holder_F','rand_mult_holder_Ka','rand_mult_holder_alpha','rand_mult_holder_V')                end            end                                   if strcmp(drug,'CQ')                currentFile = sprintf('drug_output_%d.mat',lambda);                if rand_PK == 1                    term_half_life = log(1/2)/(k_1_2_2/24) .* rand_mult_holder_t_clear_term;                      save(currentFile,'mn_PD','Cdaily_drug','drug','k_1_2_1','k_1_2_2','total_conc','x_C_cq','Cdaily_drug_standard','CQ_interp','mdCQ_interp','rand_mult_holder_tmax','rand_mult_holder_Cmax','rand_mult_holder_t_clear_init','rand_mult_holder_t_clear_term')                end            end                        if strcmp(drug,'MEF')                currentFile = sprintf('drug_output_%d.mat',lambda);                if rand_PK == 1                    term_half_life = log(1/2)/(k_1_2_2/24) .* rand_mult_holder_t_clear_term;                    save(currentFile,'mn_PD','Cdaily_drug','drug','k_1_2_1','k_1_2_2','total_conc','x_C_mef','Cdaily_drug_standard','rand_mult_holder_tmax','rand_mult_holder_Cmax','rand_mult_holder_t_clear_init','rand_mult_holder_t_clear_term')                end            end     % clear all     disp('The program has finished.')     %usefulmetrics end    
