# Supplementary figures and images for: Modeling Within-Host Effects of Drugs on Plasmodium falciparum Transmission and Prospects for Malaria Elimination
Source: PLoS Comput Biol. 2014 Jan 23;10(1):e1003434. doi: 10.1371/journal.pcbi.1003434 (PMC3900379; doi:10.1371/journal.pcbi.1003434)

Figure S1

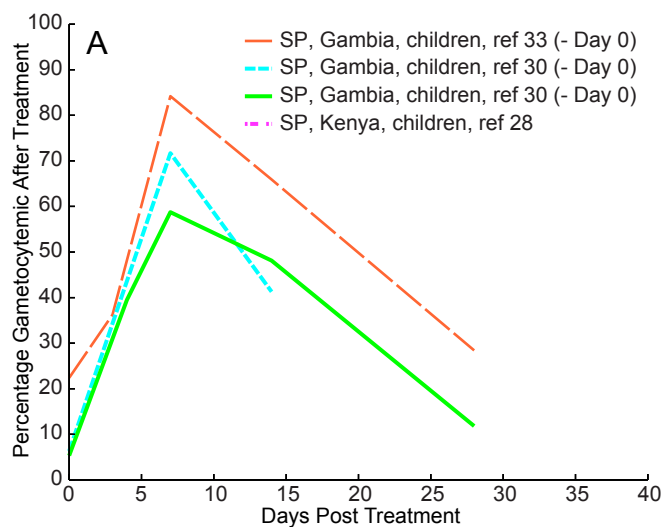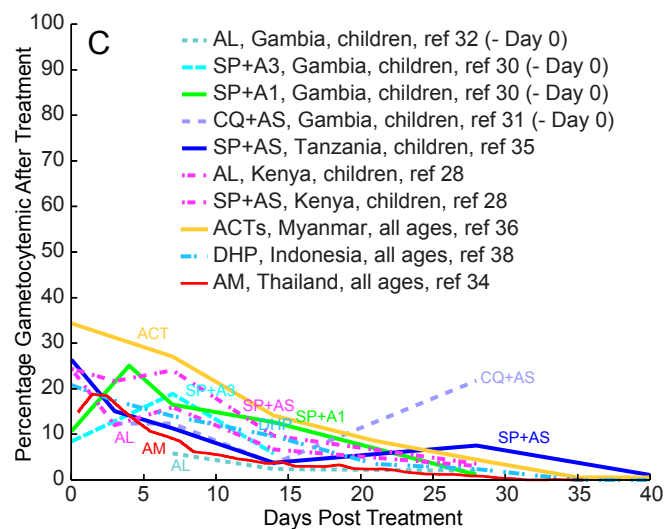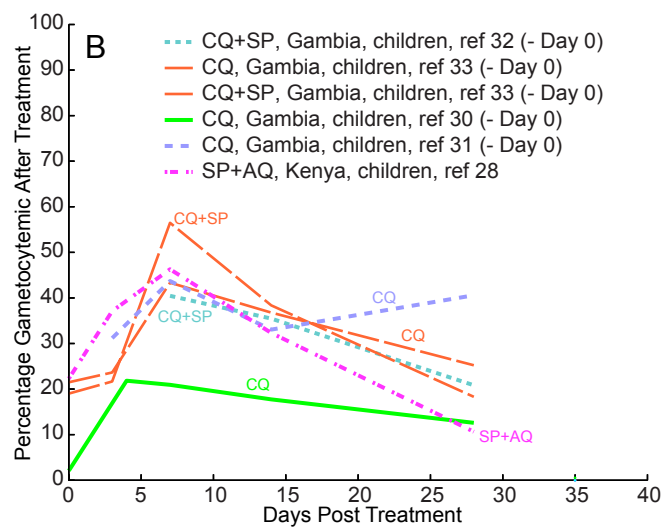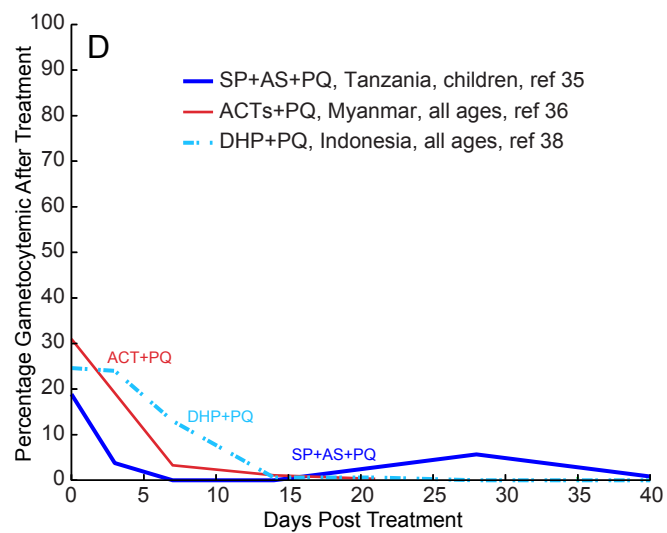

Supplement: Figure S1 — Post-treatment gametocyte prevalences from field studies. The graphs show gametocyte prevalences of field populations after antimalarial treatment. Gametocyte positivity was assessed using microscopy (threshold ∼5–10 gametocytes per µL blood). The notation (‘− Day 0’) indicates that only individuals who were gametocyte negative at admission were included in the study. Field notes include the location of study and subset of population treated. All study curves represent mean population values linearly interpolated from measured prevalences. (A) The percentage of individuals positive for gametocytes after treatment with sulfadoxine-pyrimethamine (SP) [28], [30], [33]. The pattern after SP treatment can be described as an inverted-V: few mature gametocytes were present at treatment because treatment was relatively prompt and some studies excluded gametocyte carriers at admission. A peak in prevalence was caused by sequestered gametocytes emerging into the blood stream. The peak gradually declined as the immune system cleared gametocytes from the blood. (B) Gametocyte prevalences after treatment with chloroquine (CQ) or amodiaquine (AQ) (sometimes in combination with SP) [28], [30]–[33]. (C) Gametocyte prevalences after treatment with various artemisinin-based combination therapies (ACTs): (artemether-lumefantrine, AL), (artesunate, A1, A3, AS), (artesunate-mefloquine, AM), (dihydroartemisinin-piperaquine, DHP) [28], [30]–[32], [34]–[36], [38]. (D) Gametocyte prevalences after treatment with ACTs plus primaquine (PQ) [35], [36], [38]. (PDF) [file pcbi.1003434.s002.pdf]

Figure S2

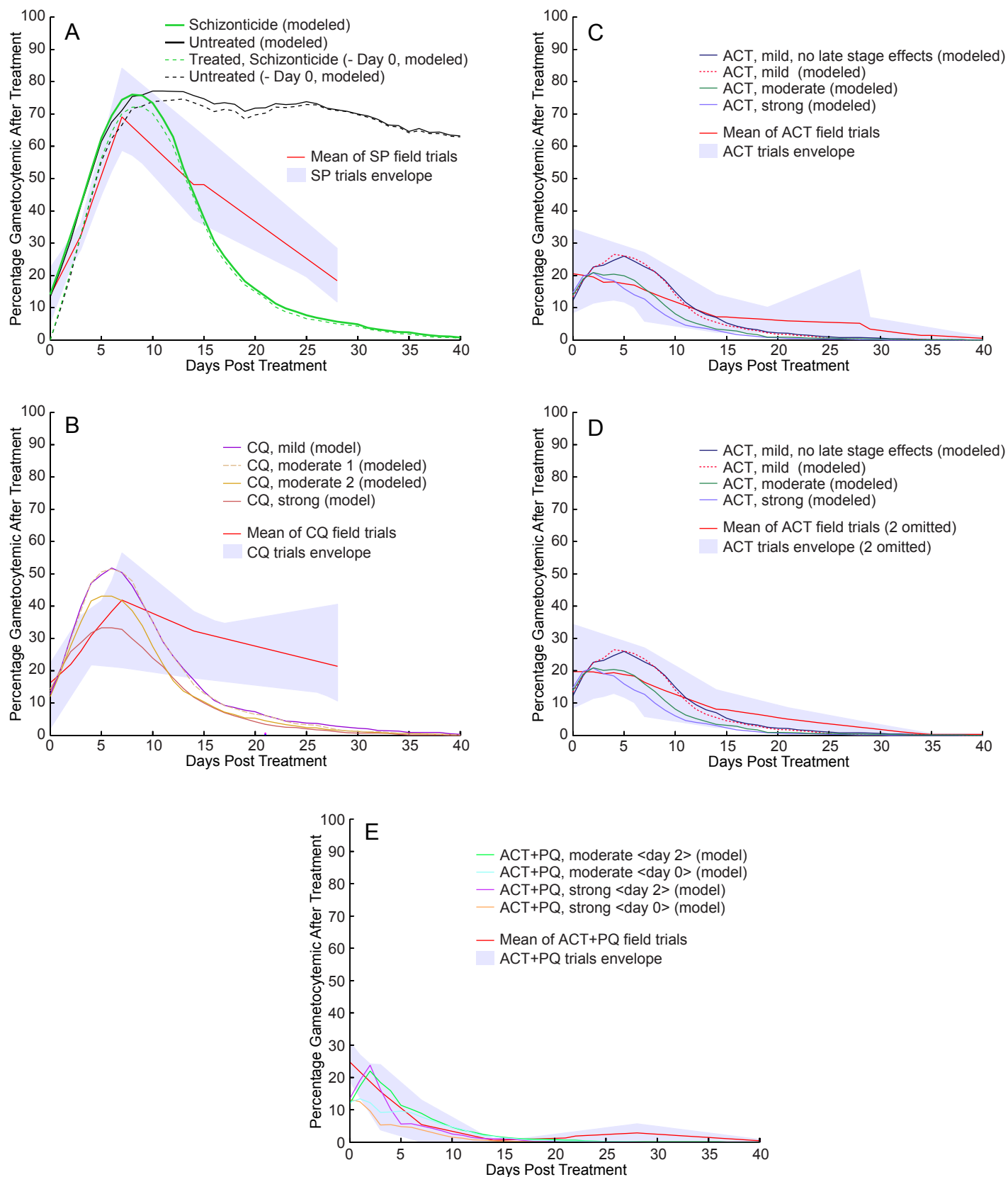

Supplement: Figure S2 — Comparison of modeled post-treatment gametocyte prevalences to field study data. The post-treatment gametocyte prevalences from Figure S1 were averaged to create a set of target data to parameterize the modeled effects of antimalarials on transmission. (A) The mean of the field data after treatment with sulfadoxine-pyrimethamine (SP) [28], [30], [33] is illustrated by the red line and the range of observed responses are depicted in light blue. The modeled gametocyte carriage curves are shown in black and green. All model treatment was assumed to start 5 days after the first onset of fever, consistent with average behavior from field studies [34], [38]. All model outputs represent the mean of 1,000 runs. The solid black line illustrates modeled gametocyte carriage among untreated individuals; the dashed black line illustrates modeled clearance in untreated individuals among gametocyte negatives at admission (‘- Day 0’). Modeled gametocyte clearance in untreated individuals is mediated only by immune processes as described in [23]. The solid green line depicts modeled gametocyte prevalence after treatment with a schizonticidal combination therapy (i.e. a short-lived component that rapidly kills asexual parasites and a longer-lived one that is less potent; neither are assumed to affect gametocytes); the dashed green line depicts modeled gametocyte carriage after schizonticidal treatment, including only gametocyte negative individuals at treatment (‘- Day 0’). (B) Gametocyte prevalences after treatment with chloroquine (CQ) or amodiaquine (AQ) (sometimes in combination with SP) [28], [30]–[33]; mean values are illustrated in red, range in blue. Modeled gametocyte prevalences are also provided. Model prevalences assume treatment with a combination of drugs (short-lived and long-lived) that kill asexual parasites but only affect early stage gametocytes. Mild, moderate (2 being stronger than 1), and strong model outputs vary in the assumed intensity of early stage gametocy [file pcbi.1003434.s003.pdf]

Figure S3

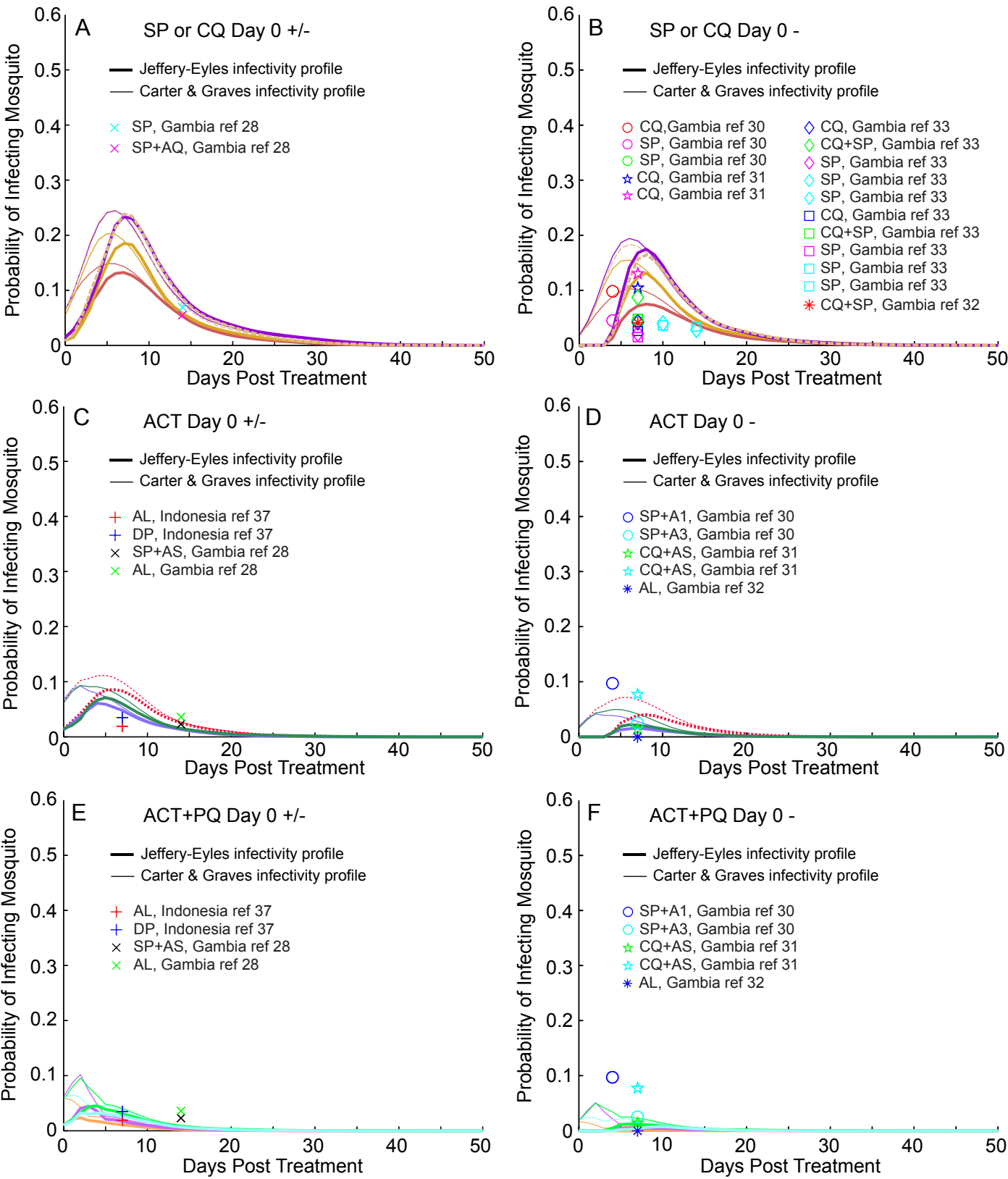

Supplement: Figure S3 — Infectivity to mosquitoes after treatment. These graphs illustrate the probability that a human will infect a mosquito following antimalarial treatment. Infectivity is defined as the probability that a mosquito bite will produce oocysts. Field study data are indicated with markers; model outputs are indicated by curves. All model outputs represent the mean of 1,000 runs; treatment was assumed to begin 5 days after first fever. Field markers represent the mean from a set of mosquito feedings. Model output curve coloring is taken from Figure S1. Two different gametocyte density-to-infectivity relationships were used to model infectivity: Jeffery-Eyles (in bold; JE) and Carter & Graves (CG) [23]. Some field and model data included only gametocyte negative individuals at admission, as indicated by (‘Day 0 −’); others included all individuals (‘Day 0 +/−’). (A) Field data post-treatment with SP (sulfadoxine-pyrimethamine) or SP plus amodiaquine (AQ) [28]. The modeled outputs are from simulations approximating the effects of chloroquine (CQ) treatment. Field-measured infectivity after SP treatment resembles that of modeled CQ treatment, even though gametocyte densities after SP treatment were much higher than after CQ. The discrepancy is explained in part by evidence that SP acts against the mosquito stages of development, thus reducing the human-to-mosquito infectivity for given levels of gametocytemia [68]–[70]. (B) Field-measured infectivity after treatment with CQ, SP, or CQ+SP [30]–[33]. Model outputs were normalized to remove simulated individuals positive at treatment. The JE parameterization is more consistent with field data, although it is unclear how infectious individuals were 0–3 days post-treatment. (C) Field-measured infectivity [28], [37] and modeled outputs after treatment with artemisinin-based combination therapies (ACTs), including only individuals that were gametocyte negative at admission. (D) Field-measured infectivity [30]–[32] and modeled outputs [file pcbi.1003434.s004.pdf]

Figure S4

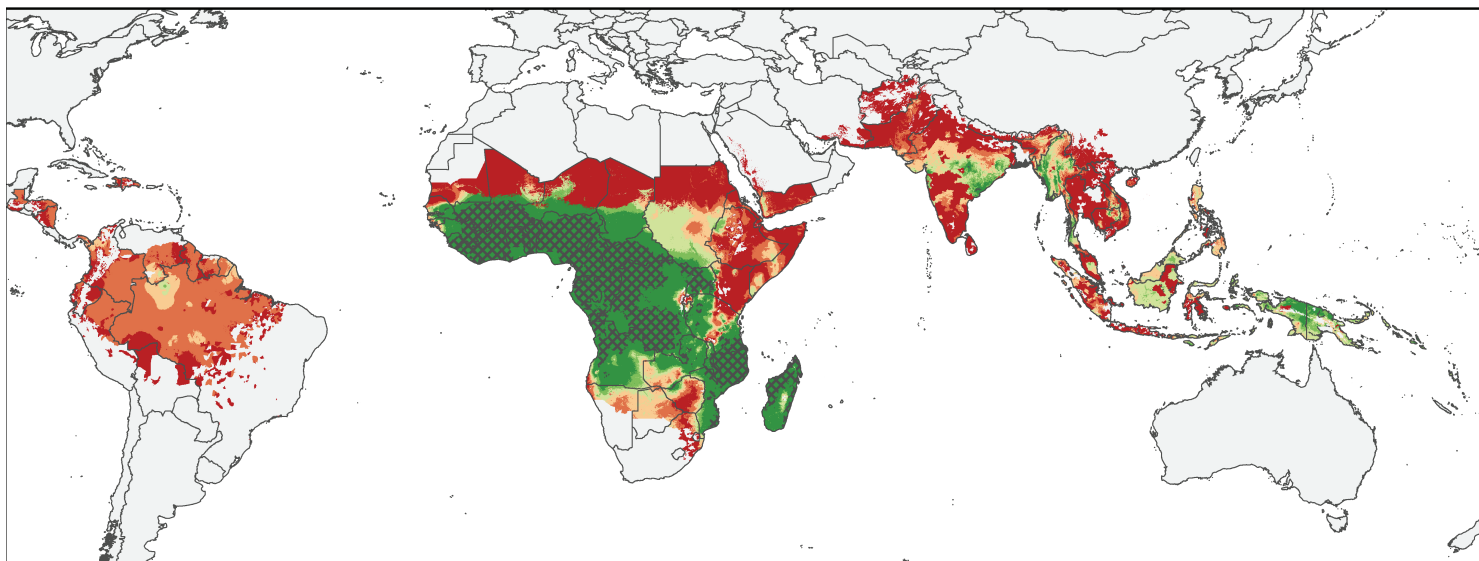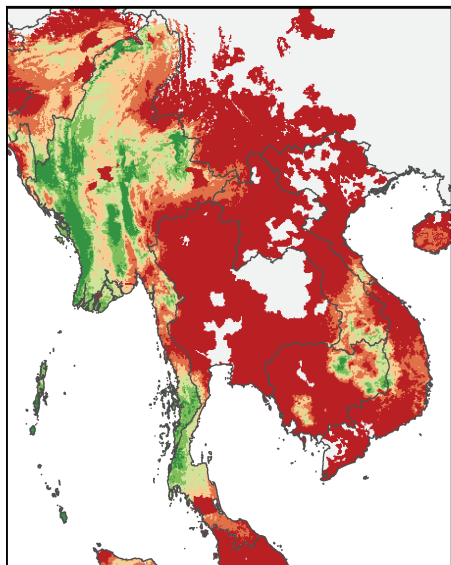

Probability of reducing  $R_C$   
to  $< 1$  with effect size of 2

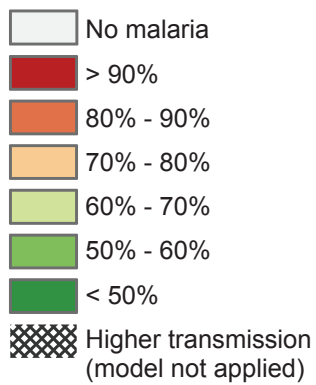

Supplement: Figure S4 — Maps of the predicted probabilities that a two-fold effect size will interrupt malaria transmission. The upper map shows the predicted probabilities that a two-fold reduction in transmission (‘two-fold effect size’) would interrupt malaria transmission over a given pixel. Map pixel size is 5 km2. In order to interrupt malaria transmission in a given area, the basic reproductive number for malaria under control (RC) needs to be reduced below 1. Probabilities for each pixel are calculated according to Bayesian posterior estimates of uncertainty (45). Probabilities have been binned into six categories for clarity. Areas with high transmission (R0>10) are masked because our model results are applicable to regions of relatively lower transmission. Note that local conditions (within a given pixel) may be more or less favorable to transmission than the per-pixel averages shown here, and so these maps are most applicable for regional or country-level planning, rather than local-level control efforts. Microenvironments or ‘hotspots’ might require additional interventions and/or greater treatment coverage than the pixel average [67]. The lower map inset illustrates the predicted probabilities that a control effort with a two-fold reduction would interrupt transmission in Southeast Asia, using the same masking of high transmission areas (R0>10) and mapping assumptions as for the upper map. Areas that appear to be uniform may have small-scale heterogeneities in transmission that are beyond the scale of this map. (PDF) [file pcbi.1003434.s005.pdf]

Figure S5

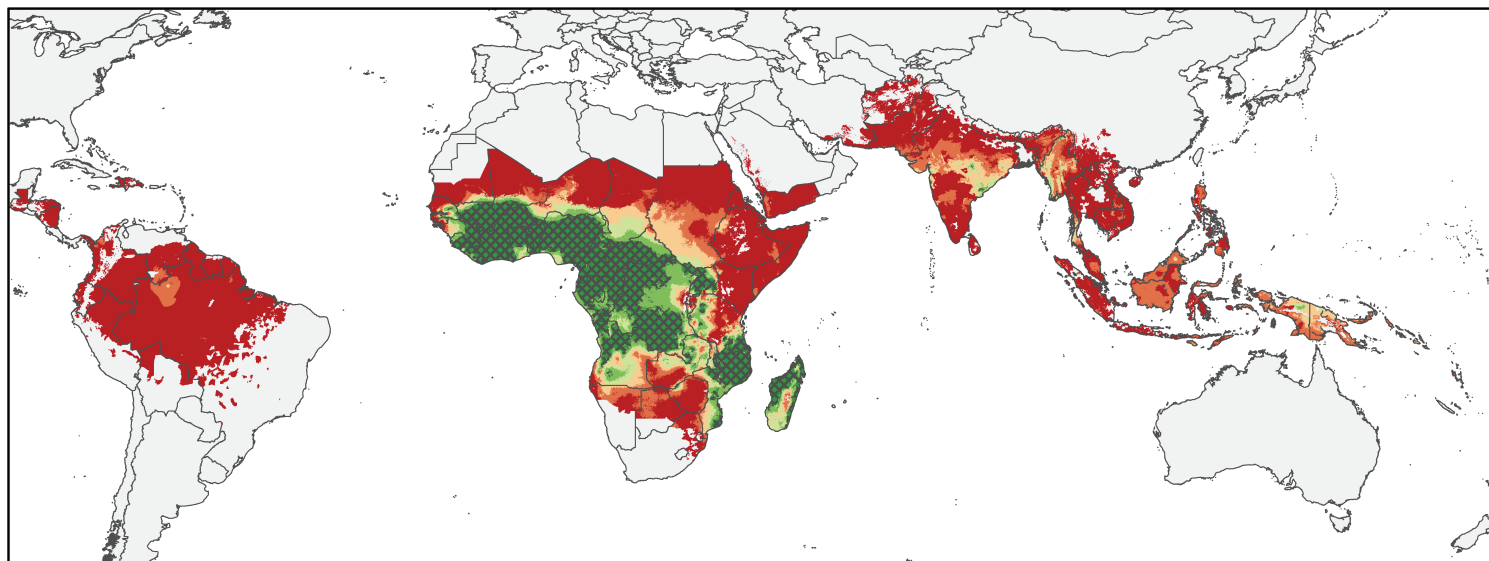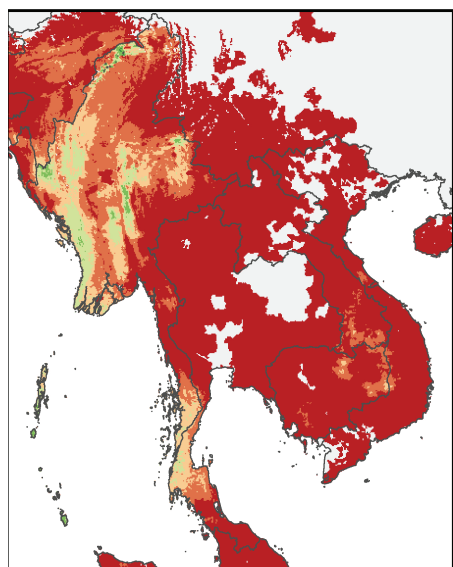

Probability of reducing  $R_C$   
to  $< 1$  with effect size of 5

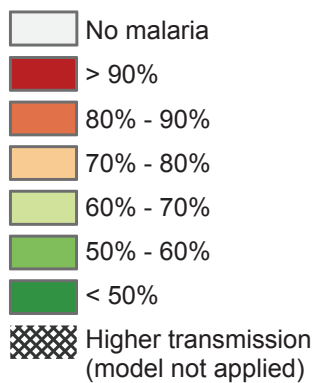

Supplement: Figure S5 — Maps of the predicted probabilities that a ten-fold effect size will interrupt malaria transmission. The upper and lower maps are illustrated as per Figure S4, except that Figure S5 shows the predicted probabilities that a ten-fold reduction in transmission (‘ten-fold effect size’) would interrupt malaria transmission over a given pixel (size is 5 km2). (PDF) [file pcbi.1003434.s006.pdf]
