# Supplementary material for: Modeling Within-Host Effects of Drugs on Plasmodium falciparum Transmission and Prospects for Malaria Elimination
Source: PLoS Comput Biol. 2014 Jan 23;10(1):e1003434. doi: 10.1371/journal.pcbi.1003434 (PMC3900379; doi:10.1371/journal.pcbi.1003434)
Supplement: Text S1 — Pharmacokinetic and pharmacodynamic equations and distributions [71]–[90]. (PDF) [file pcbi.1003434.s007.pdf]

Text S1 – Supporting Information

Modeling Within-Host Effects of Drugs on Transmission and Prospects for Malaria Elimination

Geoffrey L. Johnston, Peter W. Gething, Simon I. Hay, David L. Smith, and David A. Fidock

Table of Contents

I) Pharmacokinetics: choice of model studies: page 2

- a. Artemisinins: page 2
- b. Lumefantrine: page 2
- c. Mefloquine: page 4
- d. Chloroquine and monodesethyl-chloroquine: page 5

II) Pharmacokinetic model assumptions: page 6

- a. Equations determining pharmacokinetics: page 6

III) Pharmacokinetic variability: page 7

- a. Lumefantrine: page 7
- b. Chloroquine and monodesethyl-chloroquine: page 9
- c. Mefloquine: page 10

IV) Pharmacodynamic properties in asexual blood stage parasites: page 11

V) Fitting modeled effects of drugs on gametocytes: page 13

VI) Comparing predicted human-to-mosquito infectivity to field data: page 17

VII) Coding specifications: page 20

I) Pharmacokinetics: choice of model studies

a. Artemisinins

For all artemisinin-based combination therapies (ACTs), in our simulations we assume that artemether and/or artesunate as well as their active metabolite dihydroartemisinin are present at relevant concentrations for only the days on which they are consumed. This is due to the fact that these artemisinin derivatives are rapidly absorbed [71] and very quickly eliminated ($t_{1/2}$: 0.5 – 3 hr) [72,73]. We thus do not explicitly model the plasma concentrations of this component, as clearance of pharmacodynamically relevant concentrations occurs within the smallest time step of the model (1 day).

b. Lumefantrine

The pharmacokinetic profile of lumefantrine (LMF) differs from that of the artemisinins. Clinical studies have found that LMF pharmacokinetics follow a two-compartment model with first-order absorption and a lag time of approximately two hrs between ingestion and onset of absorption [72]. Lumefantrine is cleared much more slowly than the artemisinins (the mean terminal half-life ranges from 30 to 87 hrs) [72]. Due to this much-longer half-life, we explicitly model LMF plasma concentrations.

The standard adult prescription regimen of artemether-lumefantrine (AL) is four doses of a fixed dose tablet containing 20 mg artemether and 120 mg of LMF twice daily for three days. This AL regimen is assumed in our model. For the pharmacokinetics of LMF, we use a two-compartment model fitted to plasma concentrations from Thai patients infected with *P. falciparum* and treated with AL [61]. The current dosage recommendations correspond to the reference ‘regimen B.’

For regimen B, 18 Thai patients hospitalized in Bangkok were paired with 72 community-based patients from Mae La, and pharmacological modeling was performed to fit the plasma LMF measurements. The maximum concentration (C_{max}) achieved was approximately 7,000 ng/ml for the patients from Bangkok and approximately 8,000 ng/ml for the patients from Mae La. Because the pharmacokinetic parameters differed only slightly between the two populations, we utilized the values from Mae La. Two other Thai studies yielding plasma LMF concentrations near these values are Ezzet et al. [71] (although this study examined a two day regimen rather than three) and van Vugt et al. [74] (with pharmacokinetic parameters as reported in White et al. [72]).

While these studies have similar pharmacokinetic profiles, some other studies report much higher LMF plasma concentrations. In one study, 219 patients with acute, uncomplicated *P. falciparum* malaria in Thailand were treated with AL, and LMF concentrations were monitored during treatment [75]. In a second study, LMF concentrations were monitored among 14 Western Europeans without malaria but treated with AL [76]. In both studies, the mean C_{max} was much higher than in the set of trials above: ≥ 25,700 and 28,300 ng/ml, respectively. Further, the terminal half-life reported in the latter study was relatively long (11.5 days vs 3.1 and 4.5 for two Thai studies) [72]. The higher values for the latter study were to be expected: LMF levels are lower among individuals who are infected with *P. falciparum*, and in this study the medications were taken with a standard diet (ensuring adequate lipid consumption). The former study provides a slight outlier, as these were infected Thai individuals. However, since their plasma concentrations were not followed after treatment, few pharmacodynamic implications can be drawn.

Thus, given the applicability of these various studies to treatment of malarious individuals in low transmission settings, our LMF modeling utilizes parameters from Ezzet and van Vugt et al. [61].

c. **Mefloquine**

For the initial phase of the pharmacokinetic profile of mefloquine (MFQ), we utilized a recent study in which adults presenting with *P. falciparum* at the Hospital for Tropical Diseases, Bangkok, Thailand were treated with either a fixed dose (n = 25) or a loose dose (n = 25) formulation of artemether-mefloquine (AM) [62]. The fixed dose formulation consists of two fixed dose tablets containing 100 mg artesunate and 200 mg of MFQ daily for 3 days. The loose dose formulation is dosed by weight and consists of non-fixed artesunate at 4 mg/kg of body weight/day for 3 days plus MFQ at 15 mg/kg on day 1 and 10 mg/kg on day 2. These individuals' MFQ plasma concentrations were followed for 28 days.

We used a different study for estimation of the terminal half-life of MFQ. In this study, 39 Peruvian adults infected with *P. falciparum* were treated with a loose dose formulation of AM and their MFQ plasma concentrations were monitored for 56 days [63]. The terminal half-life was calculated (utilizing concentrations at days 21, 28, 35, 42, and 56) as 347 hours, compared to 286 and 322 for the fixed and loose regimens in Thailand, respectively. MFQ pharmacodynamics are often described by a two-component model [77] and because the Thai half-life estimates come from earlier measurements, they might not represent the true terminal half-life but rather a combination of initial and terminal half-lives. Further, the Peruvian study measures concentrations in whole blood rather than plasma (i.e. it includes MFQ bound to and/or absorbed into red blood cells). Because the parasite lives within red blood cells, whole blood concentrations are relevant for our study of the effects of drugs on malaria. In neither the Thai nor the Peruvian study were the compartmental model parameters reported.

Once the datasets had been selected, we extracted the MFQ plasma concentrations for days 0–28 from the Thai data published by Krudsood et al. [62] using data-mining software [78]. To

extrapolate plasma MFQ concentrations past day 28, we utilized the terminal half-life from the Peruvian study [63]. Our model thus allows for simulation of MFQ concentrations at any day during and after treatment for both loose and fixed formulations and utilizes the most reliable data from both studies.

d. Chloroquine and monodesethyl-chloroquine

For the pharmacokinetics of chloroquine (CQ), we relied upon a study that tracked the plasma concentrations of CQ and one of its active metabolites, monodesethyl-chloroquine (md-CQ), over time in Melanesian children with malaria [60].

For our CQ modeling, we used the children (aged 5 – 10) in arm B, who were treated with 10 mg base/kg daily for three days, along with a single dose of sulfadoxine-pyrimethamine (SP) with the first CQ dose. We assume that there are no pharmacokinetic interactions between these drugs. Given that average child weight was 18.8 kg, the dosing regimen yields $10 \text{ mg/kg} \cdot 3 \cdot 18.8\text{kg} = 564$ mg of CQ base on average. The suggested dose of CQ for adults is 1.5 g of CQ over two days; because we are modeling the concentrations in adults, we thus scaled the observed plasma concentrations of both CQ and md-CQ by 1.5 / 0.564.

To extract the plasma concentrations from the paper, we used the same technique as for MFQ, i.e., we digitized the relevant figures and extracted the data using [78]. Of note, both MFQ and CQ are eliminated fairly rapidly at active concentrations but then have a slow terminal clearance rate at sub-active concentrations. To address this, we used two different parameters to model drug clearance: an initial half-life and a terminal half-life. The transition point between the two phases was chosen qualitatively based on the observed clearance patterns from published pharmacokinetic studies [60,62,63].

II) Pharmacokinetic model assumptions

a. Equations determining pharmacokinetics

The plasma concentrations of LMF in the central compartment are given by the equation

$$C(t) = \begin{cases} \sum_{i=1}^{n-1} D_i \left[Ae^{-\alpha\left(t-t_{D_i}-Tlag\right)} + Be^{-\beta\left(t-t_{D_i}-Tlag\right)} - (A+B)e^{-k_a\left(t-t_{D_i}-Tlag\right)} \right] & \text{if } t - t_{D_n} \leq Tlag \\ \sum_{i=1}^{n} D_i \left[Ae^{-\alpha\left(t-t_{D_i}-Tlag\right)} + Be^{-\beta\left(t-t_{D_i}-Tlag\right)} - (A+B)e^{-k_a\left(t-t_{D_i}-\mathrm{T}lag\right)} \right] & \text{if not} \end{cases}$$

The parameters for this model are given in [61,79] and are reproduced below:

Parameter	Value	Description
Ka	rand_mult_Ka .* 0.17	absorption constant
alpha	rand_mult_alpha .* 0.114	initial plasma phase elimination constant
beta	0.009	terminal plasma phase elimination constant
k21	0.015	rate from peripheral to central compartment
V	rand_mult_V .* 103	volume of distribution of the central compartment
L	[0 8 24 32 48 56]	dosage times (in hours); Coartem dosing card
Lag	2	lag time (in hours)
F1	rand_mult_F .* (1.0) *(4*120*1000)	bioavailability
F2	rand_mult_F .* (.51) *(4*120*1000)	bioavailability
F3	rand_mult_F .* (1.49) *(4*120*1000)	bioavailability
F4	rand_mult_F .* (.51) *(4*120*1000)	bioavailability
F5	rand_mult_F .* (2.54) *(4*120*1000)	bioavailability; 0 for four dose regimen
F6	rand_mult_F .* (1.68) *(4*120*1000)	bioavailability; 0 for four dose regimen

The pharmacokinetic conversion to standard form parameters are given by [79]:

Parameter	Equation
A	(ka/V)*(k21-alpha)/((ka-alpha)*(beta-alpha))
B	(ka/V)*(k21-beta)/((ka-beta)*(alpha-beta))

These parameters can also be found in the model source code. The stochastic values, rand_mult_Ka, rand_mult_alpha, rand_mult_V, and rand_mult_F, were chosen to match the observed C_{max} and area under the curve (AUC) values from [61]. The parameters reported in [61] were calculated using NLME, a nonlinear mixed effect algorithm.

For MFQ and CQ, we did not use an explicit compartmental model; rather we used the measured plasma concentrations from selected studies and interpolated using log-linear interpolation. For time points where we could not interpolate plasma concentrations, we used the terminal plasma concentration half-lives to extrapolate these values.

III) Pharmacokinetic variability

a. Lumefantrine

For the pharmacokinetic variability of LMF, we assumed that individuals differed in their rates of drug uptake and clearance. Specifically, we assumed that individuals varied in terms of LMF bioavailability (fraction of drug absorbed in each dose), volume of distribution, absorption rate, and initial plasma clearance rate (rand_mult_F, rand_mult_V, rand_mult_Ka, and rand_mult_alpha, respectively). To model this variation, we assumed that each of these four parameters varied independently within a population. Each individual was simulated as a draw from the set of four probability distributions for these four pharmacokinetic properties. Once this draw was chosen, we then multiplied the population medians [61] for these four pharmacokinetic indices by each of the four individual-specific values and computed the plasma concentrations over time using two-compartment model equations as described in [79].

The four probability distributions were chosen so that the variation in concentrations over time in the simulated population matched the concentrations in the observed population. Specifically, we attempted to match the C_{max} and the AUC of the modeled and observed populations (also written AUC($0 \rightarrow \infty$) or just AUC). For the distribution of bioavailability, we set half of the population to have a higher bioavailability than the median (max of 1.5-fold increase), and half to have a lower bioavailability (min of 3-fold decrease). For the volume of distribution, we set half to greater than the median (max of 1.5-fold increase), and half to lower (min of 1.5-fold decrease). For the constant of absorption, we set half to greater than the median (max of 1.5-fold increase), and half to lower (min of 1.5-fold decrease). For the initial plasma elimination constant, we set half to greater than the median (max of 1.1-fold increase), and half to lower (min of 2-fold decrease).

The resulting model output for LMF yields an AUC($0 \rightarrow \infty$) of 212 (in ng/ml/day) compared to a reported value of 561 (4). Regarding the bounds on AUC, the model 5% and 95% are 72.4 and 537.7, versus field values of 231 and 1,668, respectively (units of ng/ml/day) [61]. For C_{max} (μg/ml), the field data reports a median of 9.0 with 5% and 95% quantiles of 1.1 and 19.8 [61]. In our model, these values are 8.4 (range 2.97 – 19.3). We do not simulate the effects of parasite load on bioavailability, though high loads generally yield lower bioavailability [61].

As an additional check, at day 7 our pharmacokinetic model yields a mean concentration of 787 ng/ml for LMF, with a range of 152 – 2,739, compared to an observed mean value of 528 ng/ml (range 49-5,175 ng/ml) in a study among Karen women in Thailand [80]. Further, in our model the median number of hours until a concentration of 280 ng/ml is reached is 265.5 hours, compared to 252 hours in the field.

b. Chloroquine and monodesethyl-chloroquine

To simulate the pharmacokinetic variation for CQ, monodesethyl-CQ (mdCQ), and MFQ, we did not use a compartmental model; instead we took observed population mean values from field trials and used log-linear interpolation and extrapolation to generate daily plasma concentrations. Thus, we could not multiply compartmental model parameters to generate stochastic variation. Rather, we multiplied the observed mean values by scaling factors to generate individual variation in concentrations over time.

Specifically, for CQ we multiplied the observed time to maximal concentration (T_{max}) by a mean-preserving scaling factor between 1.3 and 0.77; we multiplied the C_{max} of half of individuals by a factor between 1 and 1.9 and the other half by a factor between 1 and 0.67; we multiplied the initial half-life of half of individuals by a factor between 1 and 1.7 and the other half remained unchanged from the population mean; and we multiplied the terminal half-life by a mean-preserving scaling factor between 1.5 and 0.67.

These distributions were chosen so that modeled and observed values would match in terms of AUC and C_{max}. For CQ, the reported median and interquartile range (IQR) of the AUC (in μM·hr) are 122.5 (range 101.1 – 165.8); see reference [60]. The model produces values of 102.4 (range 80.3 – 135.9). For the C_{max} (in nM), the reported median values are 233 (range 206 – 298); the model output yields 247 (range 203 – 291).

For mdCQ, we used the same scaling factors as for CQ. However, we assumed that the decreases in concentration in the initial half-life phase lasted for 7 days in mdCQ versus 6 days for CQ. In terms

of model fit to observed values, for mdCQ, the reported median and IQR of the AUC (in μM·hr) are 98.5 (range 69.5 – 133.8); see (12). The model produces 98.5 (range 76.3 – 132.7). For the C_{max} (in nM), the reported values are 290 (range 236 – 368); the modeled values are 248 (range 203 – 294).

c. <u>Mefloquine</u>

We used the same procedure to generate individual variation in plasma concentrations for MFQ as for CQ and mdCQ; however, the scaling constants were changed to match observed variation [62]. For a fixed dose of MFQ, we multiplied the T_{max} by a mean-preserving scaling factor between 1.55 and 0.65; we multiplied the C_{max} by a mean-preserving scaling factor between 1.9 and 0.53; we multiplied the initial half-life of half by a mean-preserving scaling factor between 1.7 and 0.59; and we multiplied the terminal half-life by a mean-preserving scaling factor between 1.9 and 0.53. We assumed that the initial plasma clearance phase lasted 8 days.

For the AUC of a fixed dose of MFQ the mean, (range), standard deviation, and coefficient of variation are 1,145,977, (519,164 – 3,103,075), 678,719, and 59.2, respectively (units of ng/ml·hr) [62]. In the model, these values are 1,216,251, (456,843 – 2,752,062), 578,381, and 47.5, respectively. For the C_{max} values (ng/ml), the mean, (range), standard deviation, and coefficient of variation are 3,279, (1,809 – 5,796), 1,252, and 38.2; for the model we have 3,044, (1,655 – 5,422), 1,142, and 37.6, respectively.

For the loose dose formulation of MFQ, we used the same stochastic parameters as for the fixed dose. For the AUC of a loose dose of MFQ the mean, (range), standard deviation, and coefficient of variation are 1,095,421, (571,397 – 2,127,627), 370,167, and 33.8, respectively (units of ng/ml·hr) [62]. In the model these values are 1,172,894, (range 476,334 – 2,531,135), 542,679, and 46.3,

respectively. For the C_{max} values (ng/ml), the mean, (range), standard deviation, and coefficient of variation are 3,239 (range 1,817 – 4,583), 734, and 22.7; for the model we have 3,207, (range 1,745 – 5,666), 1,155, and 36.0, respectively.

IV) Pharmacodynamic properties in asexual blood stage parasites

For the pharmacodynamic properties of the artemisinins, we assumed a binary model (killing/no killing), given their rapid half-lives. If multiple drugs were present at a given time point we assumed that the effects are strictly additive (i.e. no synergism).

For the dose-response indices of the partner drugs, we took known *in vitro* relationships among the drugs and scaled them by the estimated effectiveness of MFQ *in vivo*, because the effectiveness of MFQ *in vivo* has been characterized by two studies [65,66]. These studies relied upon a calculus-based method for estimating the effects of drugs on parasite growth over time [81]. For the *in vitro* relationships, we assumed that IC_{50} values (i.e. the 50% inhibitory concentrations *in vitro*) were 25 nM for CQ (8 ng/ml of the hydrochloride salt) [82], 66 nM for LMF (34.9 ng/ml of the base) [21], and 170.4 nM for MFQ (64.5 ng/ml of the hydrochloride salt) [83]. Thus, the ratio of *in vitro* IC_{50} values for CQ and MFQ was 25:170 = 0.147, and the ratio for LMF and MFQ was 66:170 = 0.387. Further, we assumed that the EC_{50} of MFQ was 350 ng/ml for fully sensitive parasites [66]. Thus, we used EC_{50} values of 51 and 136 ng/ml for CQ and LMF, respectively.

Because there is widespread resistance to MFQ throughout Southeast Asia, however, we used an EC_{50} for MFQ of 600 ng/ml, which is the *in vivo* value derived for slightly MFQ-resistant parasites [65,66]. As a simplification, we assumed that all Hill slopes are the same as that of MFQ (2.5; value taken from [65]).

The maximum parasite reduction rate (PRR) is a measure of the effectiveness of a drug. We define the maximum PRR of a drug as the fold-reduction in parasitemia due to drug after 48 hours when a drug is at maximal concentration. For example, if a drug's PRR is 10^3, then there will be approximately 1,000-fold fewer parasites after 48 hr of treatment at maximal concentration than in the absence of drug.

To determine the maximum parasite reduction ratios of MFQ, LMF, and CQ, we ran model simulations to determine the effectiveness of each drug as monotherapy and compared our results to field data. For LMF monotherapy, high clearance rates have been reported [84]; indeed, data from Novartis indicate that four-dose LMF monotherapy had 28-day uncorrected parasitological cure rates of 90-96%, averaged over patients of all ages [85]. For our model, we set the maximum PRR of LMF to 3,000; under a two-day, four-dose regimen of LMF monotherapy with treatment one day after first fever, our model yielded a 28-day clearance rate of 84%, which was somewhat lower than reported (though we assumed no prior immunity in our model) (21). For CQ, we set its PRR to be 3,000, given its known high potency. We assumed a background of no CQ resistance in order to examine the historical effects of CQ on transmission and to provide an estimate of the effects of a weakly-gametocytocidal schizonticide for comparison to the ACTs. For MFQ, we modeled the effects of 25 mg/kg monotherapy over two days; using a PRR of 300 yielded a modeled cure rate of 97%, as compared to the field rate of 91% in 1992 [86]. For dihydroartemisinin, artesunate, and artemether, we set their PRR values to 10^4, given their very high potency against asexual blood stages. These PRR values all corresponded roughly with prior estimates [87]. **Figs 2B**, **2D**, and **2E** show the effect of LMF, MFQ, and CQ concentrations on asexual parasites according to the model assumptions.

V) Fitting modeled effects of drugs on gametocytes

Figure S1 illustrates the post-treatment prevalences of gametocytemia from various field studies [28,30-38]. The review by Okell et al. [88] described the features of many of the studies conducted before 2007. Here, we added the studies that occurred since 2007 and that measured prevalence by microscopy. Studies were disaggregated based upon two factors: 1) the type of drugs used (non-ACT, ACT, and ACT plus primaquine (ACT+PQ)); and 2) whether or not individuals who were gametocytemic on admission were included in the analysis. Studies including only gametocyte-negative individuals at admission are indicated in the legend by the note '- Day 0'. Most of the studies were conducted on African children, although some were conducted on African adults or were performed outside of Africa.

Figure S1A shows the patterns of gametocyte clearance observed after treatment with the antifolate combination SP [28,30,33]. The inverted-V pattern, reflecting increased percentages of gametocytemic patients after treatment, can be explained by three factors. First, individuals were treated relatively early in the course of infection, before gametocyte levels were high. Second, the peak of the inverted-V could be caused by gametocytes emerging from sequestered sites into the venous bloodstream. The inverted-V ends when these residual gametocytes were cleared from the bloodstream by the immune system. Of note, the peak heights reflect the general lack of SP activity against gametocytes at any stage of their development.

Figure S1B illustrates the results of field trials using CQ, amodiaquine (AQ), or combinations of CQ or AQ with SP [28,30-33]. CQ and AQ have stronger effects on gametocytes than SP, killing the early stages [33]. However, CQ and AQ do not affect the later stages of development. The effects of the early stage gametocytemic activity of CQ and AQ on gametocyte carriage over time

were evident when compared to the effects of SP alone: the peak of the inverted-V decreased relative to the SP-treated case. The higher gametocyte densities by day 28 were also observable, as in the SP treated case.

Figure S1C illustrates the gametocyte clearance patterns after treatment with ACTs: AL, artemether-lumefantrine; DHP, dihydroartemisinin-piperaquine; AM, artesunate-mefloquine; AS/A1, single dose of artesunate; A3, three doses of artesunate [28,30-32,34-36,38]. Gametocyte prevalence was significantly reduced in comparison to CQ treatment; this is due to the fact that artemisinin and its derivatives are active against intermediate stages of gametocyte development and have some activity against later stages [21]. The prevalences at day 28 were near zero (except for the CQ+AS treated case, in which recrudescence may play a role [31]). **Figure S1D** illustrates the gametocyte clearance curves from three studies monitoring prevalence after treatment with ACT+PQ [35,36,38].

Once we had collated the available field data on the effects of antimalarial treatment on gametocytemia (we excluded those studies that used detection tools other than microscopy, as their data were not directly comparable), we then took the means and ranges of these data to use as our simulation targets. **Figure S2** illustrates the same field studies as in **Figure S1**, but the means of the field data are in red; light blue shading indicates the ranges. **Figure S2** also includes our within-host model outputs after various types of treatment.

Figure S2A illustrates model outputs for untreated individuals as well as individuals treated with a hypothetical combination schizonticidal treatment that was assumed to rapidly kill asexual blood stage parasites [23]. For our simulated schizonticidal combination, we used the pharmacokinetic and pharmacodynamic data from AM, assuming that the combination only affected asexual parasites.

We also assumed that treatment started relatively early in the infection, i.e., 5 days after first fever, in agreement with field studies [34,38]. All model data is from the mean of 1,000 runs. Model outputs include simulations with all individuals (solid line) as well as the subset of individuals that were gametocyte negative at admission (dotted lines). The behavior of gametocyte clearance was determined both by the immune responses of the hosts as well as the cessation of gametocyte production due to clearance of all asexual blood stage parasites after schizonticidal treatment.

The modeled behavior of gametocyte clearance after treatment with a pure schizonticide exhibited the same inverted-V pattern observed in the SP field data, while simulated untreated individuals remained infectious [23]. By truncating the onward asexual infection, gametocyte prevalence reached a peak and then decayed due to immune pressure and because fewer asexual parasites fed into the gametocyte development pathway. We note that these graphs depict the mean behavior of the population after treatment; in our model some individuals cleared their gametocytes faster than the mean, while others were slower, as determined by the distributions in [23].

While the initial slopes and peaks of the modeled and field data were similar, the model outputs differed from field data by the 28-day time point when the model predicted a low prevalence whereas the field data observed positive gametocyte prevalence. This discrepancy could be explained in part by treatment failures and reinfection within the field population. Because there was SP resistance at the time of the field studies [30,33], it is possible that some individuals did not completely clear their infections, thus producing additional gametocytes [28]. Some individuals might also have been reinfected during the course of the study [28]. In our model, we assumed that resistance had not yet arisen and that individuals were not reinfected during the 40–day post-treatment period. Another explanation would be that our model underestimated the duration that

gametocytes circulate in the bloodstream, though we fitted this parameter to malaria therapy data [23].

To fit the CQ, ACT, and ACT+PQ field data, we used our model to simulate the effects of increasingly gametocytocidal therapies on post-treatment gametocyte prevalence. We began by assuming that the AM combination treatment was slightly gametocytocidal, i.e., that treatment killed asexual parasites and early stage gametocytes. We then increased the assumed gametocyte killing power of the treatment; as we increased the killing power, the post-treatment gametocyte peak decreased in magnitude. We then assumed that the short-lived component killed both early and late stage gametocytes, but that the longer-lived component affected only earlier stages. Finally, we added a second partner drug to the AM combination, which we assumed killed both early and late stage gametocytes strongly (the simulated PQ treatment).

Figure S2B illustrates the model outputs with only early stage killing effects that best corresponded with the field data for CQ. The initial gametocyte prevalence and the height of the post-treatment peak corresponded well to field data, as in the case of the SP data. However, model gametocyte clearance was faster after the peak such that prevalence was near zero by day 28, whereas in the field data the prevalence was nearly the same as when treatment began. This effect was also noted in the case of the SP data, and the same explanations apply here.

Figure S2C illustrates the model outputs for those parameterizations with both early and late stage gametocytemic killing (in the short-lived component) that best corresponded to the ACT field data. The modeled gametocyte prevalence curves were at or below the envelope of observed responses. However, when we excluded two studies that may have been severely affected by drug resistance effects (CQ+AS, [31,89] and SP+AS [35]), the modeled curves better corresponded to the field data,

although modeled curves were still below observed data (**Figure S2D**). Possible explanations for the faster clearance of modeled gametocytemias are provided in the section describing SP fitting above.

Figure S2E illustrates the modeled gametocyte clearance curves assuming treatment with a triple combination: a short-lived drug that strongly kills early stages and mildly affects later stages, a longer-lived partner that kills only early stages, and a second single dose short-lived partner that strongly kills both early and late stages. We used the 'mild' ACT parameterization from **Figure S2C** for the first two components and varied the presumed effectiveness of the third (the PQ component). We assumed that the PQ component was only active for 3 days, as PQ has a very short elimination half-life (~8 hours) in the human host [39]. We also varied the assumed day of administration, comparing administration on the first or last days of ACT treatment (indicated with the labels <day 0> or <day 2> respectively), reflecting varied dates of administration reported in field studies [36,38,39]. In total, we chose four different parameterizations to represent the possible effects of single dose PQ on gametocyte densities (**Figure S2E**).

VI) Comparing predicted human-to-mosquito infectivity to field data

In order to compare our model-predicted human-to-mosquito infectivities to field data, we searched the literature to find field studies that that measured infectivity to mosquitoes after treatment with antimalarials. **Figure S3** illustrates a variety of post-treatment feeding study data, disaggregated by drug type and day 0 gametocyte positivity, along with model outputs [28,30-38]. The model-predicted infectivities were derived by taking the post-treatment gametocytemias from **Figure S2** and translating them using either the Jeffery-Eyles or Carter & Graves relationships (see [23] for a description of the two relationships).

Figure S3A illustrates the field data for CQ and SP treatment considering both day 0 gametocyte positive and negative individuals at admission. Unfortunately, only SP feeding studies were available; no CQ studies of this type could be found in the literature. Some sets of feeding data were relatively sparse, given the difficulty of conducting feeding studies and the need to disaggregate by drug type and day 0 positivity. **Figure S3A** also illustrates model-predicted infectivities assuming CQ treatment. Our model overestimated the infectivity of SP treated individuals under either the JE or CE assumptions. The feeding studies indicate that SP infectivity after treatment was similar to predicted CQ treatment, even with the much higher residual gametocyte densities after SP.

After these calculations were made, we searched the literature for studies of the effects of SP on gametocyte infectivity; although the literature is conflicted, two recent studies demonstrated that SP likely affects the mosquito stages of parasite development [68,69], while a third found that pyrimethamine inhibited ookinete formation (in *P. berghei*) [70]. We thus confirm the likelihood that SP inhibits ookinete/oocyte development, and observe ~4–fold reduced onward infectivity at day 14 for field SP infectivity as compared to modeled schizonticidal treatments that had no effect on mosquito stages.

Figure S3B illustrates both CQ and SP model and field data, including only day 0 gametocyte negative individuals. First, we note that the feeding studies exhibited significant variability. Observed CQ and SP infectivities overlapped, even given the higher gametocyte loads in SP treated patients, as noted above.

Many of the modeled CQ infectivity profiles fell within the envelope of observed responses. However, the CG parameterizations yielded predicted infectivity values that were consistently higher than observed in the data. Further, the JE parameterizations predicted that gametocytes were

not substantially infectious until day 5 after treatment, while the data showed that substantial infectivity was present by at least day 4 post-treatment. We also note that predicted infectivity was actually slightly greater than observed infectivity by day 14; this may indicate that the higher gametocytemias observed in the field data are due to recrudescence/reinfection.

Figure S3C illustrates the model and field infectivity data post ACT treatment, including both gametocyte-positive and -negative individuals. Both the JE and CG parameterizations overestimated infectivity at day 7 but some parameterizations aligned well with the field data at day 14. The model overestimation may be caused in part by the fact that LMF has been shown to have an oocidal effect in a variety of studies [21,70,90]. **Figure S3D** provides model and field infectivity data post ACT treatment, including only day 0 gametocyte-negative individuals. The modeled and observed infectivity over time was low: one field study with AL found no infectivity to mosquitoes among individuals treated with AL [32].

Figs. S3E and S3F illustrate the modeled infectivity of ACTs plus single dose PQ. We could not find field studies that measured the infectivity of individuals after treatment with PQ. We have provided the ACT field study data for reference; it is expected that ACT+PQ will exhibit substantially less infectivity. The modeled infectivity profiles exhibit very low predicted infectivity because of the low number of residual gametocytes. Unadjusted model estimates likely overestimate infectivity because PQ inhibits oocyst development [43,44]. However, PQ has a very short half life in the human host [39], and thus the oocidal effects of single dose PQ might operate for only a few (~3) days after administration. We account for the mosquito-stage effects of single dose PQ in our calculations in the **Results** section of the main text.

VII) Coding specifications

The model was built in MATLAB (Mathworks, Version R2012b). The model outputs include the random number generator seed values so that runs can be replicated exactly.
